# Effects of changing sea ice on marine mammals and subsistence hunters in northern Alaska from traditional knowledge interviews

**DOI:** 10.1098/rsbl.2016.0198

**Published:** 2016-08

**Authors:** Henry P. Huntington, Lori T. Quakenbush, Mark Nelson

**Affiliations:** 1 Huntington Consulting, 23834 The Clearing Drive, Eagle River, AK 99577, USA; 2 Alaska Department of Fish and Game, 1300 College Road, Fairbanks, AK 99701, USA

**Keywords:** Arctic marine mammals, sea ice, Alaska, indigenous communities, traditional knowledge

## Abstract

Marine mammals are important sources of food for indigenous residents of northern Alaska. Changing sea ice patterns affect the animals themselves as well as access to them by hunters. Documenting the traditional knowledge of Iñupiaq and Yupik hunters concerning marine mammals and sea ice makes accessible a wide range of information relevant to understanding the ecosystem to which humans belong. We interviewed hunters in 11 coastal villages from the northern Bering Sea to the Beaufort Sea. Hunters reported extensive changes in sea ice and weather that have affected the timing of marine mammal migrations, their distribution and behaviour and the efficacy of certain hunting methods. Amidst these changes, however, hunters cited offsetting technological benefits, such as more powerful and fuel-efficient outboard engines. Other concerns included potential impacts to subsistence hunting from industrial activity such as shipping and oil and gas development. While hunters have been able to adjust to some changes, continued environmental changes and increased disturbance from human activity may further challenge their ability to acquire food in the future. There are indications, however, that innovation and flexibility provide sources of resilience.

## 1. Introduction

**Figure 1.**
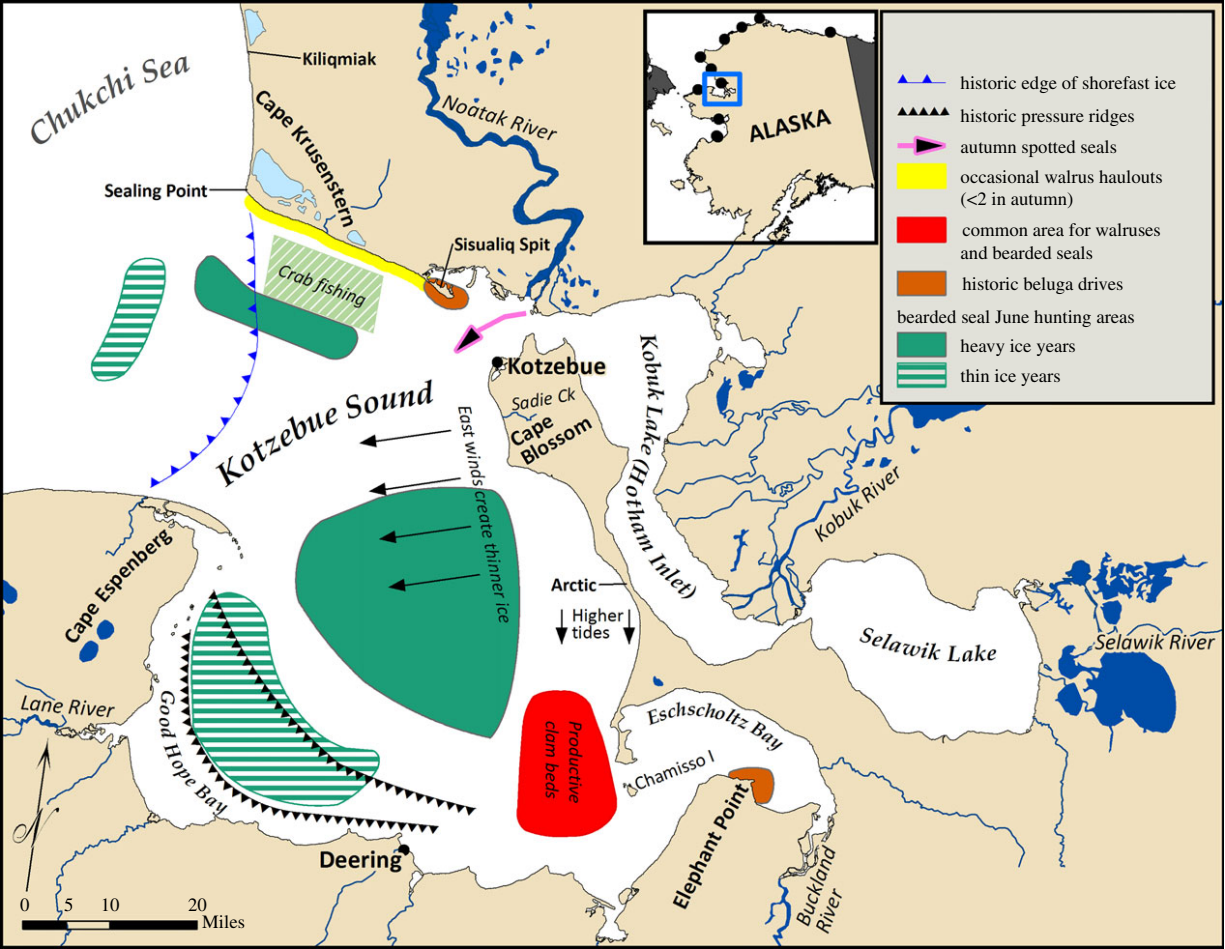
Detailed observations recorded in Kotzebue during January 2016 interviews provided as an example of the level of reported detail. Inset shows the location of the 11 communities that participated from 2007 to 2016.

Marine mammals are culturally and nutritionally vital to indigenous communities in northern Alaska, where some 80 kg of food per person per year comes from local harvests of marine mammals [1]. Safe, successful hunting requires detailed knowledge of the environment and the species hunted [2]. Sea ice, a defining characteristic of this northern marine environment [3], is changing rapidly [4], which has impacts on marine mammals and hunters [5]. Hunting is also affected by societal changes, such as economic development [6] and technology [7]. Documenting hunters' traditional knowledge provides an understanding of the implications of changing sea ice for marine mammals and hunting in northern Alaska through detailed, long-term perspectives as well as essential context in which to interpret change [8]. In this paper, we present the results of interviews with experienced marine mammal hunters in 11 Iñupiaq and Yup'ik communities from 2007 to 2016 and include a figure from our interviews in Kotzebue as an example of reported detail (figure 1 and table 1).

**Table 1.** Summary of reports. Figures in parentheses refer to the supplements to this article in which the data are found.

community	year	species focus	no. of participants
Kaktovik and Barrow (1)	2007	bowhead whales	6, 6
Wainwright (2)	2008	bowhead whales	7
Point Lay and Wainwright (3)	2011	walrus	5, 13
Point Hope (4)	2013	walrus	8
Barrow (5)	2015	walrus and ice seals	10
Elim (6)	2015	ice seals and walrus	8
St Michael and Stebbins (7)	2015	ice seals and walrus	8
Kivalina (8)	2016	ice seals, walrus, bowhead whales	5
Kotzebue (9)	2016	ice seals	6
Shishmaref (10)	2016	ice seals and walrus	5

## 2. Methods

Interview participants were selected with the assistance of the tribal government in each community as well as community representatives to various co-management organizations responsible for governance of traditional marine mammal hunting [9]. The interview method was the semi-directive interview [10], in which participants are able to follow their own trains of thought and describe ecological connections and species' behaviours as they understand them, which can lead to connections and information that interviewers did not anticipate [11]. Hunters were interviewed once, and thus their observations of change are based on their experience prior to the interview.

Traditional knowledge itself is the product of careful, systematic observation and confirmation by hunters over a lifetime, combined with what has been learned from other hunters and preceding generations [2]. It is shared among hunters with care and attention to precision and detail, as a hunter's life may depend on accurate knowledge.

The research project was approved by the responsible co-management organizations for the different species studied. In recognition of the intellectual property rights of the interview participants, free, prior and informed consent [12] was obtained from all individuals who were interviewed. Draft summaries of the interviews were sent to all participants and to the co-management organizations, to allow mistakes to be corrected, misleading or potentially harmful information removed and additional details added. Once the reviews were complete, the summaries, which constitute the data from this research (electronic supplementary material, S1–S10), were made publicly available.

## 3. Results

Notable changes in the behaviour of marine mammals include bowhead whales (*Balaena mysticetus*) arriving at Wainwright and Barrow earlier in spring and whaling near Barrow occurring earlier in the spring and later in the autumn. Hunters from Barrow and Kaktovik have noted more bowheads overall during recent decades. Less multiyear sea ice and thinner shorefast ice has made it hard to find ice on which to haul whales out for butchering in spring near Barrow and Wainwright. Bowhead whales no longer appear to feed along the edge of the shorefast ice southwest of Barrow in spring.

In recent summers, Pacific walruses (*Odobenus rosmarus divergens*) have hauled out on land near Point Lay in the tens of thousands. Formerly, they were seen on land in summer but not in large numbers. Haulouts on ice, however, are smaller (10–100 s) now than in the past, when a few thousand walruses might be seen together on large ice floes; ice floes in recent years are smaller and thinner and cannot support the weight of so many animals. Walrus body condition is good with no change observed over time.

Although ringed seals (*Pusa hispida*), bearded seals (*Erignathus barbatus*) and walruses are associated with ice, they also found also in ice-free water. Bearded seals and walruses can sleep on the water; walruses can inflate their necks to float while they sleep. Ringed seals rarely haul out on land, but bearded and spotted seals (*Phoca largha*) will; sometimes, they are seen together next to passes between barrier islands. Less snow on the ice in Norton Bay has reduced the number of ringed seal dens near Elim. There are now fewer pressure ridges around which snow drifts can form in Kotzebue Sound, though seal holes remain plentiful through the ice in spring both there and in Norton Bay. Young ringed seals were seen on beaches near Wainwright in the summer of 2010 and near Kivalina, Kotzebue and Shishmaref in recent years. These seals appeared to be ill and in poor condition. Barrow hunters reported that some bearded seals had thinner blubber in recent years, which produced different seal oil (a highly valued condiment for local foods). Old bearded seals have yellow blubber that makes yellow seal oil; this has not changed with time.

Travelling on sea ice for hunting is more dangerous and more limited now, because shorefast ice is thinner, less extensive and no longer contains multiyear ice to anchor it. The disappearance of summer sea ice also reduces hunting opportunities. Ice kept wave action down, making boating easier. Now, there are more frequent storms and fewer safe boating days. Walruses and seals are harder to find close by, because those species tend to stay with the ice, but larger boats and more efficient motors allow hunters to travel farther offshore when necessary. Walruses are more dangerous to hunt in open water. Towing them to shore for butchering takes longer and thus creates more risk than butchering them on ice. For hunters in Kivalina, Kotzebue and Shishmaref, decreases in hunting opportunity are the most notable impacts, as seals remain healthy and abundant. Spring migrations and thus hunting opportunities used to last for two months (May and June) and species were hunted sequentially. Now, migrations are compressed into a two-week or shorter period in May, so hunters must hunt earlier for multiple species in a shorter period of time. Hunters must also hunt later in the autumn when the sea ice starts to form, but more frequent autumn storms make it harder to do so.

Environmental change is not the only factor influencing hunters. There is much concern, for example, about the potential impacts of offshore oil and gas activity as well as increased commercial vessel traffic. Noise disturbs marine mammals, as hunters see from their own activities. For example, increased snowmachine (snowmobile) traffic on sea ice near Barrow is seen as one factor in decreasing numbers of whales near the edge of the shorefast ice.

## 4. Discussion

Changes in sea ice affect hunters directly by changing access and altering the utility of ice as a substrate for hunting. Changes in sea ice affect hunters indirectly by altering the distribution, timing, behaviour and local abundance of marine mammals. These effects do not occur in isolation, but as a suite of factors that combine to alter hunting behaviour and success.

These findings are consistent with other recent studies of traditional knowledge and marine mammals in the region [2,13–16] and elsewhere [5,17,18], which note both the responses of marine mammals to changing conditions and the innovations of hunters. In these studies and in ours, hunters emphasized that the impacts on animals and people are a result of the interactions among multiple factors rather than of changing sea ice alone. Assessments and studies of the overall impacts of climate change on Arctic communities similarly emphasize the combination of environmental, social, cultural, economic and technological changes that result in specific outcomes such as loss or gain of hunting opportunities [19–21].

Our results are also consistent with biological and ecological studies of Arctic marine mammals, many of which describe observed and expected negative impacts to species and those who hunt them, as well as the ways some species appear to be benefiting from less ice [22,23]. For example, there are more bowhead whales now and their body condition is improved [24]. Ringed and bearded seals in the Chukchi Sea region also appear to be abundant and healthy under recent environmental conditions [25], although there was an unidentified disease that caused weight loss, hair loss and skin sores, mostly in 2011.

Information provided by many hunters in many locations observing the environment as it changes and observing marine mammal responses to those changes is well beyond what scientists can do with research projects that are limited to one or two seasons in one location. For example, a review of the scientific literature indicates that bearded seals rarely haul out on land in Alaska [26,27]. Without local observations, scientists might continue to assume that bearded seals do not haul out on land. Bearded seal hunters, however, have seen that the seals can in fact alter their behaviour when necessary. These observations may indicate more resilience to less ice than would be known from existing scientific studies.

The changes to sea ice that have been observed to date are extensive, and greater decreases are expected in the decades to come [28]. For now, hunters have been able to adapt to some degree by improved equipment and changes in the timing of hunting. At the same time, marine mammals appear to be adjusting to the longer open water period as indicated by hunters and by biological studies. How Arctic marine mammals and their hunters will respond to even less ice will have important consequences for subsistence-based indigenous communities in northern Alaska and for the Arctic marine ecosystem. A combination of traditional knowledge and scientific knowledge is more likely to provide the information needed to prepare for the future than either approach on its own.

## Supplementary Material

Traditional Knowledge of Bowhead Whale Migratory Patterns near Kaktovik and Barrow, Alaska

## Supplementary Material

Traditional Knowledge of Bowhead Whale Migratory Patterns near Wainwright, Alaska

## Supplementary Material

Traditional Knowledge Regarding Walrus near Point Lay and Wainwright, Alaska

## Supplementary Material

Traditional Knowledge Regarding Walrus near Point Hope, Alaska

## Supplementary Material

Traditional Knowledge Regarding Walrus, Ringed Seals, and Bearded Seals near Barrow, Alaska

## Supplementary Material

ADF&G-ELI 2015

## Supplementary Material

ADF&G-SMK SBB 2015

## Supplementary Material

ADF&G-KVA 2016

## Supplementary Material

ADF&G-OTZ 2016

## Supplementary Material

ADF&G-SHH 2016

## Data accessibility

Data from our research are provided as electronic supplementary material, S1–S10, to this article. It consists of the interview summaries, reviewed and approved by participants for accuracy.
