# Supplementary material for: Effects of changing sea ice on marine mammals and subsistence hunters in northern Alaska from traditional knowledge interviews
Source: Biol Lett. 2016 Aug;12(8):20160198. doi: 10.1098/rsbl.2016.0198 (PMC5014017; doi:10.1098/rsbl.2016.0198)
Supplement: Traditional Knowledge of Bowhead Whale Migratory Patterns near Kaktovik and Barrow, Alaska [file rsbl20160198supp1.pdf]

Traditional Knowledge of Bowhead Whale Migratory Patterns near Kaktovik and Barrow, Alaska

Report to:

The Barrow and Kaktovik Whaling Captains Associations
and
The Alaska Eskimo Whaling Commission

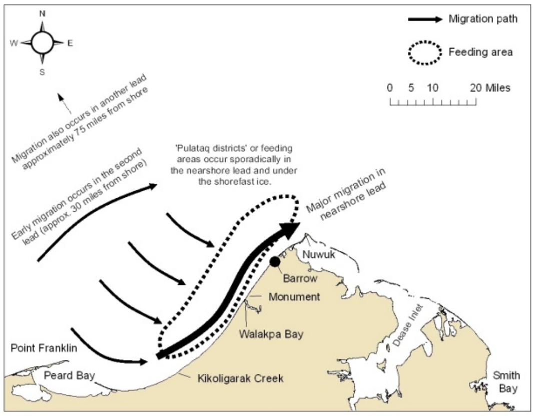

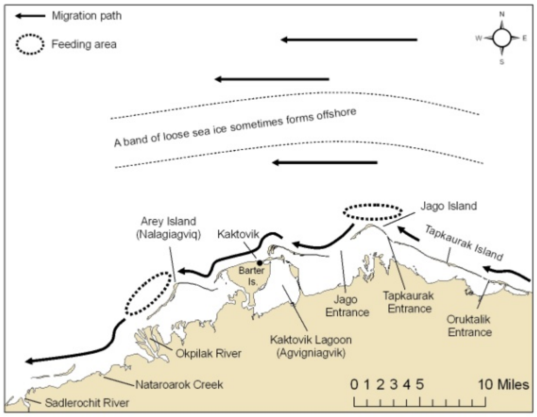

By:

Henry P. Huntington
Huntington Consultants
Eagle River, Alaska
hph@alaska.net
Ph: (907) 696-3564

And

Lori T. Quakenbush
Alaska Department of Fish and Game
Fairbanks, Alaska
lori.quakenbush@alaska.gov
Ph: (907) 459-7214

October 2009

This report should be cited as:
Huntington, H.P. and L.T. Quakenbush. 2009. Traditional knowledge of bowhead whale migratory patterns near Kaktovik and Barrow, Alaska. Final report to the Barrow and Kaktovik Whaling Captains Associations, the Alaska Eskimo Whaling Commission, ConocoPhillips, and the Minerals Management Service. 13pp.

Traditional Knowledge of Bowhead Whale Migratory Patterns near Kaktovik and Barrow, Alaska

Henry P. Huntington and Lori T. Quakenbush

Introduction

The general migratory patterns of bowhead whales in the Bering, Chukchi, and Beaufort Seas are well documented. The details of whale movements and activity throughout the migratory range are not as well known. Offshore industrial activity in the region is increasing, including seismic exploration, oil and gas development, and ship traffic. A better understanding of bowhead movements and activity is needed to help determine how to minimize the impacts of industrial activity on whales and those who hunt them. The Alaska Department of Fish and Game (ADF&G), in cooperation with the Alaska Eskimo Whaling Commission (AEWC), is conducting two related projects to learn and document more about bowhead whale movements and activities.

First, ADF&G is placing satellite transmitters on a small number of whales to learn more about the movements and behavior of individual whales over large portions of the migratory range. To date, 40 transmitters have been deployed; 31 in Alaska and 9 in Canada. Thirty-one were placed on whales near Barrow; five in spring and 26 in fall, and most were tagged by Barrow whalers. Efforts to place transmitters on whales near Kaktovik have not yet been successful due to weather conditions. In 2007, ADF&G and whalers from Kaktovik and Point Hope, Alaska, have worked with the Canadian communities of Aklavik and Tuktoyaktak to tag nine bowheads in August. George Tagarook and Eddie Arey from Kaktovik assisted with this tagging effort at Shingle Point. In 2008, ADF&G worked with the Canadian community of Tuktoyaktuk to tag one bowhead in August. George Tagarook from Kaktovik and Ray Koonuk from Point Hope assisted with this tagging effort near Atkinson Point. In Alaska, ADF&G plans to continue its efforts in Barrow and also to work with whaling captains on St. Lawrence Island to extend its work to the Bering Sea. The satellite transmitter work has been

funded by the Minerals Management Service (MMS) and tagging efforts in Canada are supported by the Department of Fisheries and Oceans Canada.

Second, at the request of the AEWC, ADF&G has interviewed whaling captains and crew members in Kaktovik and Barrow to document traditional knowledge of bowhead movements and behavior near those communities. This information will provide details about the patterns of large numbers of whales in more localized areas, complementing the information gathered by satellite telemetry about patterns of a few whales over larger areas. The traditional knowledge project uses the same approach that the Native Village of Savoonga used when documenting traditional knowledge about bowhead whales on St. Lawrence Island (Noongwook et al. 2007). This study was funded by a grant from ConocoPhillips. This report presents the results of the interviews.

Kaktovik

Bowhead whales have occasionally been seen in the Kaktovik area in July and early August, for example in Camden Bay or 8–10 miles offshore from Kaktovik. The main migration begins in late August, with whales moving westwards (Figure 1). The first whales in the migration are typically large ones that establish the route of the migration. Whalers do not hunt these whales, allowing the migratory pattern to be established in the expectation that later whales will follow regardless of whaling activity. This behavior is also recognized in migrating caribou and other animals. The migration continues through September and into October, but Kaktovik whalers stop whaling before the migration ends and so are not sure how late in the fall the whales continue to come by.

Some whales travel close to the shore, pausing to feed in the passes between barrier islands or just off of the islands where water flowing from the lagoons mixes with ocean water. In these areas, bowheads leave depressions in the sea floor that can be seen by whalers when the water is calm. Whales have been seen with mud on their stomachs when feeding in these areas. Other whales travel farther offshore, generally swimming steadily westward. “Traveling” whales can be distinguished from feeding whales because traveling whales surface to blow once and then continue, whereas feeding whales surface

many times and stay in the same area. When the leader of a group of feeding whales shows its flukes during a dive, it signals to the other whales that it is time to leave. Whales are not seen close to shore every year. That whales have long been found nearshore is indicated by Arey Island's Iñupiaq name, Nalagiagviq, which means "place to listen for whales."

In some years, there is a band of loose pack ice (it is possible to boat through without difficulty) a few miles wide between 5 and 15 miles offshore. Bowheads are known to migrate on both sides of the ice and also among the ice floes. When being hunted, whales may hide or seek refuge in the ice. In years with ice, bowheads are generally closer to shore than in years without ice. Sometimes whalers go as far as 20 miles offshore to find whales.

Kaktovik whalers have seen few patterns in whale size or other characteristics during the bowhead migration. Large whales may tend to come earlier (not counting the first few whales that set the migratory path), but whales of all sizes are seen throughout the migratory period. Whalers noted that they do not see the "super-big" whales that are sometimes taken at Barrow. They speculated that those whales may be farther offshore than the whalers go (i.e., more than 20 miles). Cows and calves start coming by in mid-September, later than the earliest whales to migrate past Kaktovik. Small whales may be more common close to shore, but large whales and even cow-calf pairs are seen close to shore, too. Calves may be separate from their mothers, making it difficult for whalers to tell if a small whale is a calf or not.

Since whaling resumed in Kaktovik in the early 1960s, whalers have noticed more whales and a decrease in sea ice during whaling season. Whalers used to be able to climb onto ice floes and use high ice as a lookout for whales. Other aspects of whale behavior, such as the timing of the migration or feeding behavior near shore, have not changed. There has been considerable change in the barrier islands, with both erosion and build-up seen. Some passes have become shallower. Until the 1950s, the lagoon now called Kaktovik Lagoon was known as Agvigniagvik, or "place to hunt whales," from an earlier period of

whaling in the region. Today, beluga whales occasionally enter this lagoon, but it is too shallow for bowheads.

Bowhead whales are known to be sensitive to noise. When a thermos was accidentally knocked over in one whaling boat, a nearby bowhead whale immediately dove and was not seen again. Kaktovik whalers are thus worried about offshore oil activity in their area, fearing that the noise may deflect bowhead whales away from shore.

In the fall of 2006, whalers noticed millions of jellyfish in the waters off Kaktovik. This has been seen in other years, too. A Bering wolfish was caught near Kaktovik in the summer of 2006, the first time one had been seen in the area. Overall, fishing success has declined in recent years. Dolly varden and cisco were the most common fish. Today, there are more salmon than formerly.

Kaktovik whalers generally do not begin whaling until early September, when the air temperature is cool enough to preserve the meat during butchering. They typically finish whaling in September, having reached their allocation of whales before the migration ends and before the weather deteriorates. Prior to and after whaling, Kaktovik residents are typically hunting on land or fishing in rivers or the nearshore, and thus have limited opportunities to see whales when they are not actively whaling.

Barrow

Bowhead whales have been seen near Barrow as early as February, but the main migration begins in mid-April. Offshore from Barrow, there are three lead systems in the spring sea ice. Moving outwards from shore, the first lead begins at the edge of the shorefast ice. Beyond this lead, there is pack ice out to another lead about 30 miles offshore. The third lead is about 75 miles offshore, but is narrow. There are also different current movements in the different leads with the farthest lead having the strongest current. Whalers who flew planes while guiding polar bear hunts in the 1960s have noticed these patterns. When the second lead is long, a few large bowhead whales have

been seen there in early April, but beluga whales are more common in that lead than are bowheads.

The main migration in the nearshore lead begins with a small number of mid-sized whales (“qairaliq”), followed by larger numbers of small whales in mid- to late April (Figure 2). The whales are plentiful during the three to four days the first wave lasts. The whalers let the first 50–100 whales of the first wave go past to establish the path. A second wave, consisting of mid-sized whales, typically arrives in early May after a gap of two or three days from the first wave. The second wave has many whales, and lasts about a week. After another period of fewer whales, the final wave of large whales, including cows and calves, arrives in mid-May and continues into June.

One whaling crew has noticed a recognizable whale appearing year after year, always on April 23, indicating that perhaps some individual whales follow their own annual patterns.

Whale behavior is the same during all three waves, although whales in the first wave may spend more time in the area, feeding or playing. While many bowheads migrate quickly through the Barrow area (perhaps aware of the presence of whalers), some stop and feed under the shorefast ice. Feeding whales may circle many times under the ice, returning to open water to breathe. This behavior is termed “pulataq” in Iñupiaq, and the whalers recognize “pulataq districts” along the edge of the shorefast ice. Whaling camps may be located at places where bowheads are expected to emerge from under the shorefast ice. Whales migrating quickly through the area tend to be farther from the edge of the shorefast ice.

A cow with a calf may leave the calf in bays in the shorefast ice while the cow travels ahead to scout conditions along the route. The cow will then retrieve the calf and continue the migration. Some females give birth in the Barrow area.

Barrow residents often hunt walrus and seals offshore, west of Barrow in July. During the interviews, respondents did not report seeing bowhead whales west of Barrow in July. During the review of a draft of this report by the AEWC, however, it was remarked that bowhead whales have been seen west of Barrow in July in recent years. The difference may reflect the intentional selection of older respondents, many of whom are less active now and who therefore may not have observed recent changes in distribution, timing or behavior. Occasionally bowhead whales are seen north of Barrow in summer. Many gray whales are seen in this season. Gray whales may enter Dease Inlet, as do belugas, but bowhead whales require deeper water.

In the 1940s, when one respondent was growing up at Cape Halkett, bowhead whales were not seen in that area.

Bowhead whales return to the area near Point Barrow in late August, though some large whales were seen 20–30 miles offshore in open water in early August one year. Generally, the large whales come first in the fall migration, followed by mid-sized whales, with small whales coming last (Figure 3). This pattern is less distinct in fall than is the three-wave pattern in spring. Bowhead whales may feed near the barrier islands east of Point Barrow. Whales are heavier in fall than in spring. In years with heavy pack ice in fall, whales will head southwest from Point Barrow. In years with light or no pack ice in fall, whales may stay near Point Barrow longer before heading west. The migration tends to occur later in years with little or no ice than in years with heavy ice, with whales in the area through late October. Small whales that stay close to shore may encounter gray whales southwest of Barrow.

Barrow whalers have noticed an increase in the number of whales over the past several decades. At the same time, changes in ice conditions and an increase in noise from snowmachine travel on the shorefast ice have led to noticeable changes in the spring migration pattern near Barrow. Fewer bowheads travel next to the edge of the shorefast ice, and fewer bowheads are seen southwest of Barrow. Whaling crews that used to set up camps near the Monument (approximately 12 miles southwest of Barrow) have had to

move farther north along the shorefast ice. This shift may be the result of thinner ice conditions and less multi-year ice, which is associated with feeding opportunities for bowhead whales, thus reducing the attraction of the shorefast ice southwest of Barrow. Bowhead whales are also arriving earlier in spring now than they did in the past.

The shorefast ice has become thinner in spring and more susceptible to breaking off and being blown away from shore. The shore-fast ice breaks apart earlier than it used to. In fall, there is more open water and the ice forms later. In November 1964, a 28-foot bowhead whale was landed at Barrow and hauled up onto sea ice for butchering. In more recent years, no shorefast ice has been present in November, at least not of sufficient thickness to bear the weight of a bowhead whale and allow butchering to take place on the ice.

When a test well was drilled offshore near Point Barrow, whales diverted their migration around the area, even though no drilling occurred during the migration. The noise from the idle drill ship was still sufficient to affect the whales. After the drilling ceased and the rig was removed, the whales reverted to normal behavior in the area within a couple of years.

Barrow whalers have seen whales much larger than those that have been landed. Some whalers believe that the very large whales would have tough meat and maktak, and so might not be worth hunting.

Methods

This study used the same basic methods to document traditional knowledge as those used by Noongwook et al. (2007), and described in detail there. (That paper also describes the ways that traditional knowledge is acquired among Yupik whalers. The description is generally applicable to Barrow and Kaktovik as well.) Specifically, we used the semi-directive interview (Huntington 1998). Unlike Noongwook et al., however, our interviews were with one or two persons at a time, rather than with larger groups. In the semi-directive interview, researchers initiate a discussion around various topics of

interest, but allow the person being interviewed to determine the order in which topics are discussed and to make connections between various topics that the researchers might not have anticipated. The interview is thus more fluid than would be a standardized questionnaire. The interviews were conducted in English, as all participants were comfortable in that language.

The research trip took place in early February 2007. In Kaktovik, we interviewed six whalers or whaling captains. They had an average of about 30 years of whaling experience, ranging from 18 to 45 years. In Barrow, we interviewed five whalers or whaling captains plus one locally resident scientist who had over 25 years of experience working with and learning from the whalers. The Barrow whalers and whaling captains had an average of over 50 years of whaling experience, ranging from 40 to 64 years. In both cases, the persons being interviewed were recommended by the head of the local whaling captains association or by chain referral (one participant recommending additional persons to interview). This report includes the information told to us by the whaling captains and whalers that participated in interviews in each village. As noted earlier, there may be additional information known by others that is not included here, particularly concerning recent changes. Because the environment continues to change, there may be merit in developing a mechanism for recording and reporting ongoing observations made by whalers and hunters.

The researchers included a marine mammalogist (LTQ) who is also the principal investigator of the satellite transmitter study, and a social scientist (HPH) with experience in traditional knowledge studies (and who also took part in the St. Lawrence Island study mentioned earlier). Having both forms of expertise helped in the conduct of the interviews and in asking appropriate follow-up questions. The interviews also allowed LTQ to share preliminary results of the satellite transmitter study, specifically the movements of two tagged whales. This information was in most cases shared at the end of the interview, the exceptions occurring when one respondent arrived as the previous interview was ending.

Following the trip, LTQ and HPH prepared a draft report, which was provided to the individuals who were interviewed and the presidents of the Barrow and Kaktovik whaling captains associations for corrections and comments. The corrections and comments were incorporated into a final report, which was approved by the AEWC.

Acknowledgments

We are grateful to ConocoPhillips for funding the traditional knowledge project. We are particularly grateful to the Alaska Eskimo Whaling Commission for its support and encouragement, and to the Barrow Whaling Captains Association and the Kaktovik Whaling Captains Association for helping set up interviews, plan the research trip, and review the draft report. Eugene Brower and Fenton Rexford were extremely helpful as our primary local contacts. John "Craig" George in Barrow helped identify participants and also agreed to be interviewed himself. In Kaktovik, we interviewed Charles M. Brower, Eddie Rexford, and four others who preferred to remain anonymous. In Barrow, we interviewed Ben Itta, David U. Leavitt, Warren Matumeak, Johnnie Brower, John "Craig" George, and one individual who preferred to remain anonymous. Although Harry Brower, Jr. was not interviewed, he assisted in developing the project and he reviewed the draft report and provided valuable comments. John Citta prepared the maps used during interviews and the figures in this report. Minerals Management Service is funding the satellite telemetry project that prompted the traditional knowledge interviews.

References

Huntington, H.P. 1998. Observations on the utility of the semi-directive interview for documenting traditional ecological knowledge. Arctic 51(3):237-242.

Noongwook, G., the Native Village of Gambell, the Native Village of Savoonga, H.P. Huntington, and J.C. George. 2007. Traditional knowledge of the bowhead whale (*Balaena mysticetus*) around St. Lawrence Island, Alaska. Arctic 60(1):47–54.

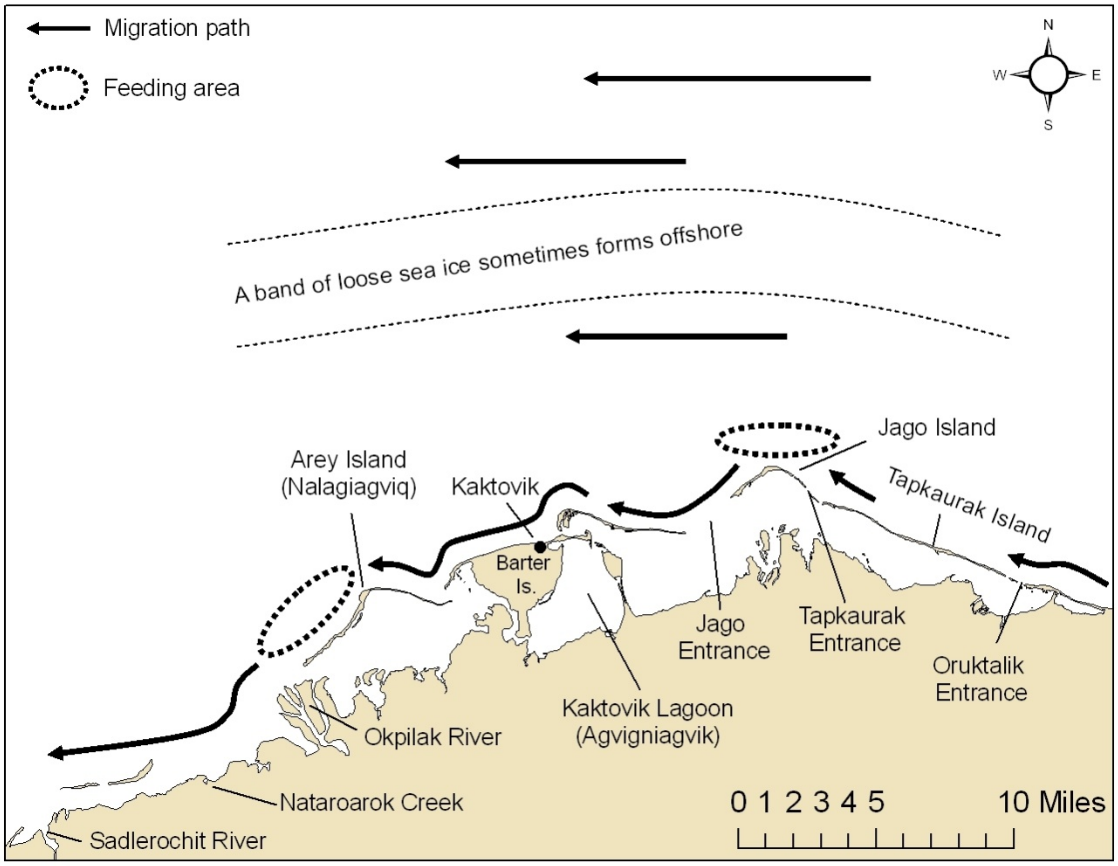

Figure 1. Movements and behavior of bowhead whales near Kaktovik, Alaska in August–September. Some whales travel close to shore, feeding in the passes between barrier islands and outside of Jago and Arey islands, however they may feed also feed at other places along the coast in this area. Whales also pass farther offshore and whalers may go as far as 20 miles from shore to find whales. Sometimes there is a band of loose ice 5–15 miles offshore. Whales travel to the north and south of this ice and through it. In years with more ice whales tend to travel closer to shore.

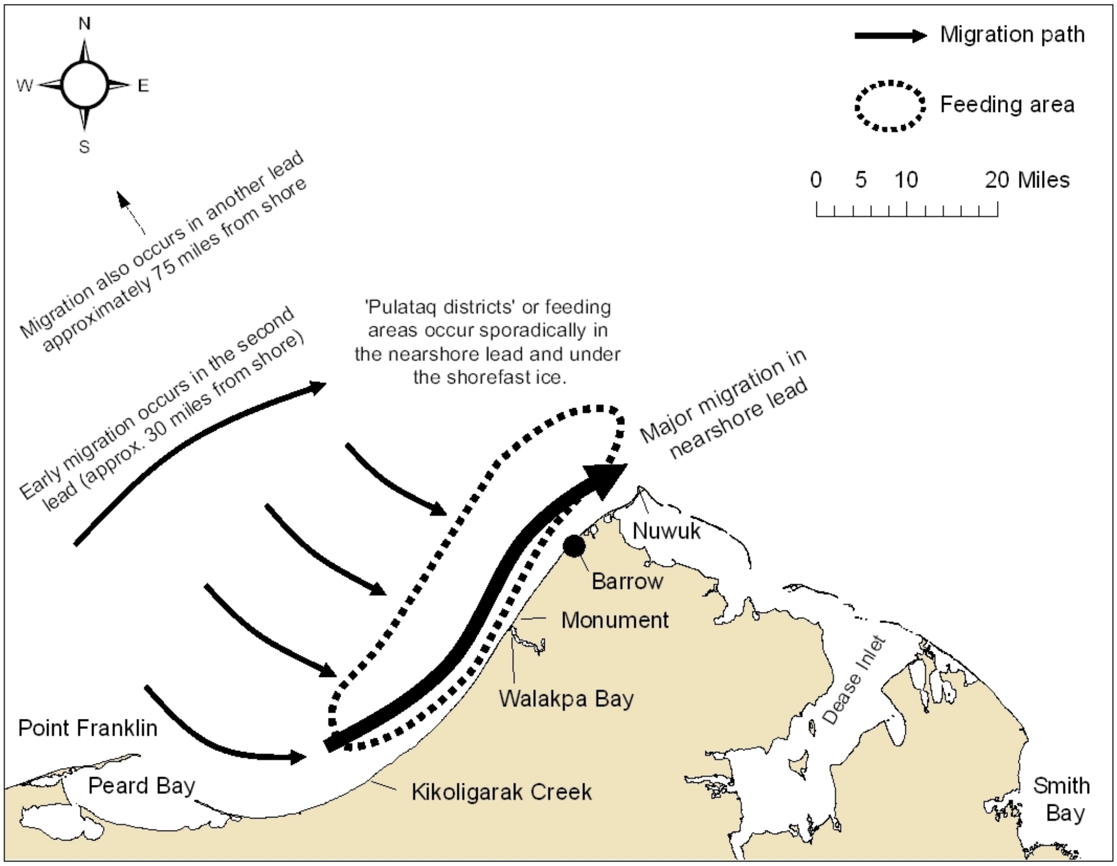

Figure 2. Movements of bowhead whales in spring near Barrow, Alaska. There are three lead systems in the spring sea ice. The closest one begins at the edge of the shorefast ice and is where the main migration occurs. The second lead occurs about 30 miles out and a third occurs about 75 miles out. Some whales move through quickly and some circle under the ice to feed. These feeding areas are called “pulataq districts”.

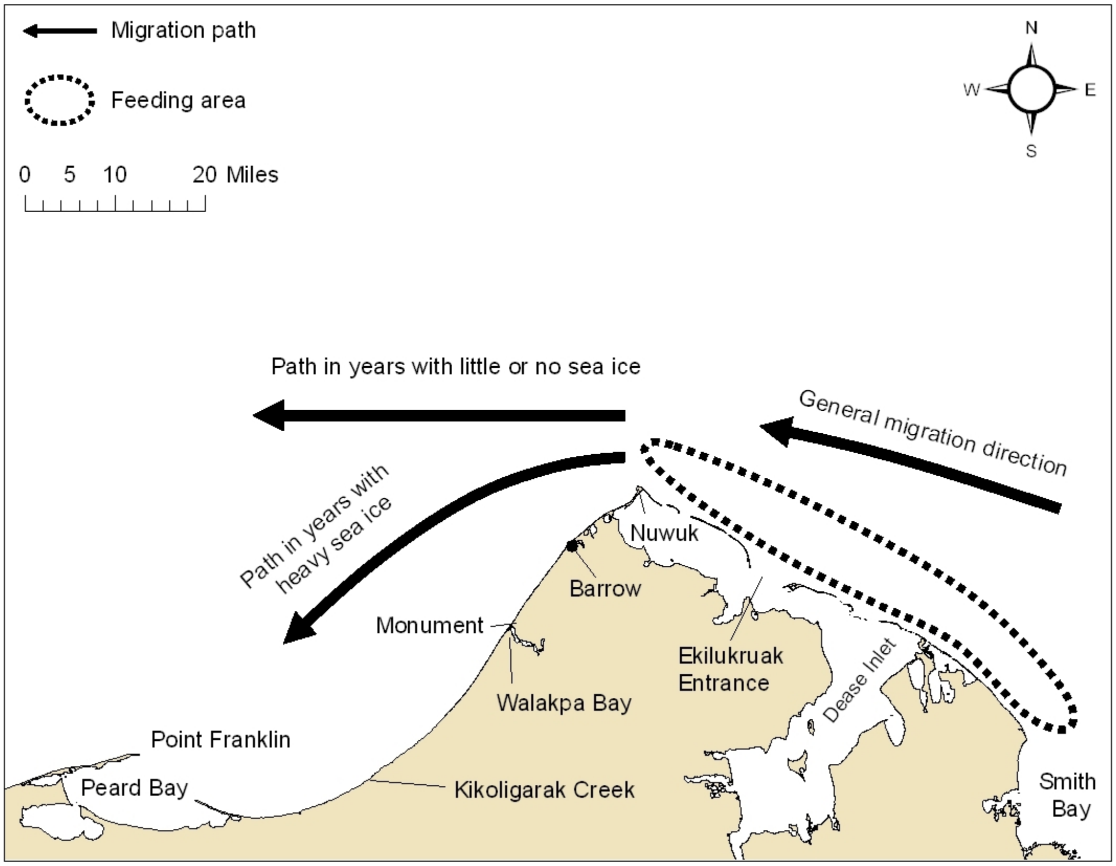

Figure 3. Movements and behavior of bowhead whales in fall near Barrow, Alaska. Bowhead whales may feed near the barrier islands between Nuwuk and Dease Inlet. In years with little or no ice, whales may stay longer near Barrow and head more west when they leave. In years with heavy ice, the whales head more southwest and they leave earlier.
