# Supplementary material for: Effects of changing sea ice on marine mammals and subsistence hunters in northern Alaska from traditional knowledge interviews
Source: Biol Lett. 2016 Aug;12(8):20160198. doi: 10.1098/rsbl.2016.0198 (PMC5014017; doi:10.1098/rsbl.2016.0198)
Supplement: Traditional Knowledge of Bowhead Whale Migratory Patterns near Wainwright, Alaska [file rsbl20160198supp2.pdf]

Traditional Knowledge of Bowhead Whale Migratory Patterns near Wainwright, Alaska

Report to:

The Alaska Eskimo Whaling Commission

and

The Wainwright Whaling Captains Association

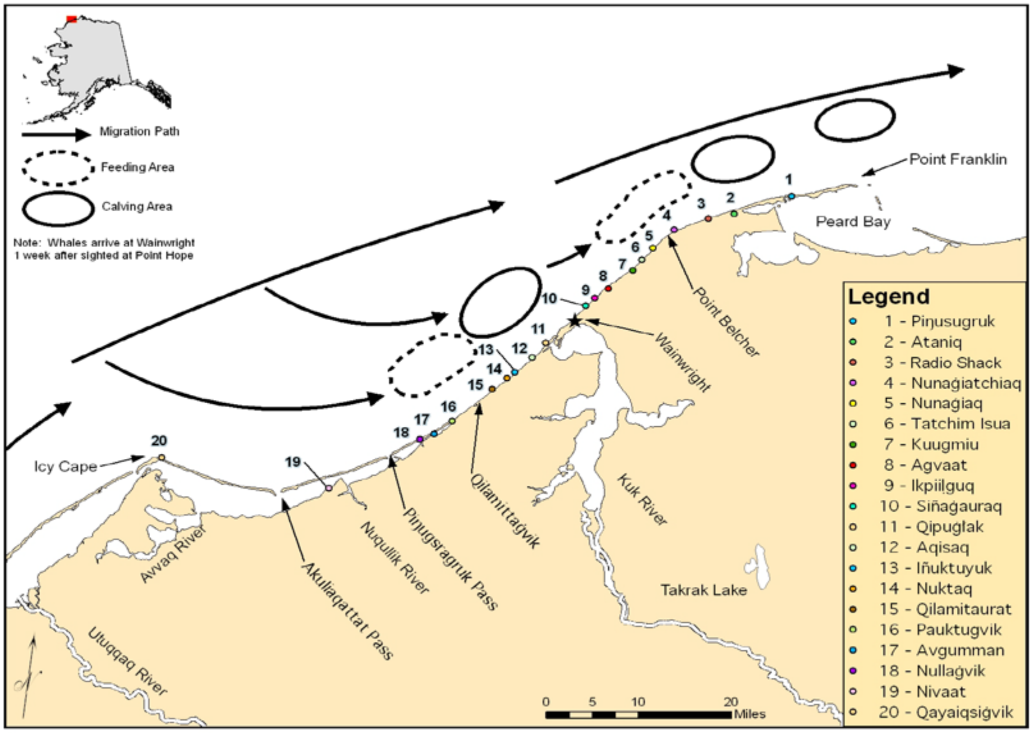

By

Henry P. Huntington
Huntington Consulting
Eagle River, Alaska
hph@alaska.net
and
Lori T. Quakenbush
lori.quakenbush@alaska.gov

October 2009

This report should be cited as:
Huntington, H.P. and L.T. Quakenbush. 2009. Traditional knowledge of bowhead whale migratory patterns near Wainwright, Alaska. Final report to the Wainwright Whaling Captains Associations, the Alaska Eskimo Whaling Commission, ConocoPhillips, Minerals Management Service, and the Coastal Marine Institute. 9pp.

Introduction

The general migratory patterns of bowhead whales in the Bering, Chukchi, and Beaufort seas are well documented. The details of whale movements and activity throughout the migratory range are not as well known. Offshore industrial activity in the region is increasing, including seismic exploration, oil and gas development, and ship traffic. A better understanding of bowhead movements and activity is needed to help determine how to minimize the impacts of industrial activity on whales and those who hunt them. The Alaska Department of Fish and Game (ADF&G), in cooperation with the Alaska Eskimo Whaling Commission (AEWC), is conducting two related projects to learn and document more about bowhead whale movements and activities.

First, satellite transmitters are being placed on a small number of whales to learn more about the movements and behavior of individual whales over large portions of the migratory range. To date, 40 transmitters have been deployed; 31 in Alaska and 9 in Canada. Thirty-one were placed on whales near Barrow, five in spring and 26 in fall, and most were tagged by Barrow whalers. Efforts to place transmitters on whales near Kaktovik have not yet been successful due to weather conditions. Since 2007, ADF&G and whalers from Kaktovik and Point Hope, Alaska, have worked with the Canadian communities of Aklavik and Tuktoyaktuk to tag nine bowheads in August. In Alaska, ADF&G plans to continue its efforts in Barrow during spring and fall and also to work with whaling captains on St. Lawrence Island to increase knowledge of bowhead movements in the Bering Sea. The satellite transmitter work has been funded by the Minerals Management Service (MMS) and tagging efforts in Canada are supported by the Department of Fisheries and Oceans Canada.

Second, at the request of the AEWC, ADF&G has interviewed whaling captains and crew members in Kaktovik, Barrow, and Wainwright to document traditional knowledge of bowhead movements and behavior near those communities. This information will provide details about the patterns of large numbers of whales in more localized areas, complementing the information gathered by satellite telemetry about patterns of a few whales over larger areas. The traditional

knowledge project uses the same approach that the Native Village of Savoonga used when documenting traditional knowledge about bowhead whales on St. Lawrence Island (Noongwook et al. 2007). Traditional knowledge research in Kaktovik and Barrow was funded by a grant from ConocoPhillips (Huntington and Quakenbush 2009) and this funding was used as match by the Coastal Marine Institute (CMI) for research in Wainwright. This report presents the results of the interviews in Wainwright.

Wainwright

The movements of bowhead whales near Wainwright are determined primarily by ice conditions. Leads in the local area affect local distribution, whereas the condition of leads to the south influences the timing of the migration as a whole. The prevailing east-northeast winds tend to open the leads near Wainwright, with currents playing a role, too. West winds tend to close the lead, making whaling impossible. When the lead is closed, the whales travel farther from the shorefast ice. Currents are stronger by Point Belcher, and there is a strong current near the Kuk River mouth by Wainwright in late May and early June.

Wainwright whalers hear from St. Lawrence Island and from Point Hope that bowheads are migrating. They expect bowheads to reach the Wainwright area about a week after they reach Point Hope, depending on ice conditions in between. Before whaling, Wainwright whalers wait for the leads to be open wide, to help reduce the struck-and-lost rate. The pattern of leads varies from year to year in the Wainwright area. In some years, the lead has remained open all spring, whereas in other years there has been hardly any open water during whaling season. When nearshore leads do not open, whalers may have to travel farther offshore, either across young ice or on un-grounded ice susceptible to breaking off, both of which are dangerous.

In spring, whalers recognize three waves (sometimes called schools, runs, or pulses) of whales. The first wave is primarily small, young whales, and occurs when the leads first open. Formerly, this would occur in late April, but in recent years has been taking place earlier so that bowhead whales now appear in the area in early April and at times even in March. The whales in the first wave migrate past Wainwright through open leads or ponds of open water. The second wave of whales, comprised of mid-sized whales, also requires open leads or ponds. For both waves, if the

lead closes or if leads are not available to the south, the whales may delay their migration to await more favorable conditions. The whales may congregate in open pools while waiting for the leads to open up.

The third wave of whales, which includes the largest whales and also most of the mother-and-calf pairs, takes place in the second half of May and early June. These whales are capable of pushing up through young ice (up to approximately 18” or 45 cm thick) to create breathing holes. Subsequent whales will use the same breathing holes, which can be dangerous to people on the ice if the holes are covered with snow and thus invisible from above. Elders used to tell the young boys at whaling camps to keep quiet so that blows could be heard even when the lead was closed and so that the whales would not be disturbed while setting the migratory path. Later whales follow the pattern set by the first animals. Bowheads can also find cracks in thicker ice through which they can breathe. They are thus able to migrate even when the main lead system is closed. Whales in the third run may also be found in cracks and openings far out in the pack ice.

Wainwright whalers seek open leads closest to shore. The whalers will have been on the ice throughout the winter, watching where the ice breaks off and assessing where it is likely to do so in spring, which helps them plan. In most years, whaling starts near Point Belcher to the north of the village (Fig. 1), where the currents and ice conditions tend to create leads closer to shore. The ice is typically rough in this area. A key concern is finding a suitable location for hauling a whale onto the ice for butchering. The ice needs to be thick enough to support a whale, with a large enough flat area for the whale and the cutting-up activities. The whalers begin by seeking such a spot, often traveling the lead edge by boat and then cutting a trail to the suitable location. (Searching on the shorefast ice would be much more difficult due to the difficulty of traveling over rough ice.) In recent years, the ice has been thinner, making it harder to find a good spot.

As the season progresses, leads may open up closer to Wainwright, and the whalers may move to the southwest from Point Belcher, seeking other locations to set up whaling camps. In some years, the whalers may go as far south as Icy Cape, though that is not as common. (One year, migrating ducks at Icy Cape were so numerous that the whalers’ camps and gear were covered in duck droppings, reducing the appeal of returning to that particular location.) The whales often

follow the ice edge, but may also travel directly from the Icy Cape area to the Point Belcher area, staying farther offshore as they pass the village.

During the spring migration, whales have been observed calving, mating, and feeding. Calving has been seen in a few locations (Fig. 1). Calves are occasionally seen in late April, but more typically in late May and June. Calves are small and gray, rather than the black of an older whale. Elders emphasized the need to look carefully when whaling to make sure that an adult whale was not accompanied by a calf. Whales with calves should not be taken. Mating behavior consists of several males and one female. The female and one male will stay in one location, rolling slowly over and over so that each has the opportunity to breathe without disengaging. The other males will slowly circle the mating pair. Whalers once asked elders about the advisability of hunting a mating pair. The elders discouraged this, saying that first the whales were making more whales, and second that they might become violent if disturbed at this time and thus would be hazardous to the whalers. For these reasons, mating whales are left alone.

Feeding is often seen along the edge of the shorefast ice (Fig. 1). Whales will swim parallel to the edge with their mouths open. They may have trouble closing their mouths, sometimes using the ice by pushing their lower jaw against the ice to help shut their mouths. Whales are also known to swim under the shorefast ice to feed, circling under a particular area or traversing the area by breathing through cracks in the shorefast ice or pushing up through young ice to create breathing holes. Whalers have seen and heard whales surfacing between their camps at the ice edge and land, as the whales migrate under the shorefast ice. In one case, the whalers had cut a hole in the ice behind their camp, and found a whale breathing in that hole. In another case, where whales were repeatedly circling under the shorefast ice, the whalers approached and the whales quickly moved away from the area. Whales also feed under young ice, where food can often be found.

Bowhead whales have been seen occasionally after the shorefast ice has broken up and also in fall near Wainwright. After the whaling season, hunters often seek bearded seals in the pack ice. At these times they may see bowhead whales still migrating. One such whale was seen near Point Franklin in June of 2007. Many large whales were seen in July 2007 while hunters were

out after bearded seals. These are believed to be part of the third run, the large whales that come last. Whales have been seen later in July on a few occasions, near Wainwright and Icy Cape. Three very large bowheads were seen by the Kuk River mouth one July about 35 years ago. In October, whales have been seen a few times near Wainwright, but they do not generally follow the coast southwards from Barrow.

Whalers have seen other species of whales in the region. Identifying which species is difficult, but characteristics such as a small dorsal fin make it clear that they are not bowhead or gray whales. Fin and minke whales are likely candidates, and there is a possibility that sperm and blue whales have been seen in the area, too. The sperm whale was seen during bearded seal hunting about 30 years ago, recognized by its blunt head (whalers being familiar with Moby Dick). Beluga whales are common during the spring migration, typically migrating ahead of one or more bowheads. In summer, belugas congregate near Icy Cape. In some years, they come close to Wainwright and are hunted, whereas in other years they do not come past Icy Cape along the coast.

In recent years, the ice has been changing. The ice used to start forming in October, but now may not form until December or even until after Christmas. The resulting thinner ice can be blown offshore more easily during winter storms, further reducing the time it has to thicken and become anchored to provide safe locations for whaling camps. Break-up of the shorefast ice used to occur in late June and July, but now the ice may start to rot in May (both from sun on top and from currents underneath), making travel on the ice dangerous and limiting the season that the whalers can be out whaling. The lack of multi-year ice and other large, thick floes has allowed the whales to begin their migration earlier in the spring.

Wainwright whalers are very concerned about offshore oil and gas activities in the Chukchi Sea. In 1968, there was seismic testing offshore during the spring migration. The whalers saw no whales, not even a blow, that spring. Barrow provided whale meat and maktak to Wainwright for Thanksgiving and Christmas. The whalers were supposed to have been compensated for the loss of whaling that year, but never were. For planned activities in the Chukchi, the whalers believe stringent conditions should be imposed. They also have seen impacts from the activity near

Prudhoe Bay, recognizing that Barrow and Nuiqsut whalers report that the whales now migrate farther offshore in fall than before. In Wainwright, this means that the whales will not travel southwest near the Chukchi coast, but will stay offshore to the north as they migrate across to the Russian coast.

Methods

This study used the same basic methods to document traditional knowledge as those used by Noongwook et al. (2007), and described in detail there. (That paper also describes the ways that traditional knowledge is acquired among Yupik whalers. The description is generally applicable to Iñupiat whalers of Wainwright as well.) Specifically, we used the semi-directive interview (Huntington 1998). As was the case in Noongwook et al. (2007), we conducted a single group interview with seven whaling captains, who were identified and invited to the interview by the head of the Wainwright Whaling Captains Association. The captains had an average of 35 years of whaling experience ranging from 15 to 45. The research took place in Wainwright in late February 2008.

In the semi-directive interview, researchers initiate a discussion around various topics of interest, but allow the person(s) being interviewed to determine the order in which topics are discussed and to make connections between various topics that the researchers might not have anticipated. The interview is thus more fluid than would be a standardized questionnaire. The interviews were conducted in English, as all participants were comfortable in that language, though the participants occasionally discussed a particular point in Iñupiaq before providing us with a summary in English.

The researchers included a marine mammalogist (LTQ) who is also the principal investigator of the satellite transmitter study, and a social scientist (HPH) with experience in traditional knowledge studies (and who also took part in the St. Lawrence Island study mentioned earlier). Having both forms of expertise helped in the conduct of the interviews and in asking appropriate follow-up questions. The interview also allowed LTQ to share preliminary results of the satellite transmitter study, specifically the movements of two tagged whales, at the end of the interview.

Following the trip, LTQ and HPH prepared a draft report, which was made available to the individuals who were interviewed for their review and comments. Comments and changes, if any, were incorporated into the final report.

Acknowledgments

We are particularly grateful to the Alaska Eskimo Whaling Commission for its support and encouragement, and to the Wainwright Whaling Captains Association for helping set up the interviews, plan the research trip, and review the draft report. Jack Panik was extremely helpful as our primary local contact. We appreciate the participation of Terry Tagarook and the other whaling captains, who chose to remain anonymous, to share their knowledge of bowhead whale behavior and whaling near Wainwright for this project. We thank the Coastal Marine Institute for funding the interviews, with funds originally from the Minerals Management Service and the matching funds by ConocoPhillips. We thank Caryn Rea for her role in providing the funds from ConocoPhillips. John Citta prepared the maps used during interviews and Justin Crawford made the figure in this report. Minerals Management Service is funding the satellite telemetry project that prompted the traditional knowledge interviews.

References

Huntington, H.P. 1998. Observations on the utility of the semi-directive interview for documenting traditional ecological knowledge. Arctic 51(3):237–242.

Huntington, H.P., and L.T. Quakenbush. 2009. Traditional knowledge of bowhead whale migratory patterns near Kaktovik and Barrow, Alaska. Report to the Barrow and Kaktovik Whaling Captains Associations and the Alaska Eskimo Whaling Commission.

Noongwook, G., the Native Village of Gambell, the Native Village of Savoonga, H.P. Huntington, and J.C. George. 2007. Traditional knowledge of the bowhead whale (*Balaena mysticetus*) around St. Lawrence Island, Alaska. Arctic 60(1):47–54.

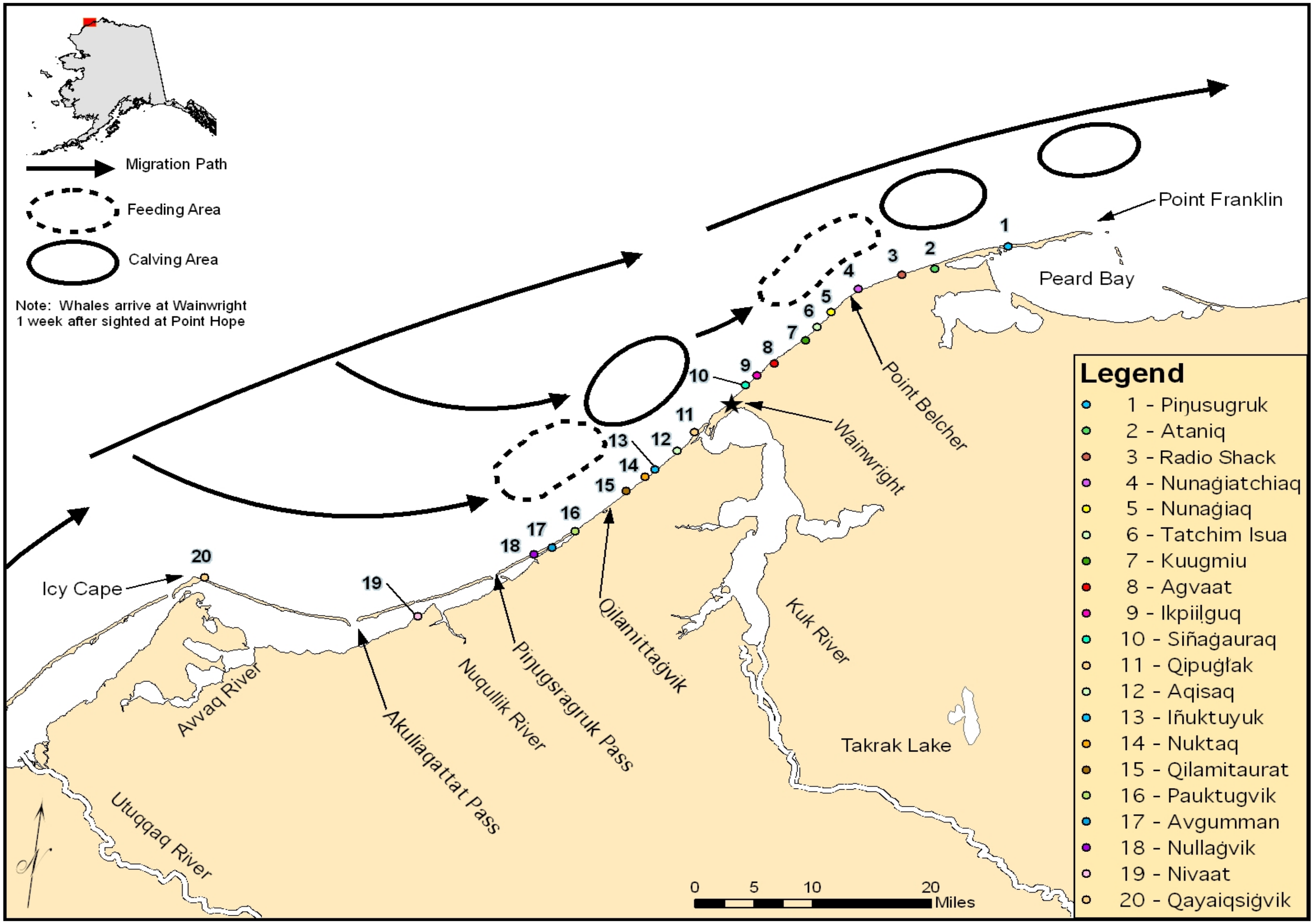

Figure 1. Movements and behavior of bowhead whales in spring near Wainwright, Alaska relative to local landmarks and other coastal features. Whales have been observed calving, mating, and feeding in the nearshore lead near Wainwright. Calves are occasionally seen in late April, but more typically in late May and June.
