# Supplementary material for: Effects of changing sea ice on marine mammals and subsistence hunters in northern Alaska from traditional knowledge interviews
Source: Biol Lett. 2016 Aug;12(8):20160198. doi: 10.1098/rsbl.2016.0198 (PMC5014017; doi:10.1098/rsbl.2016.0198)
Supplement: Traditional Knowledge Regarding Walrus near Point Lay and Wainwright, Alaska [file rsbl20160198supp3.pdf]

# Traditional Knowledge Regarding Walrus near Point Lay and Wainwright, Alaska

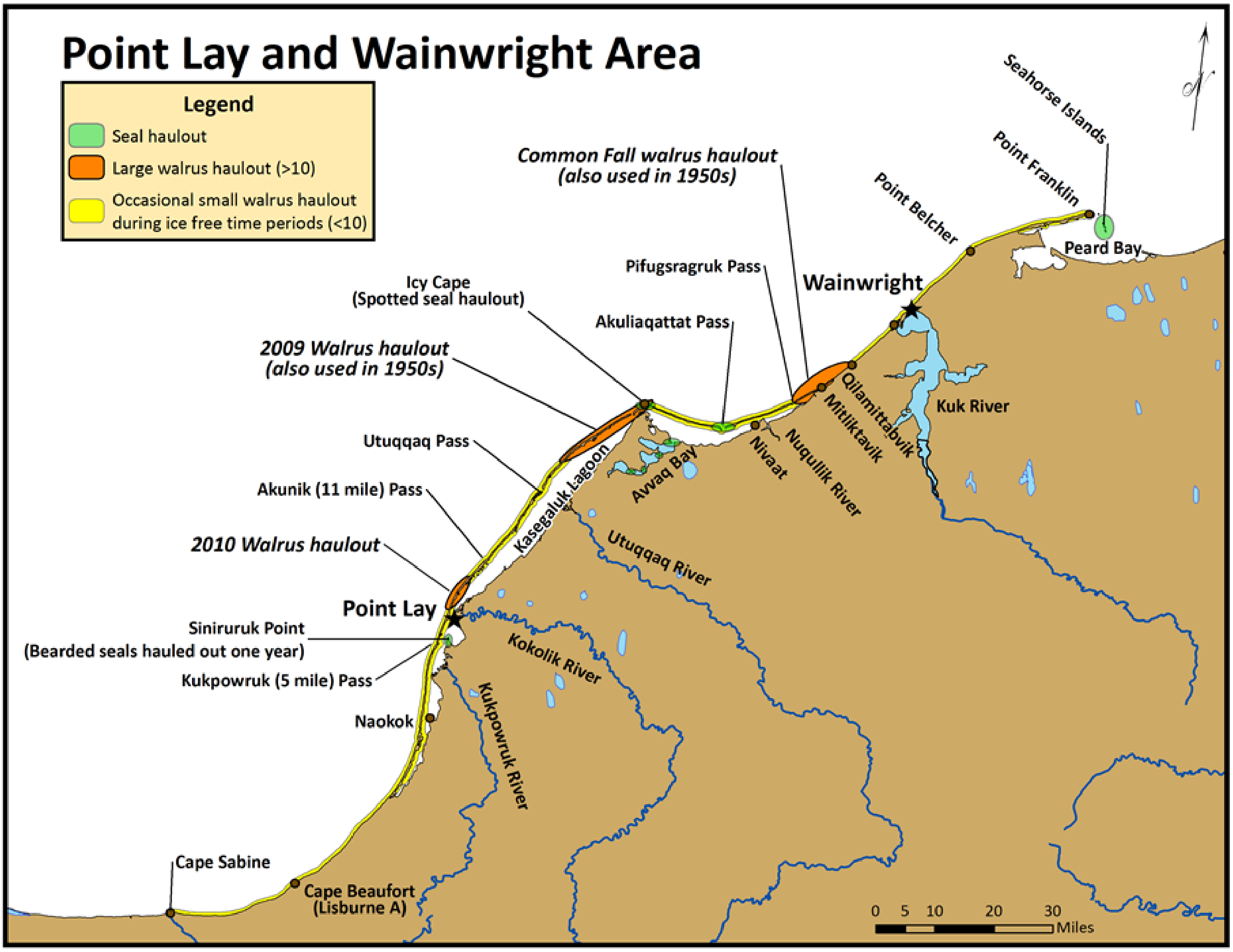

# Traditional Knowledge Regarding Walrus near Point Lay and Wainwright, Alaska

By:

Henry P. Huntington
Huntington Consulting
Eagle River, Alaska
hph@alaska.net
Ph: (907) 696-3564

Mark Nelson & Lori T. Quakenbush
Alaska Department of Fish and Game
Fairbanks, Alaska
mark.nelson@alaska.gov, lori.quakenbush@alaska.gov
Ph: (907) 459-7374, (907) 459-7214

Final Report
Approved December 2012

Final report should be cited as:
Huntington, H.P., M. Nelson, and L.T. Quakenbush. 2012. Traditional knowledge regarding walrus near Point Lay and Wainwright, Alaska. Final report to the Eskimo Walrus Commission and the Bureau of Ocean Energy Management for contract #M09PC00027. 11pp.

## Introduction

Walrus are an important species for subsistence harvests by Iñupiat hunters in northern Alaska. They are also an iconic Arctic animal, and at risk from climate change. Increasing industrial activity in the Chukchi Sea is an additional potential stressor to walrus. The study of walrus distribution, behavior, and movements is an important contribution to monitoring the effects of a changing environment and the potential effects from industrial activity. Placing satellite transmitters on walrus provides detailed information about the movements and some behaviors of individual animals. Documenting traditional knowledge about walrus, through interviews with residents of coastal communities, provides complementary contemporaneous and historical information about general patterns in walrus distribution, movement, and behavior.

This report summarizes information gathered from interviews with hunters and other knowledgeable residents in Point Lay and Wainwright, Alaska, in March 2011. This traditional knowledge project used the same approach that the Native Village of Savoonga used when documenting traditional knowledge about bowhead whales on St. Lawrence Island (Noongwook et al. 2007).

## Methods

We used the semi-directive interview method, in which the interviewers raise a number of topics with the person being interviewed, but do not rely solely on a formal list of questions (Huntington 1998). Instead, the interview is closer to a discussion or conversation, proceeding in directions determined by the person being interviewed, reflecting his/her knowledge, the associations s/he makes between walrus and other parts of the environment, and so on. The interviewers use their list of topics to raise additional points for discussion, but do not curtail discussion of additional topics introduced by the person being interviewed. In Point Lay, the interviews were conducted individually. In Wainwright, one group interview was conducted.

The topics identified by the research team in advance of the interviewers were:

Walrus distribution and abundance near communities
Distribution and sightings of walrus throughout the year
Walrus haul-out patterns on land
Sensitivity of walrus to various types of disturbance
Haul-out patterns of other pinnipeds
Changes over time for all of the topics

The results are presented under different headings, reflecting the actual information collected and the fact that some of the subjects blend together, especially changes seen over time in regard to all of the topics. The interviewers were Henry Huntington and Mark Nelson. Lori Quakenbush is the project leader.

## Point Lay

In Point Lay, we interviewed five people individually: Leo P. Ferreira III, Willard Neakok, James Tazruk, and Bill Tracey Sr., and one person who chose to remain anonymous. The interviews were conducted on 15 and 16 March 2011, at various locations in Point Lay.

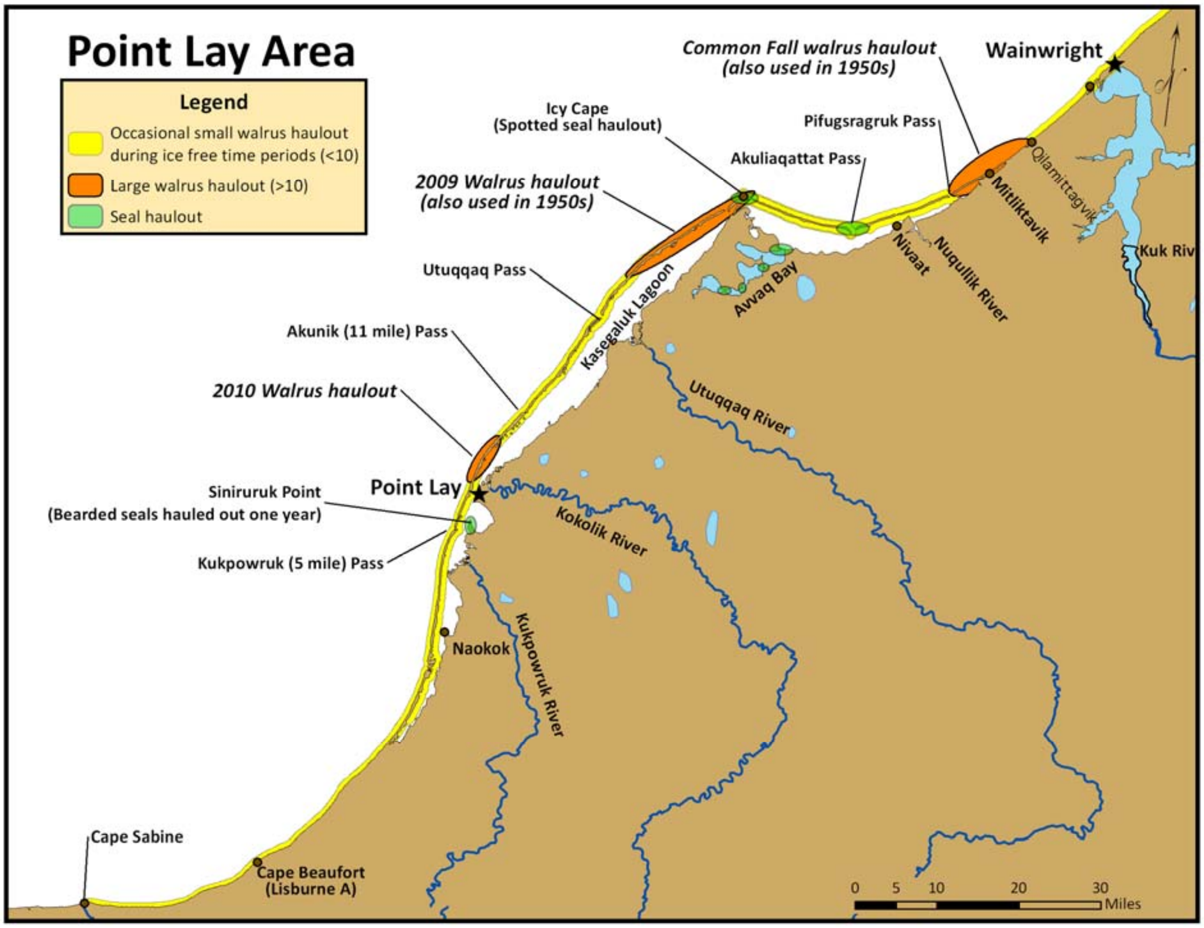


Figure 1. Seal and walrus haulout sites as recorded during traditional knowledge interviews in Point Lay.

### *Walrus*

Walrus are seen in the Point Lay area primarily in spring and fall. In May and early June, they are seen on sea ice, particularly during break-up when hunters are out hunting for bearded seals (*ugruk*) and walrus from boats. The walrus generally travel north with the ice, in groups of a few animals to a few dozen. Occasionally, walrus are seen feeding in the passes to Kasegaluk Lagoon, in the open water there at the start of break-up. A few stragglers are occasionally seen swimming north after the ice has gone. Lone walrus are not often seen, and may be sick or tired. One hunter reported seeing a walrus in an open lead one March near Wainwright, on a day when he also saw a huge bowhead whale in the lead. There was a large breathing hole nearby and it was speculated that the walrus spent part of the winter using this breathing hole. This was a one-time occurrence.

In late August and September, walrus are seen hauled out on land. Typically, a few walrus are seen at various locations along the barrier islands that form Kasegaluk Lagoon. They may also be seen farther south, at Cape Sabine and Cape Beaufort. Larger numbers are sometimes seen east of Icy Cape, at the entrances to Avvaq Bay, and between Utuqqaq Pass and Icy Cape. Elders who are no longer alive reported seeing large numbers of walrus hauled out between Point Lay and Wainwright in the 1950s, too. A few walrus may be seen in Kasegaluk Lagoon, but this is not common, and those walrus may not be healthy.

In 2009 and 2010, however, much larger numbers of walrus hauled out on shore. In 2009, the main haul-out was near Icy Cape. In 2010, some 20,000 walrus hauled out on the barrier islands a mile or two north of Point Lay, and walrus were seen in the water all the way to Icy Cape. Although not the first time large haul-outs had been seen, this was the first time one had occurred so close to the community. Most of the walrus hauled out appeared to be juveniles and females. Few large bulls were seen. Large haulouts like this had been seen only occasionally previous to 2009, typically north of Point Lay near Icy Cape.

Walrus on land are sensitive to smells, noises, and movements of other animals. Persons approaching from upwind who are wearing cologne, have been smoking, or have been drinking strong coffee will cause the walrus to take notice and, if the approach is close enough, to flee. Outboard motors and gunshots also cause walrus to flee. Boaters have to be especially careful in the vicinity of haul-outs, since many walrus are in the water and may attack a boat if provoked. Walrus may react more to persons whispering than to those talking in conversational tones, perhaps because they distinguish stalking and hunting behavior from non-threatening curiosity. The sight of a person approaching on foot, or the sight of a polar bear or brown bear, will also cause walrus to flee. Hunters approaching carefully, however, can get within a few yards without causing the walrus to react.

In 2010, the walrus hauled out near Point Lay could be heard from the village, especially in the morning and evening. The walrus were constantly going to and from the haul-out, but may have been even more active in morning and evening. Community residents avoided the haul-out, adjusted boating routes and behavior to avoid disturbing the walrus, and requested airplanes to stay at least 1500 feet above the haul-out. Planes landing at the Point Lay runway approached and took off from the inland end of the runway rather than over the lagoon and the haul-out. The community also regulated the presence of visitors, turning down most media requests to visit the haul-out.

As a result, relatively few walrus were killed on the haul-out site. In previous years, a few walrus carcasses could usually be found between Cape Sabine and Icy Cape. In 2009 and to a lesser extent in 2010, more walrus carcasses have been found at the sites of the large haul-outs, most likely from stampedes caused by disturbance of one kind or another. The higher mortality in 2009 was likely due to greater disturbance, as there were more reports of brown bears and polar bears in the area that year. Most of these carcasses are younger walrus. Polar bears, wolverines, and gulls scavenge the carcasses.

Apart from the recent changes in haul-out locations and the number of animals hauling out, Point Lay residents have seen few if any changes in walrus abundance, distribution, and behavior.

Very rarely, walrus have three or four tusks. One hunter reported seeing an albino walrus, with white skin and red eyes.

Some walrus are seen with dark skin, the color of bearded seals. These are sometimes called the "kings of the walrus." Large bulls may overwinter in the Chukchi Sea, too, maintaining breathing holes in the ice.

Walrus are not seen during the time when the barges are delivering supplies to Point Lay. Whether the walrus have not yet returned from farther north and offshore, or whether the barges keep the walrus away, is not clear. The barge traffic is concentrated in a week or two in late summer.

Walrus body condition appears to be good, with no change over time.

## *Seals*

Bearded seals and spotted seals are often seen hauled out on the barrier islands, particularly next to the passes into Kasegaluk Lagoon. Spotted seals are especially common on small islands on the north side of Utuqqaq Pass and also at the entrance to Avvaq Bay. A few dozen animals are seen at the former location, and several hundred may be seen at the latter. Bearded seals are seen at the entrances to other passes, and sometimes both bearded and spotted seals are seen hauled out together. On one occasion, a large number of bearded seals were seen hauled out on the mainland at Siniruruk Point just south of Point Lay, inside the Lagoon. More seals haul out north of Point Lay than to the south, perhaps because of more abundant fish.

Ringed seals are rarely seen hauled out on land. When hunters have seen them, the ringed seals are alone, and hunters suspect they may be ill or exhausted. Unlike bearded and spotted seals, ringed seals on land do not flee the approach of hunters.

Seals feed at the passes throughout the summer, and young bearded seals may be found up rivers chasing fish.

On haulouts, seals are sensitive to noise, such as outboard engines or gunshots.

Since the walrus have been hauling out near Point Lay, fewer seals have been seen because seals avoid walrus.

In late spring, ringed and bearded seals can be seen on the ice, basking in the sun, as far as the eye can see.

## *Other Information*

There is a north-running current about 10–15 miles offshore from Point Lay. In this area, seals and birds are often found, feeding on the plankton and fishes carried by the current.

In 2010, some 200 porpoises (harbor porpoises, *Phocoena phocoena*) were seen swimming along the barrier islands near the walrus haul-out. There appeared to be no interaction between the walrus and the porpoises. Beluga whales, on the other hand, behave differently when there are walrus around, appearing more nervous and harder to herd and hunt.

## Wainwright

In Wainwright, we held one group interview with 13 people: Enoch Oktollik, Andrew Ekak, Leslie Segevan, Artie Kittick, Jack Oktollik, Edwin Tazruk, Edward Kagak, and six interviewees who chose to remain anonymous. These men ranged in age from about 30 to 70+, providing a wide range of experience over time. The group interview format also allowed a great deal of interaction among the participants, including sharing of information across generations.

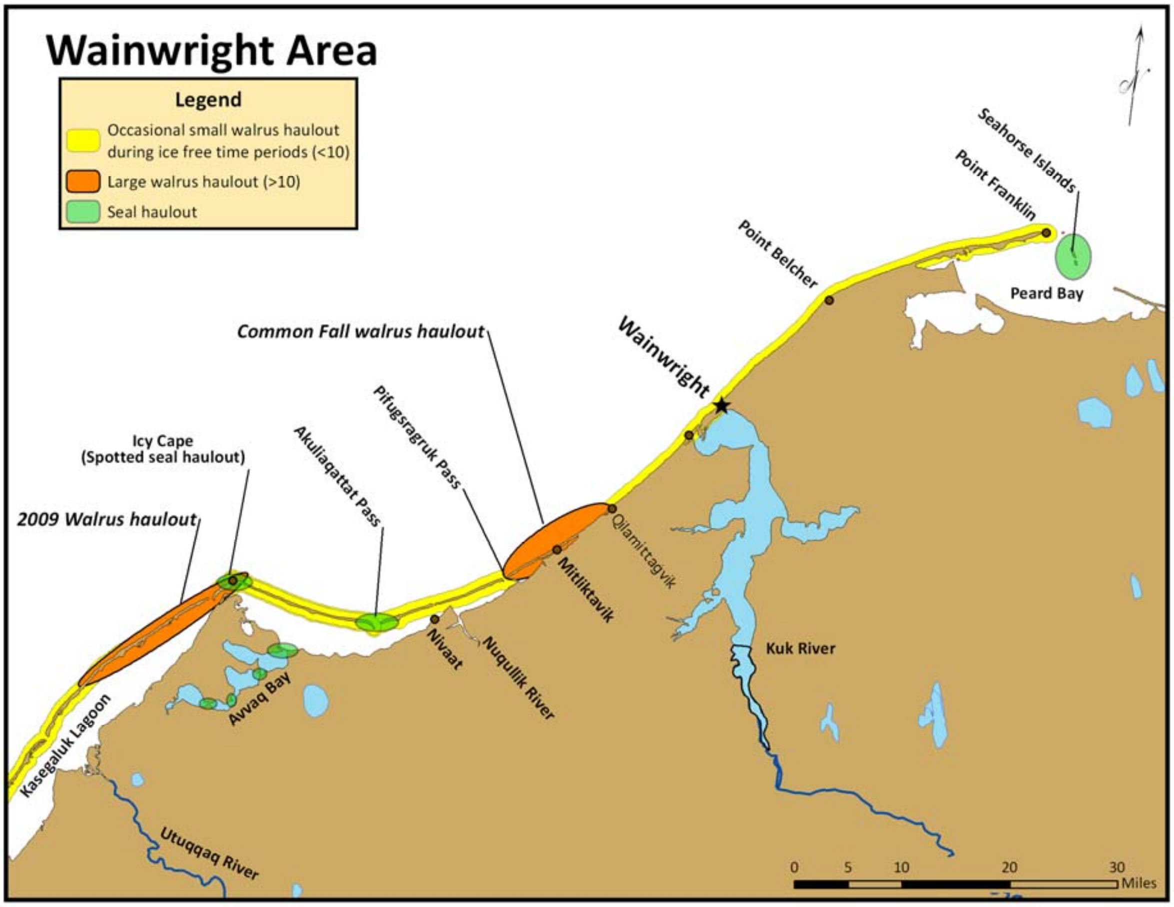


Figure 2. . Seal and walrus haulout sites as recorded during traditional knowledge interviews in Wainwright.

### *Walrus*

Walrus are seen in the Wainwright area primarily in spring and fall. In May, June, and July, they are seen on the ice moving northwards, though they are now rarely seen in July because the ice has started breaking up earlier. In the 1940s, 1950s, and into 1960s, walrus were brought near land by south winds in spring on ice floes right in front of the village; 1964 was the last time this happened. The ice floes would appear brown like land due to the number of walrus on them, and hunters would select smaller herds to hunt. Today, fewer walrus are seen hauled out on top of the ice. Hunters go far offshore in search of walrus after the ice breaks up. Formerly, the ice would leave shore but return at various times, each time bringing more seals and walrus back with it. Now, the ice leaves shore in June rather than July and does not return, making for a short

hunting season for ice-associated seals and walrus. Hunters have had to pursue walrus in the water rather than on the ice. In about 2008, no walrus were seen at all, having migrated past the Wainwright area before the shorefast ice broke up and boating was possible. Last year, hunters went 40 miles offshore but did not see walrus.

In fall, a few walrus are seen hauled out on the beach in both directions from Wainwright, usually alone or in groups or two or three. Larger groups are occasionally seen hauled out on shore. In the late 1940s, a group of skinny, apparently ill walrus were seen on the Seahorse Islands in Peard Bay; these islands were a common haulout area during that time. About 40 years ago, ~3,000 walrus were seen hauled out at Cape Sabine. This was regarded as unusual. Near Point Franklin, 10 or so walrus hauled out in fall is a regular occurrence. In the late 1990s, some walrus occasionally hauled out at the western end of Peard Bay. More recently, hunters saw a lone, old, sick walrus there. In 2009, many walrus hauled out at Icy Cape, but this too was unusual, a response to lack of ice. A walrus was once seen in the Kuk River lagoon behind Wainwright. In fall, walrus are much more alert and wary than in spring, fleeing the hunters' approach rather than just staying on the ice, making them harder to hunt.

Walrus are seen in winter, though rarely. They maintain breathing holes like seals do. These walrus appear to be seal-eating walrus, living off of the seals that are also present all winter.

Walrus in the water are dangerous to boaters, especially juvenile walrus. Larger herds are also more dangerous to people. Walrus are especially protective of their pups, so hunters are instructed by their elders to avoid hunting groups of females that might have pups with them. Hunters are also told not to hunt additional walrus after they have caught some already and are butchering those, or the other walrus may become aggressive and attack. When today's elders were young, two of them shot at walrus while the older men were butchering walrus already. The other walrus surrounded the floe, putting their tusks up on the ice, threatening to start chipping the floe away. The older hunters admonished the younger men, and then spoke to the walrus in Iñupiaq, and the walrus went away. Wounded walrus may also attack hunters.

A "dog" walrus is sometimes seen. This is a white walrus, with red eyes and a very long tail. It commands the other walrus, using its tail like a whip to control the others. It can give the signal to attack, so hunters are wary of the dog walrus.

Walrus on land are sensitive to disturbance, so the community tries to avoid large haulouts. If a person approaches a large group on land, the walrus may stampede, possibly crushing younger animals. Wainwright residents admired Point Lay's approach to managing access to the haulout there in the fall of 2010.

In the 1940s and 1950s, when walrus would come close to the beach on the ice, the older hunters would tell everyone to keep quiet in the village, so as not to disturb the walrus. The older hunters emphasized respect for the animals, taking only what you would eat and not more. They were very strict in their teaching and their own behavior. Today's hunters have a responsibility to pass those lessons and values on to the younger generations, to teach them how to care for the animals that have sustained the Iñupiat for thousands of years and which the elders and hunters hope will sustain people here for thousands more years.

Walrus today are in good physical shape, healthy and good to eat. Walrus that are hauled out alone or in small groups may be sick or tired, perhaps from having to swim so far from their feeding areas. This is especially hard for young walrus, whereas older adults can survive longer swims and longer periods without food. Mother walrus will sometimes carry their pups on their backs while swimming.

In the early 1960s, hunters returning from taking walrus on the ice near Point Franklin encountered a long line of walrus feeding underwater on the seafloor. The hunters, knowing the walrus were dangerous, had to detour far offshore to get around the feeding walrus.

Walrus usually have clams in their stomachs, but octopus and strips of ringed seal have been found, too. Walrus can rest in the water as well as on ice and on land. They inflate their throats with air, which keeps them floating with their nostrils above water, and they can sleep in the water.

Hunting patterns have changed over the years, largely in response to larger boats and more powerful outboard motors. Hunters used to work together with several boats, but now it is common for boats to travel alone when hunting walrus in the ice.

## *Seals*

Seals haul out on land. They haul out all along the beach in both directions from Wainwright, sometimes going up on the bluff. Spotted and bearded seals have been seen hauled out near Icy Cape since at least the 1930s. In the summer of 2010, many young ringed seals were hauled out on the beach. Some died, some were just very tired. Perhaps this was due to the lack of ice. The cause of death was unclear, and seagull predation prevented the collection of fresh carcasses for further examination.

Bearded seals swim up rivers.

Ringed and bearded seals have thinner blubber now than they used to. The blubber is also a different color, more yellow than it used to be. The taste of the meat has also changed, and this is true of many subsistence species, including marine mammals and waterfowl.

## *Other Information*

Hunters are concerned about the possible impacts of offshore activity, including ship traffic and oil drilling. The activity may displace walrus and other animals, moving them farther from their feeding grounds. If the walrus are pushed away from shore, they will be even harder to hunt. If they are pushed closer to shore, they will have to swim a long way to feed. Changing sea ice is not the only thing affecting walrus.

If animals are harder to hunt, people’s diets may change, too. Although there are concerns about contaminants in subsistence foods, hunters also like the taste of their traditional foods and know that those foods are nutritious, too. There were many concerns about the potential for radiation from the nuclear power plant disaster in Japan to reach Alaska and the animals people eat.

In the summer of 2010, there was a red tide algae bloom between Barrow and Wainwright. This was the first, or one of the first, times such an event was seen here. The hunters are concerned that the algae could affect mollusks, shrimp and krill, and the entire foodweb.

There was also concern about the potential impacts of polar bears and other species being listed as threatened or endangered under the Endangered Species Act. Hunters were concerned about the potential for regulation of subsistence hunting for marine mammals, and also about the effects of critical habitat designations on the community.

The ice is thinner in recent years than it used to be. Formerly, large floes of multi-year ice would arrive in fall and serve as “anchors” to the newly forming ice, creating a solid shorefast ice zone and allowing the ice to grow thick. Now, the thinner ice can be carried away whenever the water rises.

Killer whales will respond to calls for help from hunters. If walrus are threatening hunters, killer whales will come and scare the walrus away, allowing the hunters to return home safely.

Animals understand when they are spoken to in Iñupiaq.

Hunters are concerned about the state of their ice cellars, which are being affected by climate change. The City of Wainwright is considering purchasing some large freezers for community use, but these may not have the right temperature and variation in temperature to allow the foods to age properly to produce the delicacies that people greatly enjoy.

## Acknowledgements

We appreciate the support of the Eskimo Walrus Commission for this project and are grateful to commissioners Willard Neakok, for identifying participants and helping to set up interviews in Pt. Lay, and Enoch Oktollik for similar assistance in Wainwright. The Bureau of Ocean Energy Management (BOEM) funded the work as part of BOEM AK-09-01 and we appreciate the support of Charles Monnett and Catherine Coon. Justin Crawford prepared the maps used during the interviews and the figures in this report.

## References

Huntington, H.P. 1998. Observations on the utility of the semi-directive interview for documenting traditional ecological knowledge. Arctic 51(3):237-242.

Noongwook, G., the Native Village of Gambell, the Native Village of Savoonga, H.P. Huntington, and J.C. George. 2007. Traditional knowledge of the bowhead whale (*Balaena mysticetus*) around St. Lawrence Island, Alaska. Arctic 60(1):47–54.
