# Supplementary material for: Effects of changing sea ice on marine mammals and subsistence hunters in northern Alaska from traditional knowledge interviews
Source: Biol Lett. 2016 Aug;12(8):20160198. doi: 10.1098/rsbl.2016.0198 (PMC5014017; doi:10.1098/rsbl.2016.0198)
Supplement: Traditional Knowledge Regarding Walrus near Point Hope, Alaska [file rsbl20160198supp4.pdf]

Traditional Knowledge Regarding Walrus near Point Hope, Alaska

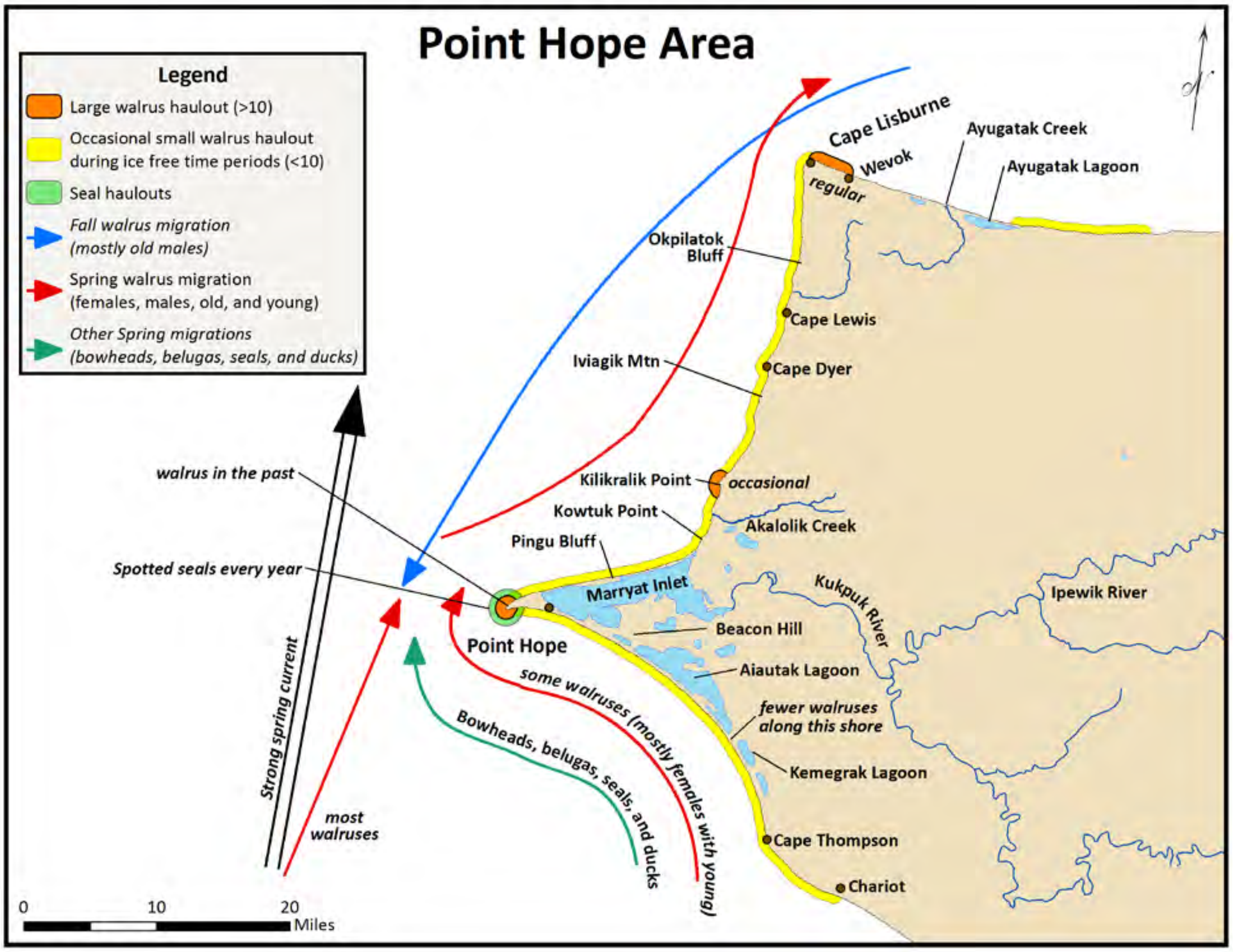

By:
Henry P. Huntington
Huntington Consulting
Eagle River, Alaska
hph@alaska.net
Ph: (907) 696-3564
and
Lori T. Quakenbush
Alaska Department of Fish and Game
Fairbanks, Alaska
lori.quakenbush@alaska.gov
Ph: (907) 459-7214

November 2013

This report is part of a project titled “Walrus Habitat Use in the Potential Drilling Area (Chukchi) - Village-Base Walrus Studies, contract #M09PC00027 funded by The Bureau of Ocean Energy Management (BOEM) and should be cited as follows:

Huntington, H.P., and L.T. Quakenbush. 2013. Traditional knowledge regarding walrus near Point Hope, Alaska. Report to Native Village of Point Hope and Bureau of Ocean Energy Management for contract #M09PC00027. 9 pp.

Traditional Knowledge of Walrus along the Northern Chukchi Sea Coast, Alaska: Point Hope

Introduction

Pacific walrus are an important species for subsistence harvests by Iñupiat hunters in northern Alaska. They are also an iconic Arctic animal, and at risk from climate change. Increasing industrial activity in the Chukchi Sea is an additional potential stressor to the walrus population. The study of walrus distribution, behavior, and movements is an important contribution to monitoring the effects of a changing environment and the potential effects from industrial activity. Placing satellite transmitters on walruses provides detailed information about how some individual animals are moving and behaving now. Documenting traditional knowledge about walrus, through interviews with residents of coastal communities, provides complementary information about general patterns in walrus distribution, movement, and behavior in the present and in addition provides a way to compare the present with the past.

This report summarizes information gathered from interviews with hunters in Point Hope, Alaska, in January 2013. This traditional knowledge project used the same approach that the Native Village of Savoonga used when documenting traditional knowledge about bowhead whales on St. Lawrence Island (Noongwook et al. 2007).

Methods

We used the semi-directive interview method, in which the interviewers raise a number of topics with the person being interviewed, but do not rely solely on a formal list of questions (Huntington 1998). Instead, the interview is closer to a discussion or conversation, proceeding in directions determined by the person being interviewed, reflecting his or her knowledge, the associations made between walrus and other parts of the environment, and so on. The interviewers use their list of topics to raise additional points for discussion, but do not curtail discussion of additional topics introduced by the person being interviewed.

The topics identified by the research team in advance of the interviewers were:

Walrus distribution and abundance near communities
Distribution and sightings of walrus throughout the year
Walrus haul-out patterns on land
Sensitivity of walrus to various types of disturbance
Haul-out patterns of other pinnipeds
Changes over time for all of the topics

The results are presented under different headings, reflecting the actual information collected and the fact that some of the subjects blend together, especially changes seen over time in regard to all of the topics. The interviewers were Henry Huntington and Lori Quakenbush. Lori Quakenbush is also the project leader.

Point Hope

In Point Hope, we interviewed eight people during three interviews; one with two hunters, one with six hunters (including one of the two from the first interview), and one with one hunter. Hunters interviewed included Theodore Frankson, Jr., Isaac Killigvuk, Sr., Henry Koonook, Ronald Oviok, Sr., and four others who chose to remain anonymous. Interviews were conducted on 10 and 11 January 2013 at the Qalgi Center in Point Hope. Five hunters shared the number of years that they have been observing walrus; the average was 40 years with a range of 26 to 50+ years.

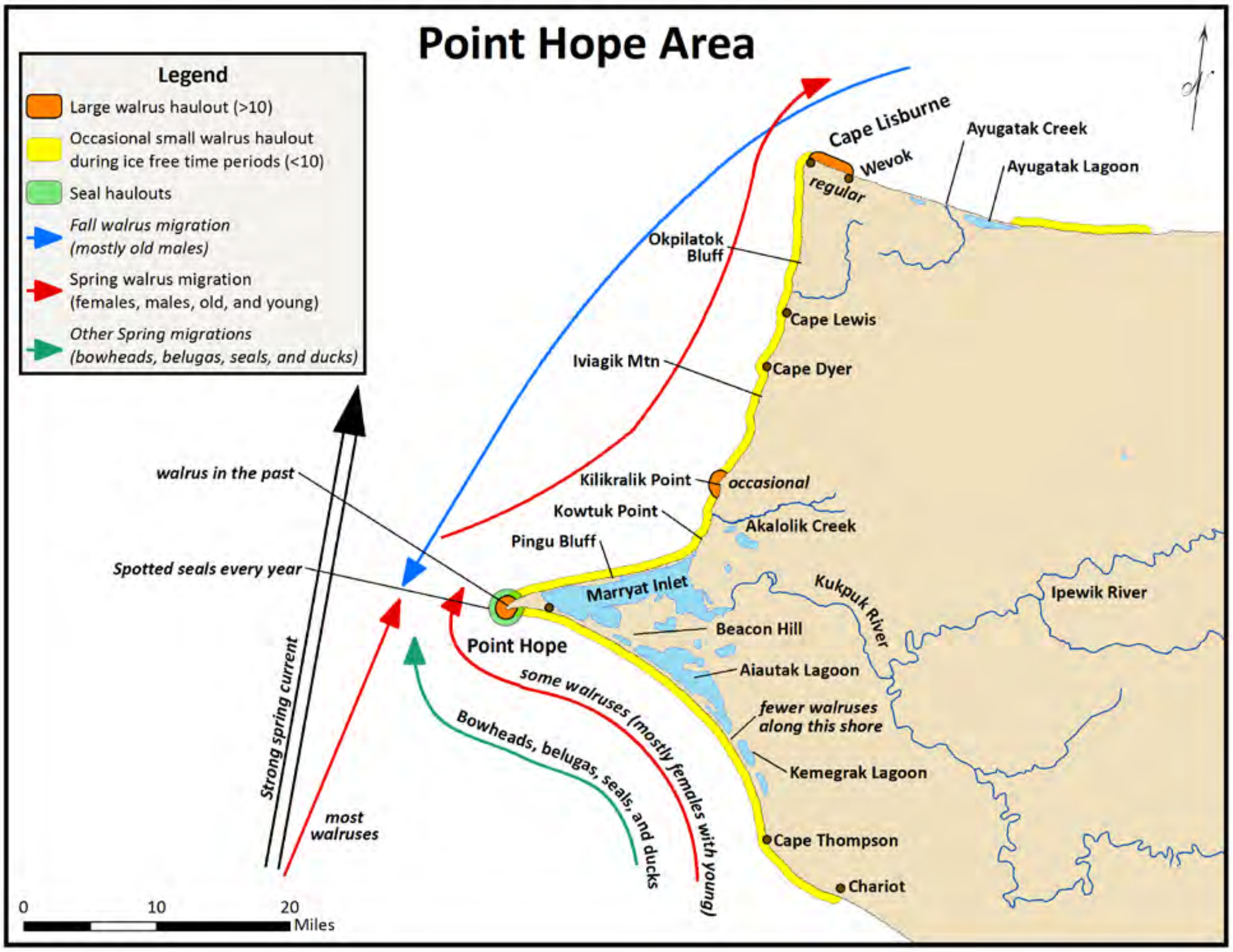

Figure 1. Seal and walrus haulout sites and general walrus migratory routes as recorded during traditional knowledge interviews in Point Hope. Other marine mammals and birds are also noted.

Walrus

Walrus typically arrive in the Point Hope area in May, after whaling season. Sometimes a few walrus have been seen migrating past in early spring, before whaling. Oddities like this happen from time to time. June and July is when they really start coming, after the bowheads and belugas have gone past. Some walrus migrate close to the shore, coming from the southeast, either riding on ice floes or swimming in open leads or cracks in the ice close to shore. They can sometimes be heard well in advance of their arrival near the village. Most walrus arrive from the

south or southwest, coming close to land only when they reach Point Hope itself. Hunters call them in by making sounds like a dog with a sore throat. Female walrus swim past Point Hope; there are also many seals here then, as long as there is still ice. Walrus are occasionally seen swimming south in spring. The spring migration includes walrus of all kinds: male, female, old, and young.

In the past five or so years, the walrus have not been coming as close to the point as usual, but appear to be traveling farther offshore as they pass Point Hope. Fewer males are seen in spring now, and fewer walrus overall. Thousands used to be seen, and now only a few hundred each year. Some hunters attributed this change to greater use of outboard engines as well as increased ship traffic in the region. The walrus are affected not just by the noise of the boats, but also by the smell of the fumes from the fuel and the engines. The shift in location also coincides with changes in sea ice, notably the loss of thick ice in winter (although the winter of 2011–2012 had thick ice as used to be the case every winter). Walrus may also be following the paths of ships that go through the ice in late spring.

In late September and October, the walrus migrate from the north, past Cape Lisburne and Point Hope. At this time of year, most of the walrus seen are older males. Fewer walrus are seen in fall than in spring. Few walrus were seen in fall 2012, only a few groups of a few walrus. A “winter walrus,” also known as an “aivialuq,” or as a “king walrus” is sometimes seen in the ice. These are very large animals.

During the fall migration, many walrus haul out on land just to the east of Cape Lisburne. This is a regular haul-out, with perhaps dozens of animals on land at any given time. In recent years, there have been fewer walrus here, perhaps due to barge traffic. Barges occasionally wait at Cape Lisburne for weather or ice, and they can disturb the walrus and keep them away.

Walrus occasionally haul out in similar numbers about halfway between Cape Lisburne and Point Hope, just to the north of Kilikralik Point. On at least one occasion in the 1940s, more than 60 walrus hauled out at Point Hope itself. Many were hunted, and people ate walrus for breakfast, lunch, and dinner. Walrus have not hauled out at the point recently. Most of the walrus that haul out on land are males.

One or two walrus may haul out anywhere along the shore from Cape Thompson to Point Hope to Cape Lisburne. This also occurs east of Ayugataq Lagoon, to the east of Cape Lisburne.

Some of the individual walrus coming to shore are following small seals, which they kill and eat. Only older male walrus eat seals. Elders said that walrus would sometimes come out of the water onto the ice to take seals. A hunter was once charged by a walrus on the beach. The walrus came very close, but then turned and went back in the water.

In the late 2000s, some people noticed yellow coloring on the flippers and around the mouths of a few walrus. Some of these drifted back in from the north side of Point Hope during the spring migration. This behavior was not unusual in itself, but due to the yellow coloring the elders said not to eat these animals. The yellow coloring has not been seen any other time. The Point Hope hunters who were interviewed have not seen walrus with green flippers or lips.

Hunters are seeing more skinny and sick walrus in spring in recent years. One male walrus taken in the fall of 2012 was ill. One walrus taken in spring 2012 had a hole in one side of its skull, although no obvious bullet hole in the hide. Its face was swollen and it smelled like a rotten tooth or worse. This animal was skinny.

A walrus with a satellite transmitter was taken in fall 2011. The walrus was healthy, and there did not appear to be any problems at the place where the transmitter was attached to the walrus’s skin.

People hunt whales and then bearded seals, for the skins to make umiaq covers, and then after getting the bearded seals they need, they start hunting walrus.

People in Noorvik saw a walrus in the Kobuk River two or three years ago. Noorvik people said there was a monster in the river, as they were not used to seeing walrus.

Seals

In spring, seals and polar bears come to the Point Hope area before whaling season. They can be found in the Point Hope area during winter.

Spotted seals haul out at the tip of Point Hope every year in the summer and mainly in the fall. The group is usually about 20–30 seals. Spotted seals are usually seen on the south side of the point. About fifteen years ago, hunters traveling by boat saw about 300 spotted seals hauled out on the ice on the north side of Point Hope. They had not seen such a large aggregation of spotted seals on the ice before.

Some hunters prefer the meat of ringed and bearded seals to that of spotted seals but they do use the skin and blubber of spotted seals. One hunter said he liked the meat of spotted seals better than ringed seals. Spotted seal meat has a very strong taste. Spotted seals are much smarter than other seals. Occasionally, a black spotted seal is seen. A group of spotted seals was once seen scaring a single walrus off the beach.

Ringed seals sometimes haul out on the beach in summer. These are usually young seals. Ringed seals haul out on the ice all around the Point Hope area.

Ringed seals may swim into Marryat Inlet and up the Kukpuk River, hauling out on the bank. Spotted seals have also been seen upriver. This is new behavior, going so far up the Kukpuk.

In 2011, many small ringed seals were seen on the beaches near Point Hope, with sores around their eyes and mouths and on their tails. They were very skinny and did not move when people approached, unlike healthy seals. About half a dozen of these sick seals were seen in the fall of 2012 as well. Hunters did not want to touch these sick seals. One hunter described the lesions on the seals as looking like “scratches” on the face and hind flippers. When hunters were taking seals in June and July, they noticed small, fast-moving bugs on the hind parts of the seals. This was new. A few sick seals and bearded seals are typically seen in early summer (late May through July).

One hunter wondered if the hair loss on seals could be due in part to sun exposure. He said that the hairless areas appear to be the areas that would be in the sun when seals are hauled out on the ice in spring. He once saw a ribbon seal with hair loss. Another hunter was worried about the melting of the ice cap making the water more fresh and causing problems.

Seals seen in winter appeared healthy. Many ringed seals are being taken now (January 2013) from the ice edge. During south and east winds there is good winter seal hunting around Kowtuk Point. Ringed seals are abundant and healthy in the Point Hope area.

In March, ringed seals taste like stove oil, due to the rut.

In the late 1960s, a hunter caught a pregnant female ringed seal that had a two-headed fetus.

According to the hunters who were interviewed, the women in Point Hope say that the seal oil is changing because the blubber has been getting darker in color, though it is still edible. The blubber is darker yellow or darker pink, and sometimes with some green color. This change, from orange-yellow blubber to a greener color, has been happening over the last five years or so.

Bearded seals have not been seen on the beach, but they do haul out on the ice in spring. Bearded seals are more commonly seen on ice to the north of Point Hope, but they are seen on ice to the south, too.

In the spring of 2012, two bearded seals were caught that were skinny, with yellow blubber. When hunters tried to move one of them, its whiskers pulled out by hand, instead of needing to be pulled out by pliers as is normal.

Other Information

Point Hope is a good location for hunting because there is open water on the south side if the wind is from the north, and on the north side if the wind is from the south. Point Hope hunters are patient—they go out and wait for the animals to come. The past year, 2012, was a good year for hunting, in that the animals were healthy and edible. There was, however, much erosion along the shoreline.

In spring, all marine mammals come past Point Hope. Hunters let the first animals of each species go past, and then start hunting. This is similar to leaving a few eggs in nests when gathering eggs, to provide for the future.

Every summer, killer whales come past Point Hope, but they are not seen as much anymore. Hunters would talk with killer whales. One does not have to talk loudly. Sometimes people would give a piece of maktak or blubber to the killer whales, but otherwise people would not bother them. Killer whales eat other whales. People have seen killer whales attacking gray whales. On one occasion, two killer whales grabbed the gray whale’s flippers and pushed it to the beach, while a third killer whale ate the gray whale’s tongue. On one occasion, a bowhead whale carcass washed ashore on the north side of the point, with killer whale bite marks and no tongue.

A Steller sea lion was caught just north of Point Hope in the 1980s.

A live elephant seal was seen at the point in the 1980s.

A narwhal carcass was found washed up on the beach north of Point Hope in the 1970s.

Two dead minke whales were found under the ice two to three years ago.

In spring 2012, there were many polar bears on the ice during whaling season. One whaling captain saw 12 polar bears near his camp at one time. The bears were most large males, and appeared healthy. The ice that spring was thick and solid, the way it used to be every year.

In spring 2012, hardly any fish came past Point Hope. People usually get salmon, but not in the summer of 2012.

In October 2012, blue tomcod washed up on the north side of the point. They had not been seen for some time. These are the fish that seals eat. Many types of invertebrates also washed up, including clams and mussels.

Point Hope used to catch small tomcod, but now they are getting larger ones, similar to those caught at Kotzebue. This change started a couple years ago.

Recently, fish were seen acting oddly on the north side of the point. They were swimming ashore, trying to get out of the water. Some of them had lesions, like something was eating away the scales and flesh on their sides.

Once in a while, people see small, skinny trout coming by. These are believed to be from the rivers near Kivalina or from the Noatak.

A live shark washed up on the south side of the village, near Beacon Hill. Another shark was found on the north beach.

Caribou do not come as close to Point Hope as they used to. The caribou used to go all the way to the point, past the old village site. Hunters have seen a small plane scaring the caribou away. A recently installed U.S. Coast Guard navigational beacon at Beacon Hill also appears to prevent caribou from coming farther west. This station is about five miles east of Point Hope. It has a light on it, and the light appears to be responsible for scaring the caribou.

In fall 2012, there were many wolves near Point Hope. One was taken in summer near the snow fence on the edge of the village.

Hunters are struggling now to get the animals they need. There is too much traffic in the air and on the sea. Too many ships travel close to the point, in the migratory path of marine mammals and where people hunt. This deflects the animals away from the point, making them harder to get

and requiring longer trips that use more gas and thus are more expensive. This is a big concern for Point Hope hunters.

Sometime in the 1980s there was a really loud ship in the Point Hope area. Seismic testing between Point Hope and Cape Lisburne resulted in a loss of tomcod and clams in the Point Hope area, and created a wider environmental disturbance. Seals eat the tomcod, so the loss of fish affected the seals. Walrus eat clams, so the loss of clams affected the walrus. The walrus and seals appear to have moved to other places, since fewer are seen now.

There was too much rain this summer; it was not a good berry year.

In spring there is a very strong north current about 10–15 miles off Point Hope. Hunters try not to travel that far offshore, to avoid being carried north.

The ice in the winter of 2012 was 5–7 feet thick, as it used to be before the ice started thinning. But it left in May and did not return. The ice used to come and go in summer, but now it leaves and is gone until fall.

Around 1970, there was a big surge of ice on the north side of the point, pushing ice as far as the ice cellars, which had not been seen for a very long time.

In 2011, after the tsunami in Japan, sea ice piled up on the beach on the south side, making pressure ridges that were 15–20 feet high. These were unusually far up the beach.

Trash from Russia and Japan washed up near Point Hope. Russian water bottles have been seen on the north side of the point, and Japanese trash on the south side. They have seen gas cans and floats, including one float that was covered in sticky oil.

Acknowledgements
We appreciate the support of the Eskimo Walrus Commission for this project and are grateful to Commissioner Ronald Oviok, Sr. and to Point Hope Mayor Steve Oomittuk and Teddy Frankson, Jr. of the Native Village of Point Hope for identifying participants and helping to set up interviews in Point Hope. We thank Teddy Frankson, Jr., Isaac Killigvuk, Sr., Henry Koonook, and Ronald Oviok, Sr. and four other hunters for sharing their knowledge and contributing to this report. The Bureau of Ocean Energy Management (BOEM) funded the work as part of contract #M09PC00027 and we appreciate the support of Catherine Coon. Justin Crawford prepared the maps used during the interviews and the figure in this report.

References

Huntington, H.P. 1998. Observations on the utility of the semi-directive interview for documenting traditional ecological knowledge. Arctic 51(3):237-242.

Noongwook, G., the Native Village of Gambell, the Native Village of Savoonga, H.P. Huntington, and J.C. George. 2007. Traditional knowledge of the bowhead whale (*Balaena mysticetus*) around St. Lawrence Island, Alaska. Arctic 60(1):47–54.
