# Supplementary material for: Effects of changing sea ice on marine mammals and subsistence hunters in northern Alaska from traditional knowledge interviews
Source: Biol Lett. 2016 Aug;12(8):20160198. doi: 10.1098/rsbl.2016.0198 (PMC5014017; doi:10.1098/rsbl.2016.0198)
Supplement: Traditional Knowledge Regarding Walrus, Ringed Seals, and Bearded Seals near Barrow, Alaska [file rsbl20160198supp5.pdf]

Traditional Knowledge Regarding Walrus, Ringed Seals, and Bearded Seals near Barrow, Alaska

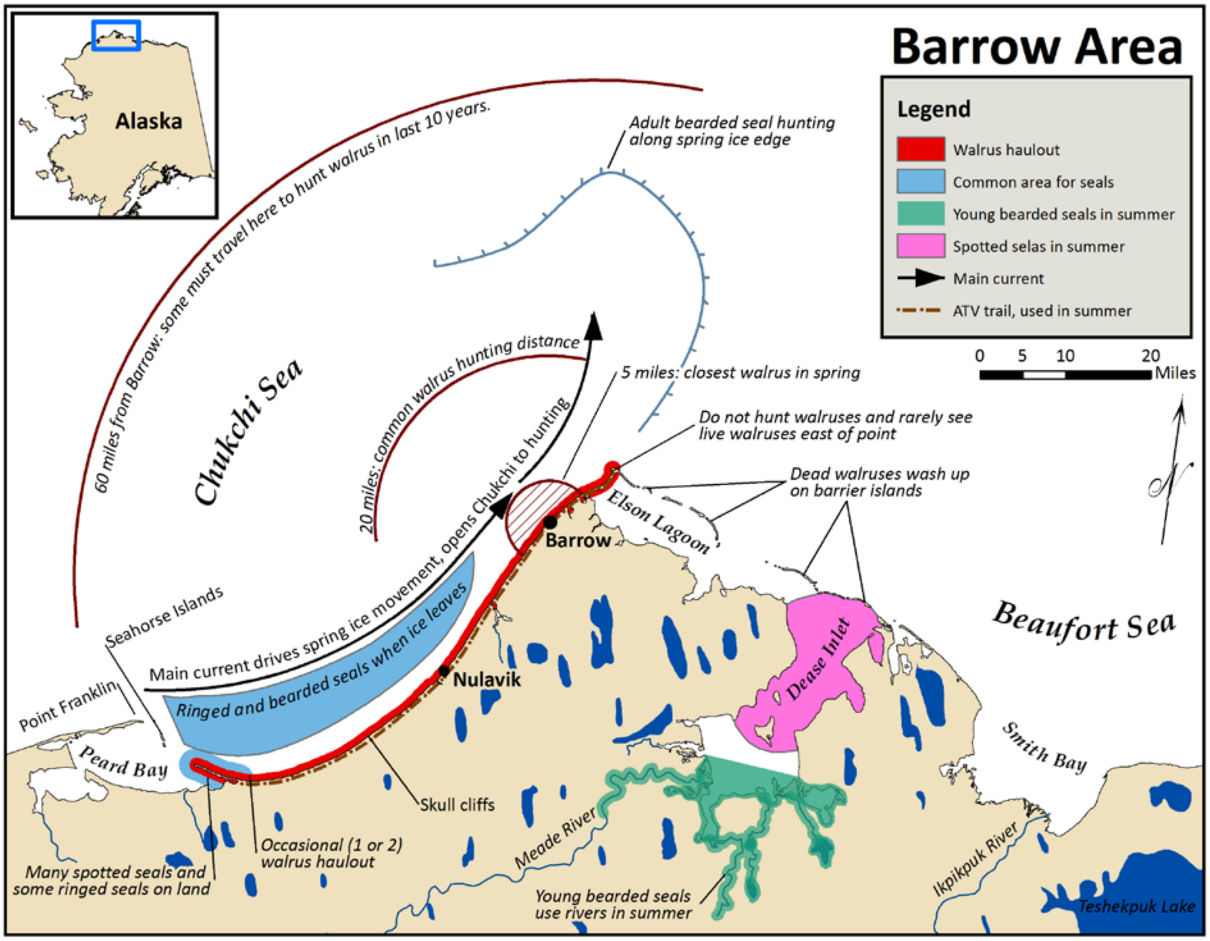

Traditional Knowledge Regarding Walrus, Ringed Seals, and Bearded Seals near Barrow, Alaska

By:

Henry P. Huntington
Huntington Consulting
Eagle River, Alaska
hph@alaska.net
Ph: (907) 696-3564

Mark Nelson and Lori T. Quakenbush
Alaska Department of Fish and Game
Fairbanks, Alaska
mark.nelson@alaska.gov, lori.quakenbush@alaska.gov
Ph: (907) 459-7374, (907) 459-7214

Final Report

Approved December 2015

Final report should be cited as:
Huntington, H.P., M. Nelson, and L.T. Quakenbush. 2015. Traditional knowledge regarding walrus, ringed seals, and bearded seals near Barrow, Alaska. Final report to the Eskimo Walrus Commission, the Ice Seal Committee, and the Bureau of Ocean Energy Management for contract #M13PC00015. 8pp.

Introduction

Walrus, ringed seals, and bearded seals are important species for subsistence harvests by Iñupiat hunters in northern Alaska. They are also iconic Arctic marine mammals, and at risk from climate change. Increasing industrial activity in the Chukchi Sea is an additional potential stressor to walrus and seal populations. A satellite telemetry study of the distribution, behavior, and movements of walrus and seals is an important contribution to monitoring the effects of a changing environment and the potential effects from industrial activity. While placing satellite transmitters on walrus and seals provides detailed information about the movements and some behaviors of individual animals, documenting traditional knowledge about walrus and seals, through interviews with residents of coastal communities, provides valuable complementary contemporaneous and historical information about the general patterns of each species.

This report summarizes information gathered from interviews with hunters and other knowledgeable residents in Barrow, Alaska, in January 2015. This traditional knowledge project used the same approach that the Native Village of Savoonga used when documenting traditional knowledge about bowhead whales on St. Lawrence Island (Noongwook et al. 2007).

Methods

We used the semi-directive interview method, in which the interviewers raise a number of topics with the person being interviewed, but do not rely solely on a formal list of questions (Huntington 1998). Instead, the interview is closer to a discussion or conversation, proceeding in directions determined by the person being interviewed, reflecting his/her knowledge, the associations made between walrus and other parts of the environment, and so on. The interviewers use their list of topics to raise additional points for discussion, but do not curtail discussion of additional topics introduced by persons being interviewed.

In Barrow, we interviewed ten people: one group of four, two groups of two each, and two individually. Those interviewed were Ernest Nageak, Van Edwardsen, Ronald Uyeno, Jonah Leavitt, Willie Koonaloak, John Heffle, and four people who wished to remain anonymous. The interviews were conducted on January 29, at the Iñupiat Heritage Center.

The topics identified by the research team in advance of the interviewers were:

- Haul-out patterns on land
- Observations of orphaned calves
- Timing and location of walrus and seal sightings
- Behavior of walrus and seals
- Parts of walrus and seals eaten by humans
- Changes over time for all topics

The results are presented under different headings, reflecting the actual information collected and the fact that some of the subjects blend together, especially changes seen over time in regard to all of the topics. The interviewers were Henry Huntington and Mark Nelson. Lori Quakenbush is the project leader.

Ringed and Bearded Seals

Ringed seals are generally found on the Chukchi Sea side of Point Barrow, including in front of the town of Barrow. They are usually found closer to shore and so are the first seals seen when boating out to hunt seals or walrus. When hunting from boats, Barrow hunters prefer to hunt bearded seals, passing ringed seals by, unless they are teaching a young hunter how to hunt for seals. When the ice is far out, seals may be found near river and creek mouths, where feeding is often good.

In spring, many ringed seals haul out on the ice. Ringed seals may haul out on land to rest. This can be seen south of Barrow towards Peard Bay. It is less common near Barrow, where there is often a lot of four-wheeler traffic. Ringed seals with bald spots and sores, which were most common in the summer of 2011, hauled out more frequently on the beach than ringed seals usually do. Seals with these signs of disease have been seen in subsequent years, too, but less often. The diseased seals are thin and do not flee an approaching person. Instead, it is possible to walk right up to them. Hunters avoid animals with signs of disease, so these animals were not hunted. Hunters also avoid ringed seals with black faces, as these seals taste like kerosene (Note: these are known to be adult males in rut, which develop a strong taste and smell).

Bearded seals are generally found farther from shore. They used to be found closer to Barrow. In summer, they may be 20–30 miles north of Point Barrow, along the ice or where the Chukchi and Beaufort waters meet. They are often plentiful that far out, but not seen as often closer to shore. While bearded seals are often associated with ice, they will remain in ice-free waters, too. They can be seen in front of Barrow, and juveniles are often seen off Elson Lagoon. In summer when there is no ice, bearded seals can be found in the current about 7 miles from shore. Hunters are taught to go out to the current and drift along until they see seals. Sometimes fewer seals are seen later in the summer. In these conditions, many boats may compete while chasing the same seal, which is not the way it used to be. The best hunting time for bearded seals is in July when the ice is beginning to go out.

Bearded seals can also sleep in open water. Hunters have come across bearded seals lying on the surface. When the seals are awakened by the boat, they react quickly, diving with a splash.

Bearded seals swim up the rivers that flow into Admiralty Bay. Bearded seals of all ages have been seen to do this. The seals are probably eating whitefish in the rivers. This is not a common occurrence however, most young bearded seals are found around the barrier islands and outside Dease Inlet during the summer.

In recent years, bearded seals have had thinner blubber. Hunters need to get to a seal quickly before it sinks. The seal oil they produce is also different from the way seal oil used to be.

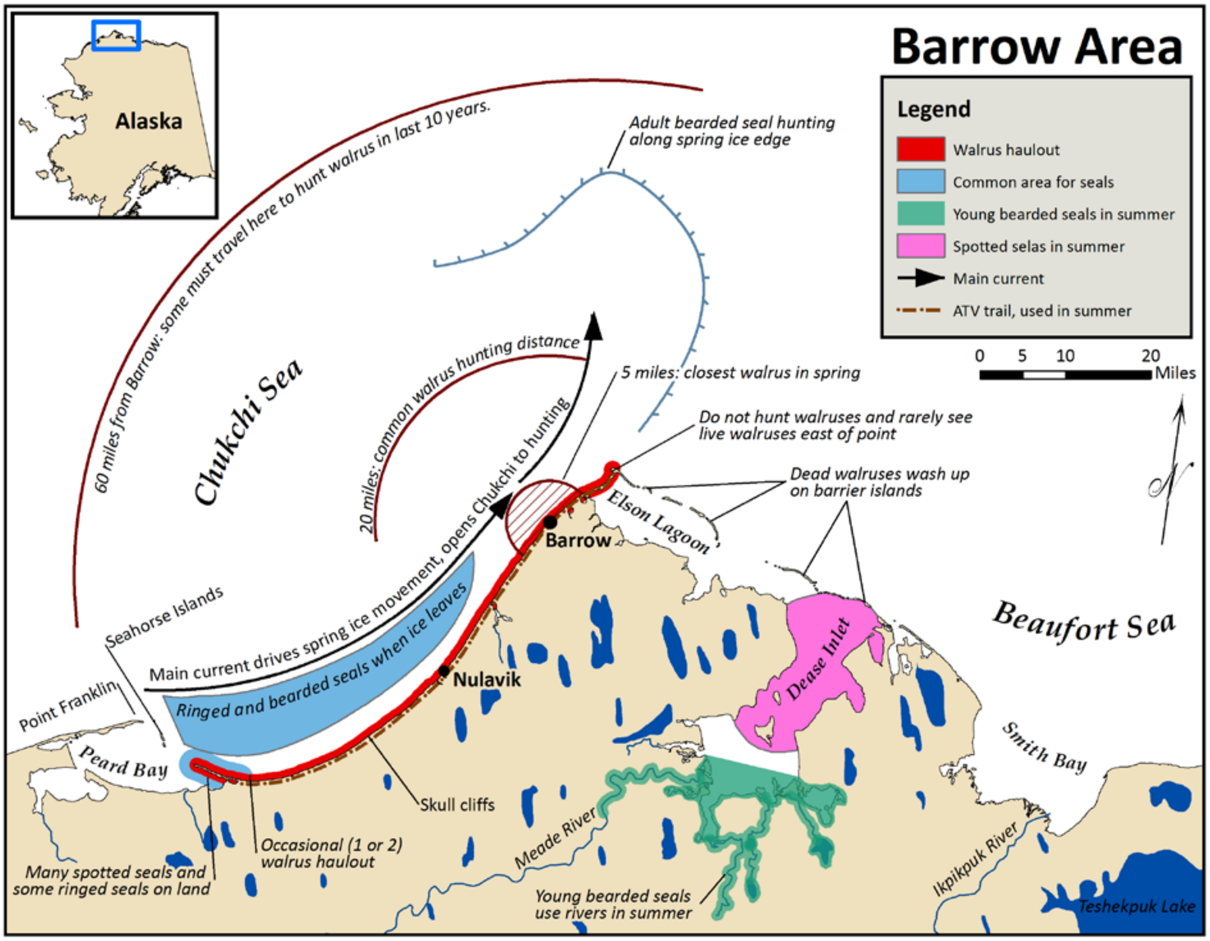

Figure 1. Movements and behavior of ringed seal, bearded seal, spotted seal, and walrus near Barrow as described during traditional knowledge interviews, January 2015.

Walrus

Walrus are harvested in Barrow when they are accessible. Walrus hunting occurs in the Chukchi Sea, but not the Beaufort Sea. Hunters typically head west, or first go south to Peard Bay and then offshore. Access depends primarily on ice conditions and can vary greatly from year to year near Barrow. Some hunters have gone as far as 60 miles offshore, which is possible now due to the fuel efficiency of four-stroke engines. Traveling this far, however carries risks if the weather changes for the worse. Recent changes in sea ice distribution and thickness have been the dominant factor responsible for changes in walrus distribution and behavior in recent years.

Walrus migrate north in spring, drifting with sea ice carried in the northbound currents of the Chukchi Sea. When shorefast ice breaks up, which is happening earlier and earlier, Barrow hunters are able to begin hunting by boat. East winds carry sea ice away from shore, making access difficult. West winds bring sea ice closer to the Barrow coast, bringing walrus and bearded seals with the ice. Walrus are typically carried north past the City of Barrow and onwards past Point Barrow. Only occasionally are walrus seen in the Beaufort Sea, to the east of Point Barrow. The eddies that form to the northeast of Point Barrow do attract beluga whales

and bearded seals, and may be responsible for walrus carcasses washing up on the barrier islands that border Elson Lagoon. Formerly, the ice would come and go during the summer as the winds shift, bringing ice and animals back several times, providing several hunting opportunities. In recent years, the ice usually does not return after it leaves the Barrow area and may leave faster when it goes out. The hunting season is thus shorter now, though still variable depending on the conditions of each year.

Ice thickness plays a role, too. Formerly, walrus were found on large ice floes in herds of up to 3,000 animals. Today, ice floes are smaller and thinner, so walrus are typically found in small groups (10–15 animals) or mid-size groups (50–100). Hunters could smell the large herds a long way away, but the smaller groups do not have as strong an odor. When walrus leave the ice floes, the floes rebound and rise higher out of the water. Thinner ice is noticeable at other times, too. During spring whaling, it is harder to find flat areas of ice that are thick enough to support a large bowhead whale for butchering. Formerly, any flat area was thick enough, but this is no longer the case.

Walrus are occasionally seen swimming in open water, presumably traveling from haulouts to feeding areas. This has been observed 4–5 miles offshore, with the walrus heading south, and no ice in sight. Individual walrus have been seen swimming along the shore in late August, with no ice in sight. Walrus can be hunted in open water, but it is much harder than hunting them when hauled out on ice floes, and they must be towed to ice or land for butchering. Walrus are also very dangerous when in the water. They can be aggressive and attack boats, and have been known to team up when doing so. This can occur when a walrus has been killed and the other walrus do not want to leave it. Dropping empty rifle shells into the water can scare walrus away, perhaps from the appearance or the sound.

Occasionally a single walrus, and more rarely two walrus together, will haul out on shore in the Barrow area. This is more common to the south of Barrow, towards Nulavik and Skull Cliffs, but can also be seen towards Point Barrow. Hunters have not seen three or more walrus hauling out together in the Barrow area. Walrus that haul out near Barrow are usually hunted; they will be seen by people who travel up and down the coast by four-wheeler, a common summer activity. Once, a sick walrus went inland from Elson Lagoon behind the Naval Arctic Research Lab (NARL) hangars. Sick walrus and sick polar bears will take themselves off to die. There does not appear to be a change in hauling out behavior in the Barrow area, although it may be more common in the past decade or so.

One hunter/carver recently saw a walrus skull that had many cavities in its teeth. He had not seen that before.

Orphaned calves are found occasionally in the Barrow area, but this is not common. It typically occurs after there has been a hunt. The last instance was three or more years prior to the interviews. When calves can be nursed to health, they are given to a zoo or other facility. There does not appear to be any trend in the frequency of orphaned calf sightings.

Walrus have excellent hearing. They do not react to people speaking in normal voices. Instead, they become suspicious if people are whispering or otherwise trying to be quiet. Walrus are

generally noisy animals, so the additional noise does not bother them. The same is sometimes true of other animals—they react more to people who are trying to sneak up to them or sneak past, than to those who show that they have seen the animals and are aware that the animals have seen or heard them.

Offshore oil and gas activity could have impacts to walrus and walrus prey. This could have a bigger impact on walrus-dependent communities, especially Gambell and Savoonga.

Barrow residents eat the skin, blubber, meat, kidneys, and heart of walrus. They do not typically eat liver or intestines. All parts are equally likely to be eaten by anyone eating walrus; there are no parts that are given specially to people of different ages or gender. Some people like to eat clams from walrus stomachs, but recently the stomachs have been mostly empty.

Polar Bears

Polar bears occasionally swim ashore in summer, having come a long way from the ice. They are typically exhausted. Polar bear monitors let them rest, rather than scaring them back into the water right away. Elders also say to leave animals alone when they come to shore, to let them rest. One bear collapsed on reaching shore and slept for a day or two, before getting up and walking to the Barrow bone pile.

Other Observations

Many things are changing. Skin boats left in the open in summer will now discolor and turn dark. They need to be covered with a tarp, whereas before they could be left out and would bleach in the sun.

Multi-year ice rarely shows up now, whereas it used to arrive reliably in October as the ocean began to freeze.

Traditional and Modern Knowledge and Ideas for Research

Younger hunters distinguished traditional knowledge from modern knowledge. The former is what can be learned from elders and others with long experience. Younger hunters have modern knowledge, having only hunted from motorboat and snowmachine, not with dog teams and other older equipment. They thus have different knowledge than their fathers and grandfathers, though they still have the skills to hunt effectively and to know how to interact with animals. The younger hunters are also well aware of how rapidly conditions are changing, and are able to provide knowledge about recent changes in a way that elders who have not hunted for many years may not.

The U.S. Fish and Wildlife Service has been paying more attention to traditional knowledge, which is a good thing and can be very helpful in many situations. At the same time, however, there are concerns about asking the same people the same kinds of questions over and over, or going to the same people to ask about different species but still taking up their time repeatedly. Better coordination in meetings and in research can help reduce the burden on the community, as it can on the ecosystem for field studies. Doing things once rather than several times would be better.

The U.S. Fish and Wildlife Service is also good at circulating information about their polar bear surveys, including photos of the aircraft involved, and flight plans and dates. This lets people know what to expect and how to recognize the aircraft and personnel who are involved, instead of wondering who is doing what and what impact they are having on hunters and on animals.

When walrus are being hunted from Barrow, there may be as many as 30–40 boats on the water. Finding ways to record the observations of these hunters could be a big contribution to walrus monitoring and research, and for other species, too. Hunters pay attention to oddities, such as unusual body condition or markings, and could take photos and bring this to the attention of biologists. Facebook might be a great way to organize hunters and report observations.

Acknowledgements
We appreciate the support of the Eskimo Walrus Commission and the Ice Seal Committee for this project and are grateful to Joe Sage and Thomas Olemaun for identifying participants and helping to set up interviews. The Bureau of Ocean Energy Management (BOEM) funded the work as part of Contract Nos. M09PC00027 and M13PC00015 and we appreciate the support of Charles Monnett, Catherine Coon, and Dan Holiday. Justin Crawford prepared the maps used during the interviews and the figures in this report.

References
Huntington, H.P. 1998. Observations on the utility of the semi-directive interview for documenting traditional ecological knowledge. Arctic 51(3):237-242.

Noongwook, G., the Native Village of Gambell, the Native Village of Savoonga, H.P. Huntington, and J.C. George. 2007. Traditional knowledge of the bowhead whale (*Balaena mysticetus*) around St. Lawrence Island, Alaska. Arctic 60(1):47–54.
