# Supplementary material for: Effects of changing sea ice on marine mammals and subsistence hunters in northern Alaska from traditional knowledge interviews
Source: Biol Lett. 2016 Aug;12(8):20160198. doi: 10.1098/rsbl.2016.0198 (PMC5014017; doi:10.1098/rsbl.2016.0198)
Supplement: ADF&G-ELI 2015 [file rsbl20160198supp6.pdf]

Traditional Knowledge Regarding Ringed Seals, Bearded Seals, and Walrus near Elim, Alaska

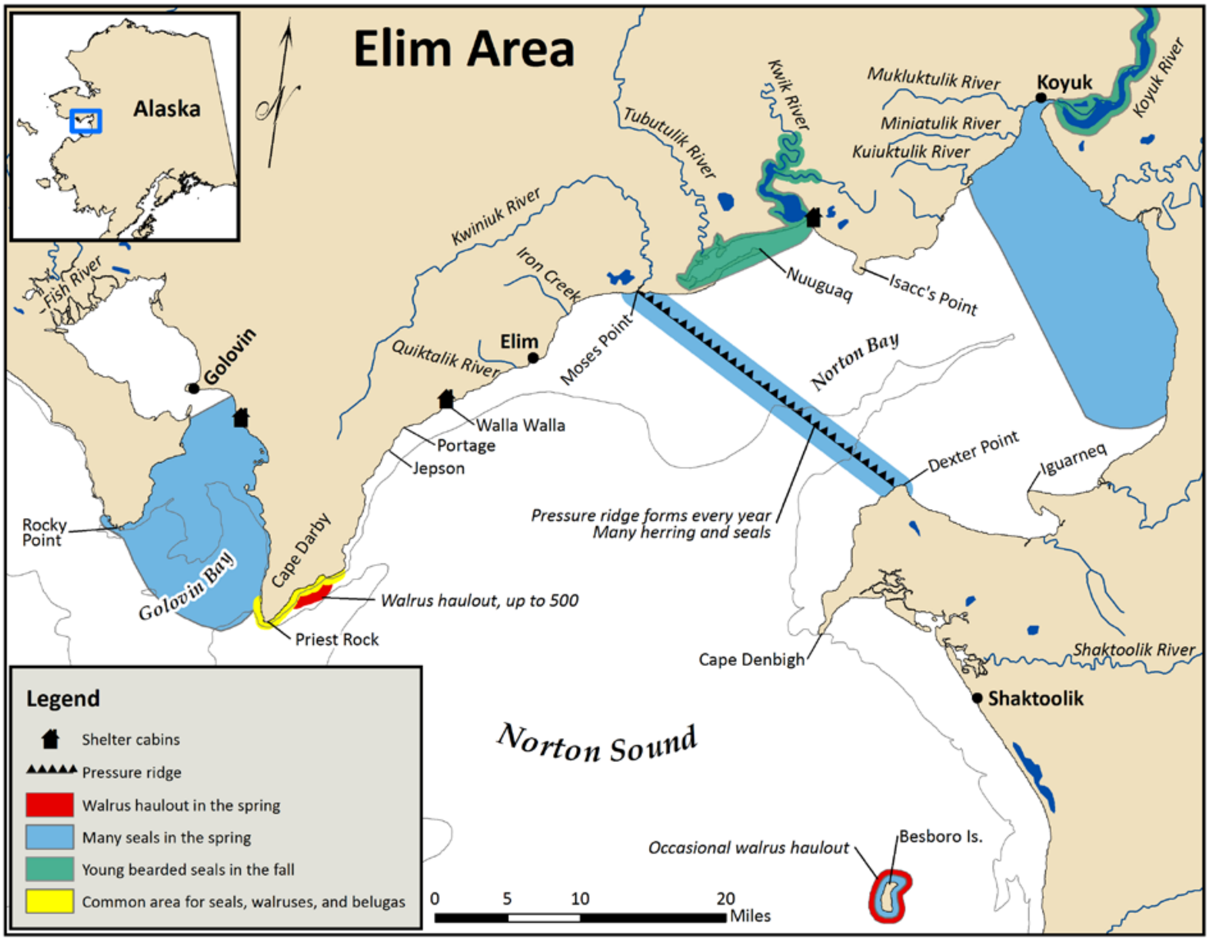

Traditional Knowledge Regarding Ringed Seals, Bearded Seals, and Walrus near Elim, Alaska

By:

Henry P. Huntington
Huntington Consulting
Eagle River, Alaska
hph@alaska.net
Ph: (907) 696-3564

Mark Nelson and Lori T. Quakenbush
Alaska Department of Fish and Game
Fairbanks, Alaska
mark.nelson@alaska.gov, lori.quakenbush@alaska.gov
Ph: (907) 459-7374, (907) 459-7214

Final Report
Approved December 2015

Final report should be cited as:
Huntington, H.P., M. Nelson, and L.T. Quakenbush. 2015. Traditional knowledge regarding ringed seals, bearded seals, and walrus near Elim, Alaska. Final report to the Eskimo Walrus Commission, the Ice Seal Committee, and the Bureau of Ocean Energy Management for contract #M13PC00015. 7pp.

Introduction

Ringed seals and bearded seals are important species for subsistence harvests by Iñupiat and Yup'ik hunters from Elim, in northern Norton Sound, in western Alaska. Walrus are found and hunted in this area, too. These marine mammals are iconic Arctic animals, and at risk from climate change. Increasing industrial activity in the Chukchi Sea, coastal development in the Norton Sound region, and shipping through the Bering Strait are additional potential stressors to seal and walrus populations. A satellite telemetry study of the distribution, behavior, and movements of seals and walrus is an important contribution to monitoring the effects of a changing environment and the potential effects from industrial activity. While placing satellite transmitters on seals and walrus provides detailed information about the movements and some behaviors of individual animals, documenting traditional knowledge about seals and walrus, through interviews with residents of coastal communities, provides valuable complementary contemporaneous and historical information about the general patterns of each species.

This report summarizes information gathered from interviews with hunters and other knowledgeable residents in Elim, Alaska, in February 2015. This traditional knowledge project used the same approach that the Native Village of Savoonga used when documenting traditional knowledge about bowhead whales on St. Lawrence Island (Noongwook et al. 2007).

Previous projects on traditional knowledge of seals have been conducted, one under the Elim-Shaktoolik-Koyuk Marine Mammal Commission in 1999 (Huntington 2000) and another on walrus and ice seals conducted by Kawerak Inc., in 2010–2013 in communities throughout the Bering Strait region, including Elim (Kawerak, Inc., 2013). Except as noted below, the information presented here comes from our February 2015 interviews and not from either prior project. A compilation of results from all three projects may be carried out later, to document changes over time and other aspects of seals and walrus.

Methods

We used the semi-directive interview method, in which the interviewers raise a number of topics with the person being interviewed, but do not rely solely on a formal list of questions (Huntington 1998). Instead, the interview is closer to a discussion or conversation, proceeding in directions determined by the person being interviewed, reflecting his/her knowledge, the associations made between walrus and other parts of the environment, and so on. The interviewers use their list of topics to raise additional points for discussion, but do not curtail discussion of additional topics introduced by the person being interviewed.

In Elim, we interviewed eight people in one group. Those interviewed were Darlene Katchatag, Martin Murray, Charles Saccheus, and five others who wished to remain anonymous. The interview was conducted on February 3, at the Elim IRA Council office.

The topics identified by the research team in advance of the interviewers were:

Haulouts on land
Overwintering areas and behavior
Use of lagoons and rivers
Feeding patterns and prey

Differences between ringed and bearded seals
Impacts from climate change
Parts of seals that people eat

The results are presented under different headings, reflecting the actual information collected and the fact that some of the subjects blend together, especially changes seen over time in regard to all of the topics. The interviewers were Henry Huntington and Mark Nelson. Lori Quakenbush is the project leader.

Ringed Seals
In early spring, ringed seals will lie on the sea ice by the hundreds throughout Norton Bay and stay in the area until the ice starts to break up more in the later spring. A few areas hold higher numbers of seals throughout the year and especially during the spring; Besboro Island, Golovin Bay, upper Norton Bay, Rocky Point and around Cape Darby. There is a persistent pressure ridge that forms from Moses Point to Dexter Point during the winter and spring that also holds many ringed and bearded seals. The snow is not as deep now as it used to be and there are fewer seal dens on the ice because of it, but there are still lots of seal breathing holes noticed mostly by fishermen who go out on the ice to deploy their crab pots in spring between Elim and Cape Darby. Pressure ridges like the one from Moses Point to Dexter Point are important for breathing holes and denning habitat. Ringed and bearded seals have always wintered in Norton Bay, especially near the mouths of creeks where fish are plentiful.

Ringed seals are occasionally seen hauled out on the beach. Usually this is young seals in the spring. Ringed seals do not haul out in large groups like walrus and are much more solitary. Historically in the spring and summer seals were taken with nets for subsistence, but this is less common now with most hunters choosing to use a rifle and harpoon instead. When a ringed seal is caught alive they are generally easy to control as they do not bite people. They will use their powerful claws to scratch at a person though.

Ringed seals feed on mostly fish such as herring, capelin, tomcod, skipjack (smelt, cisco?), and sometimes shrimp. Herring are found in large numbers along the pressure ridges especially between Moses Point and Dexter Point where they are sometimes pushed out onto the ice and preyed on by seagulls.

Elim residents eat the blubber (oil), meat, heart, kidneys, liver, and intestines of ringed seals. Seal oil is very healthy, especially for brain development in children. Coastal residents trade seal oil with interior Indians. A seal taken in November yielded clear seal oil with little flavor, so salt had to be added to give it more flavor.

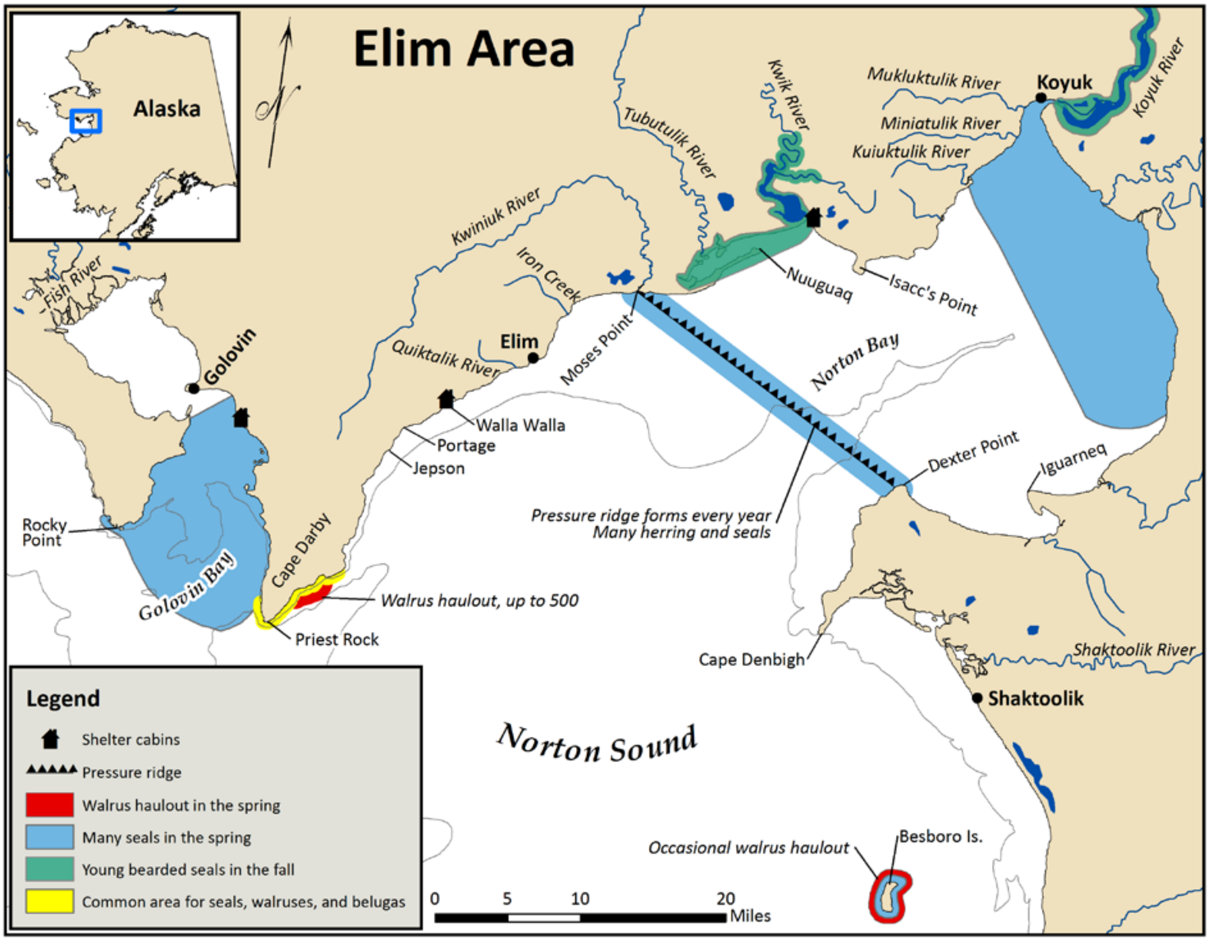

Figure 1. Movements and behavior of ringed seal, bearded seal, spotted seal, walrus, and beluga whales near Elim as described during traditional knowledge interviews, February 2015.

Bearded Seals

Bearded seals will haul out on the sea ice in spring, younger animals closer to shore on shorefast ice and older animals further out on the pack ice usually more than 15 miles from Elim. Young-of-the-year bearded seals will go up the Koyuk and Kwik Rivers in August and September. They eat clams, which can be found far upriver, and isopods. They are not found up the Tubutulik River, but can be seen in the lagoon at the mouth of the river. Adult bearded seals have been seen occasionally up the Kwik River, but not very often.

Bearded seals are more sensitive to noise than ringed or ribbon seals for example; if a ribbon seal on an ice floe is approached by hunters in a boat, it will flee away from the hunters across the floe, even if there is open water right next to it in the direction of the boat but bearded seals will dive into the open water instead. Hunters say that this behavior shows that bearded seals are much smarter and more wary than ribbon seals. Young bearded seals will fight back when caught in a net. They have long claws and will try to scratch a person, but they do not bite.

Bearded seals have clams, shrimp, and isopods in their stomachs. They do not have fish in their stomachs except for young bearded seals in rivers, they feed on whitefish. Old bearded seals have teeth that are worn down to the gums. Their blubber is yellow and yields yellow seal oil.

Elim residents eat the blubber (oil), meat, heart, kidneys, liver, and outer covering of the intestines of bearded seals.

Walrus

Walrus haul out occasionally on the east side of Cape Darby. This happens when there is no ice in Norton Sound. The walrus do not haul out on the west side of the Cape. There may be 500 or more walrus hauled out at a time. When there is no more room for walrus to haul out, other walrus will inflate their necks and float as they sleep, drifting in the current. After they drift a mile or two, they swim back to the haulout and repeat this behavior. There are sometimes some baby walrus in the haulout, in June. Walrus also haul out, though less frequently, on Besboro Island, in similar numbers to those seen at Cape Darby.

Walrus usually have clams in their stomachs. If the walrus has been on top of the ice for a while, the clams are partly digested. If the walrus has just hauled out on the ice after diving or still swimming, the clams will be fresh. These clams are ready to be cooked. Many people enjoy eating them.

Other Information

Qairaliq seals are no longer seen. These were described in the 1999 project (Huntington 2000) as small seals seen in April, May, and June, with thin skin that was useful for many purposes. The 1999 study reported that *qairaliq* seals came to the area in great numbers in the 1980s, but fewer were seen by 1999.

Elim hunters had no further information about *iigliq* seals, also reported in the 1999 study.

A bearded seal was once taken that had metal in the muscle near its ribs.

Green algae grows on the bottom of the sea ice, attracting fish. This has always happened.

In the summer of 2014 there were so many jellyfish they clogged fishing nets. The jellyfish were also larger than usual. Starfish taken in crab pots appeared to be eating jellyfish.

Small, pink krill are common throughout Norton Bay. They can be found under rocks and come to the surface with crab pots that are hauled up.

A few years ago, orange foam was found on the beaches throughout the area. The cause of this was not known and it only happened the one time.

Sea ice formed very late in the winter of 2014–15. There was a lot of open water near Unalakleet and even by Koyuk into December 2014. Beluga whales were seen swimming past Elim in January, being pursued by killer whales. It is very rare for belugas to be seen near Elim in January.

Belugas prefer to eat tomcod and shrimp, even if there are herring in the area. They also like salmon, which they eat all summer. Silver salmon in September are a favorite food of belugas. Belugas chase salmon and, when they are right behind the fish, give a blast of sound that stuns the fish, so the fish turns belly up. Then the beluga swallows the salmon whole.

Belugas can drown in a net in 10 minutes or less. They struggle, which uses up their air quickly. The belugas are used for subsistence and the lungs used to be fed to dogs. They feel spongy and were not eaten by people.

Spotted seals haul out in groups in many locations, including Rocky Point and have teeth like dogs. Spotted seals will bite people.

Animals carry their history, like human beings, including their own lives and the lives of their parents and grandparents. Animals have brains and spirits. It is essential to keep the ocean free and clean so that the animals can flourish and live healthy lives.

Concerns

Elim residents asked whether there have been any studies in the Nome area to determine if there are impacts from dredging and port construction. They expect more activity along the coastline of the region, and think it is important to learn what we can from the development that is occurring already. They pointed out that the dredging results in muddy water, which they expect affects marine mammals and other sea animals.

There is a lot more commercial crabbing in summer in the Norton Bay area now.

More hunters from other villages seem to be coming to the Norton Bay area to hunt.

Acknowledgements

We appreciate the support of the Eskimo Walrus Commission and the Ice Seal Committee for this project and are grateful to Charles Saccheus and Carol Nagaruk for helping to set up the group interview. The Bureau of Ocean Energy Management (BOEM) funded the work as part of Contract Nos. M09PC00027 and M13PC00015 and we appreciate the support of Charles Monnett, Catherine Coon, and Dan Holiday. Justin Crawford prepared the maps used during the interviews and the figures in this report.

References

Huntington, H.P. 1998. Observations on the utility of the semi-directive interview for documenting traditional ecological knowledge. Arctic 51(3):237-242.

Huntington, H.P. 2000. Traditional ecological knowledge of seals in Norton Bay, Alaska. Report submitted to the Elim-Shaktoolik-Koyuk Marine Mammal Commission and the National Marine Fisheries Service. April 2000.

Kawerak, Inc. 2013. Seal and walrus harvest and habitat areas for nine Bering Strait Region communities. Nome, Alaska: Kawerak, Inc., Social Science Program.

Noongwook, G., the Native Village of Gambell, the Native Village of Savoonga, H.P. Huntington, and J.C. George. 2007. Traditional knowledge of the bowhead whale (*Balaena mysticetus*) around St. Lawrence Island, Alaska. Arctic 60(1):47–54.
