# Supplementary material for: Effects of changing sea ice on marine mammals and subsistence hunters in northern Alaska from traditional knowledge interviews
Source: Biol Lett. 2016 Aug;12(8):20160198. doi: 10.1098/rsbl.2016.0198 (PMC5014017; doi:10.1098/rsbl.2016.0198)
Supplement: ADF&G-SMK SBB 2015 [file rsbl20160198supp7.pdf]

Traditional Knowledge Regarding Ringed Seals, Bearded Seals, and Walrus near St. Michael and Stebbins, Alaska

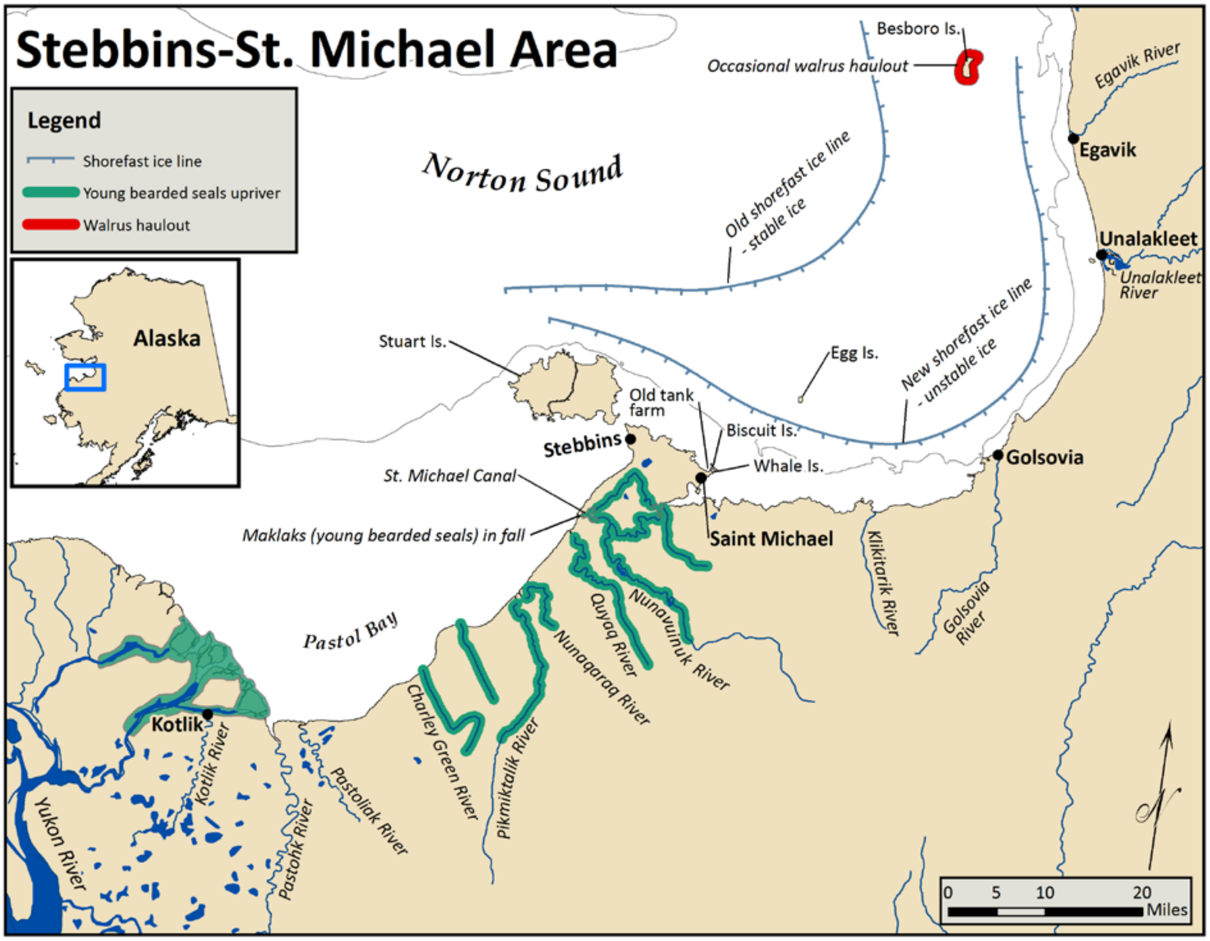

Traditional Knowledge Regarding Ringed Seals, Bearded Seals, and Walrus near St. Michael and Stebbins, Alaska

By:

Henry P. Huntington
Huntington Consulting
Eagle River, Alaska
hph@alaska.net
Ph: (907) 696-3564

Mark Nelson and Lori T. Quakenbush
Alaska Department of Fish and Game
Fairbanks, Alaska
mark.nelson@alaska.gov, lori.quakenbush@alaska.gov
Ph: (907) 459-7374, (907) 459-7214

Final Report
Approved December 2015

Final report should be cited as:
Huntington, H.P., M. Nelson, and L.T. Quakenbush. 2015. Traditional knowledge regarding ringed seals, bearded seals, and walrus near St. Michael and Stebbins, Alaska. Final report to the Eskimo Walrus Commission, the Ice Seal Committee, and the Bureau of Ocean Energy Management for contract #M13PC00015. 7pp.

Introduction

Ringed seals and bearded seals are important species for subsistence harvests by Iñupiaq and Yup'ik hunters from St. Michael and Stebbins in southern Norton Sound, Alaska. Walrus are found and hunted in this area, too. These marine mammals are iconic Arctic animals, and at risk from climate change. Increasing industrial activity in the Chukchi Sea, coastal development in the Norton Sound region, and shipping through the Bering Strait are additional potential stressors to seal and walrus populations. A satellite telemetry study of the distribution, behavior, and movements of seal and walrus is an important contribution to monitoring the effects of a changing environment and the potential effects from industrial activity. While placing satellite transmitters on seals and walrus provides detailed information about the movements and some behaviors of individual animals, documenting traditional knowledge about seals and walrus, through interviews with residents of coastal communities, provides valuable complementary contemporaneous and historical information about the general patterns of each species.

This report summarizes information gathered from interviews held in St. Michael with hunters and other knowledgeable residents from St. Michael and Stebbins in February 2015. This traditional knowledge project used the same approach that the Native Village of Savoonga used when documenting traditional knowledge about bowhead whales on St. Lawrence Island (Noongwook et al. 2007).

A previous project on traditional knowledge of walrus and ice seals was conducted by Kawerak Inc., in 2010–2013 in communities throughout the Bering Strait region, including St. Michael and Stebbins (Kawerak, Inc., 2013). Except as noted below, the information presented here comes from our February 2015 interviews and not from the Kawerak project. A compilation of results from the two projects may be carried out later, to document changes over time and other aspects of seals and walrus.

Methods

We used the semi-directive interview method, in which the interviewers raise a number of topics with the person being interviewed, but do not rely solely on a formal list of questions (Huntington 1998). Instead, the interview is closer to a discussion or conversation, proceeding in directions determined by the person being interviewed, reflecting his/her knowledge, the associations made between walrus and other parts of the environment, and so on. The interviewers use their list of topics to raise additional points for discussion, but do not curtail discussion of additional topics introduced by the person being interviewed.

In St. Michael, we interviewed eight people in one group. Those interviewed from St. Michael were Charlie Fitka, Harold T. Kobuk, Nick Lupsin, Alexander Niksik Jr., and two others that wished to remain anonymous. Two people from Stebbins were interviewed who wished to remain anonymous. The interview was conducted on February 4, at the St. Michael IRA Council office.

The topics identified by the research team in advance of the interviewers were:

Haulouts on land
Overwintering areas and behavior
Use of lagoons and rivers
Feeding patterns and prey
Differences between ringed and bearded seals
Impacts from climate change
Parts of seals that people eat

The results are presented under different headings, reflecting the actual information collected and the fact that some of the subjects blend together, especially changes seen over time in regard to all of the topics. The interviewers were Henry Huntington and Mark Nelson. Lori Quakenbush is the project leader.

Ringed Seals

During the winter and spring there are many ringed seals near Stebbins and St. Michael. They maintain breathing holes in the shorefast ice and in the drifting pack ice. When it is sunny there will by many ringed seals hauled out on the ice, occasionally they make enough noise that they can be heard from town. Ringed seals start to leave when the ice diminishes in Norton Sound, but there are still a lot around during the herring runs in June. Ringed, bearded, and spotted seals all eat herring when they are spawning and their face and whiskers are sometimes covered with herring eggs when they surface to breath. Seals gain weight quickly during the herring run. Ringed seals also eat tomcod (i.e., saffron cod, *Eleginus gracilis*) and other fish, but also eat small shrimp, which are especially plentiful in the Golsovia area.

Ringed seals start sunning with the increasing light in February and by April have started pupping in their snow dens. The rutting males during this time have black faces, smell like gasoline, and are not hunted or eaten. These rutting males are called *tiigaq*.

Residents of St. Michael and Stebbins eat seal meat, blubber (oil), heart, liver, kidneys, intestines, and the spinal cord of ringed and bearded seals. Some people like to eat aged seal flipper, but this is not common anymore.

Some sick seals were seen in 2011, but not large numbers of them. One sick young ringed seal was seen on the beach in the summer of 2014. It did not flee when approached on a four-wheeler. A sick spotted seal was seen in the fall of 2014.

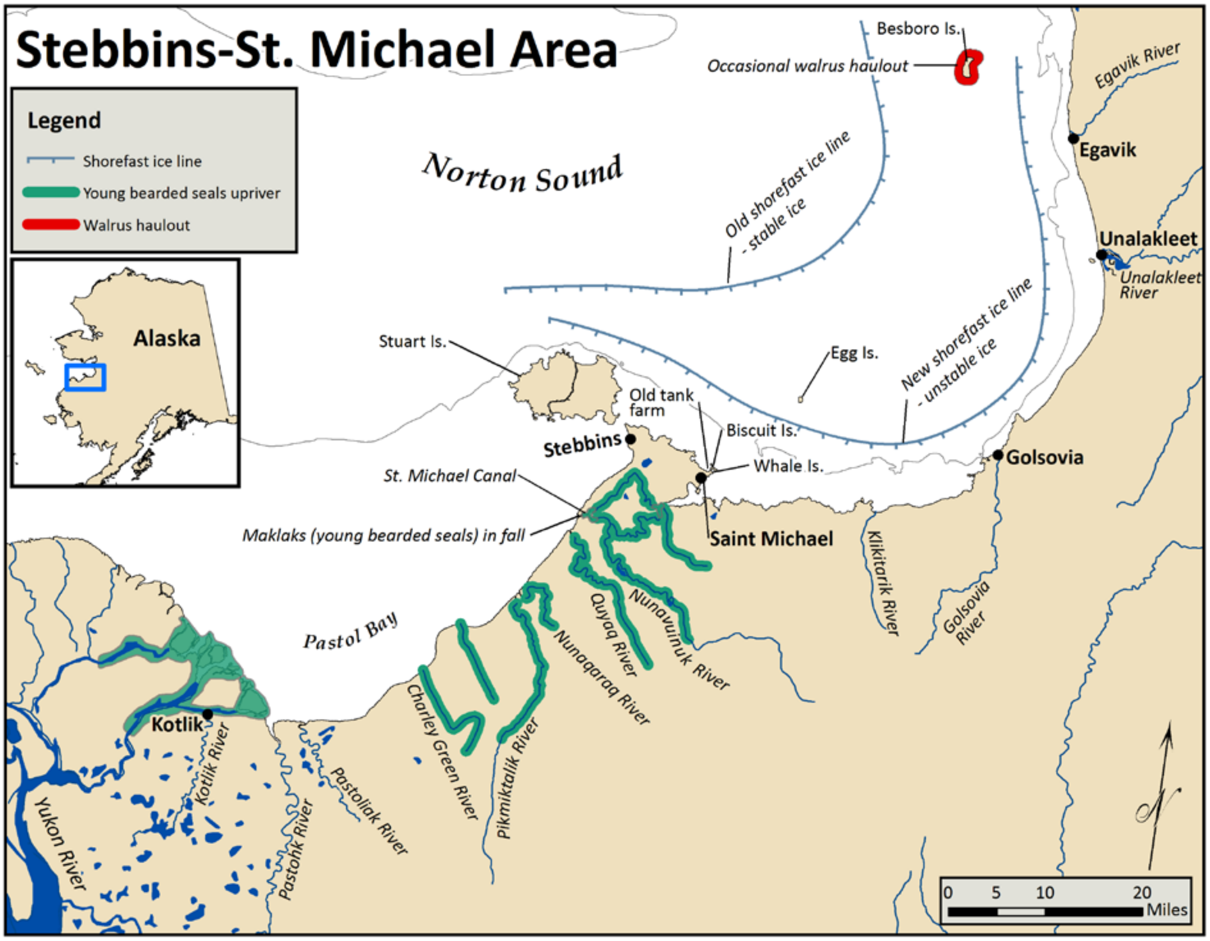

Figure 1. Movements and behavior of ringed seal, bearded seal, spotted seal and walrus near St. Michael and Stebbins as described during traditional knowledge interviews, February 2015.

Bearded Seals

Adult bearded seals are found farther away from the shore in winter and spring, than ringed seals. They are rarely seen near shore hauled out on shorefast ice, but are hunted around Whale Island. Young bearded seals are sometimes found up rivers, including the Yukon as well as smaller rivers around St. Michael and Stebbins. Older bearded seals and ringed seals are not seen in the rivers. Young bearded seals may haul out on riverbanks or mudflats but adult bearded seals are never seen on land.

Ugruq is the term for adult bearded seals; this is an Iñupiaq word. Hunters here also use the Yup'ik word *Omnigaq* for adult bearded seals. The Yup'ik word *maklak* is used for young bearded seals.

The size of adult bearded seals has decreased in recent years. In the past, hunters would see very large bearded seals off of Stuart Island that are believed to have come from farther north. More recently, it is more common to find bearded seals that are a little smaller than these very large ones.

Bearded seals eat whatever they can find farther out in the ocean: shrimp, crab, clams, and fish such as flounder. Some of the shrimp found in bearded seal stomachs are large, the size of a person's hand. When young bearded seals are in rivers they will eat whitefish and tomcod.

One hunter reported harvesting an *ugruq* with a white tissue around its liver. They described it to be white as paper and did not eat the liver, but did eat the meat and blubber.

Walrus

Walrus arrive in spring, hauled out on ice that is carried by currents. They are usually farther from shore, for example near Egg Island. The walrus include bulls, cows, and calves. Walrus are not often seen on land around Stebbins or St. Michael, but occasionally one or two will haul out on Egg Island and Whale Island; one of the small islands just north of St. Michael. One walrus swam up the Yukon River as far as Pilot Station (~120 miles) and stayed in the Pitka's Point (~100 river miles from coast) area for a while. Other walrus have been seen near the mouth of the Yukon and also in St. Michael and Little St. Michael Canal in fall. Even though living walrus are not seen very often, dead walrus wash up on shore regularly in the spring when the currents are just right.

Walrus cows will search for their calves when they become separated. The cows can find the calves when this happens.

St. Michael and Stebbins residents eat the meat, blubber, heart, and liver of walrus. They do not eat the kidney or intestines and only occasionally eat the clams from walrus stomachs.

Sea Ice

The ice has changed greatly and rapidly in the past two decades. In the 1970s and 1980s, the shorefast ice extended out to Egg Island. Hunters could travel over the ice to Egg Island or to Golsovia. Today the shorefast ice extends only a few miles from shore and it is not possible to travel that far on the ice. The ice is also thinner and breaks off more easily, making conditions more dangerous. The ice used to form large pans, but now it is more crumbled up.

In the winter of 2014–15, the ice did not freeze near Stebbins until December. The ice was only a few inches thick when it went out again. There was rain in January, and the ice remains thin and unstable.

Other Information

There are fewer spotted seals in this area since the ice started changing in the 1990s and 2000s. Spotted seals may be seen on the ice near shore during the fall and spring. Steller sea lions, gray whales, and humpback whales are rare occurrences in the area and may scare away other animals when they are around. There used to be puffins in the St. Michael/Stebbins area, but they are no longer seen here.

There used to be commercial beach seiners operating in this area during herring season. They would sometimes snag buoys and anchors belonging to St. Michael and Stebbins residents, which was not popular. This fishery is no longer being conducted.

The water level is higher in summer now than it used to be. There is more algae, and the fish are changing, too. Some St. Michael residents caught what they thought were chum salmon, but which turned out to be another kind of fish. Another fisherman caught two chum salmon a couple years ago, but they smelled like gas when his wife cut their heads off.

The old St. Michael village fuel tank farm may have affected seals. There are fewer seals around there now than there used to be. The tank farm has been moved to the west end of the village now.

Acknowledgements
We appreciate the support of the Eskimo Walrus Commission and the Ice Seal Committee for this project and are grateful to Alexander Niksik Jr. for helping to set up the group interview. The Bureau of Ocean Energy Management (BOEM) funded the work as part of Contract Nos. M09PC00027 and M13PC00015 with the support of Charles Monnett, Catherine Coon, and Dan Holiday. Justin Crawford prepared the maps used during the interviews and the figure in this report.

References

Huntington, H.P. 1998. Observations on the utility of the semi-directive interview for documenting traditional ecological knowledge. Arctic 51(3):237-242.

Kawerak, Inc. 2013. Seal and walrus harvest and habitat areas for nine Bering Strait Region communities. Nome, Alaska: Kawerak, Inc., Social Science Program.

Noongwook, G., the Native Village of Gambell, the Native Village of Savoonga, H.P. Huntington, and J.C. George. 2007. Traditional knowledge of the bowhead whale (*Balaena mysticetus*) around St. Lawrence Island, Alaska. Arctic 60(1):47–54.
