# Supplementary material for: Effects of changing sea ice on marine mammals and subsistence hunters in northern Alaska from traditional knowledge interviews
Source: Biol Lett. 2016 Aug;12(8):20160198. doi: 10.1098/rsbl.2016.0198 (PMC5014017; doi:10.1098/rsbl.2016.0198)
Supplement: ADF&G-KVA 2016 [file rsbl20160198supp8.pdf]

Traditional Knowledge Regarding Ringed Seals, Bearded Seals, Walrus, and Bowhead Whales near Kivalina, Alaska

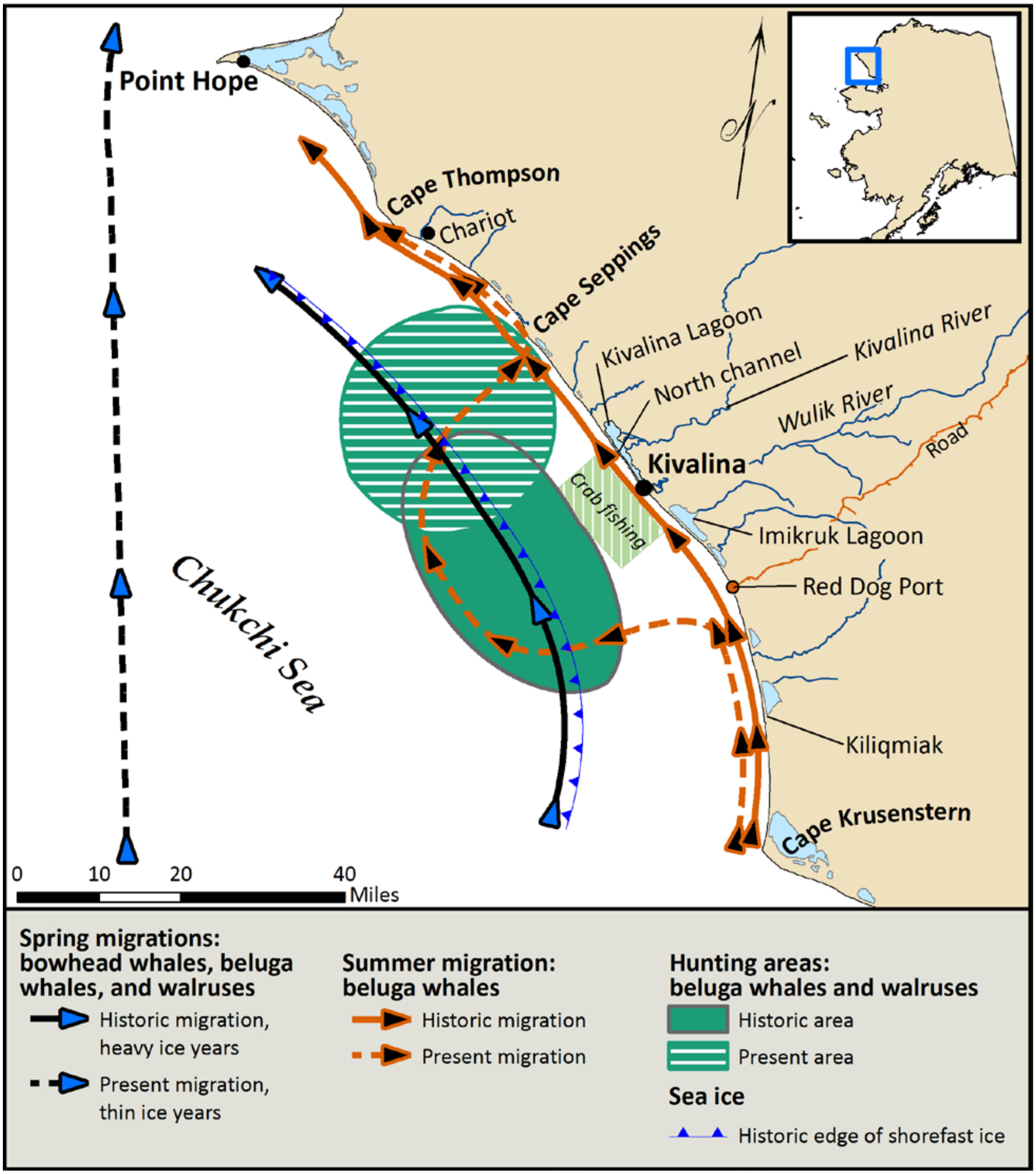

Traditional Knowledge Regarding Ringed Seals, Bearded Seals, Walrus, and Bowhead Whales near Kivalina, Alaska

By:

Henry P. Huntington
Huntington Consulting
Eagle River, Alaska
hph@alaska.net
Ph: (907) 696-3564

Mark Nelson and Lori T. Quakenbush
Alaska Department of Fish and Game
Fairbanks, Alaska
mark.nelson@alaska.gov, lori.quakenbush@alaska.gov
Ph: (907) 459-7374, (907) 459-7214

Final Report
Approved July 2016

Final report should be cited as:
Huntington, H.P., M. Nelson, and L.T. Quakenbush. 2016. Traditional knowledge regarding ringed seals, bearded seals, walrus, and bowhead whales near Kivalina, Alaska. Final report to the Eskimo Walrus Commission, the Ice Seal Committee, and the Bureau of Ocean Energy Management for contract #M13PC00015. 8pp.

Introduction

Ringed and bearded seals are important species for subsistence harvests by Iñupiat hunters from Kivalina (population 384), on the Chukchi Sea coast of northwestern Alaska. Walrus are found and hunted in this area, too. These Arctic marine mammal populations are at potential risk from climate change, increasing industrial activity, coastal development, and shipping through Bering Strait. Scientific studies of distribution, behavior, movements, and habitat use of seals and walrus have made important contributions to understanding the effects of a changing environment and the potential effects from industrial activity. For example, placing satellite transmitters on seals and walrus provides detailed information about the movements and some behaviors of individual animals. Documenting traditional knowledge about seals and walrus, through interviews with residents of coastal communities, however, provides valuable complementary current and historical information about the general patterns of each species.

This report summarizes information gathered from interviews with hunters and other knowledgeable residents in Kivalina, Alaska, in January 2016. This traditional knowledge project used the same approach that the Native Village of Savoonga used when documenting traditional knowledge about bowhead whales on St. Lawrence Island (Noongwook et al. 2007).

Methods

We used the semi-directive interview method, in which the interviewers raise a number of topics with the person being interviewed, but do not rely solely on a formal list of questions (Huntington 1998). Instead, the interview is closer to a discussion or conversation, proceeding in directions determined by the person being interviewed, reflecting his/her knowledge, the associations made between walrus and other parts of the environment, and so on. The interviewers use their list of topics to raise additional points for discussion, but do not curtail discussion of additional topics introduced by the person being interviewed.

In Kivalina, we interviewed five people. All those interviewed wished to remain anonymous. The interviews were conducted on January 7, 2016, at the Kivalina Tribal (IRA) Council office and in the homes of interviewees.

The topics identified by the research team in advance of the interviewers were:

- Haulouts on land
- Overwintering areas and behavior
- Use of lagoons and rivers
- Feeding patterns and prey
- Differences between ringed and bearded seals
- Impacts from climate change
- Parts of seals that people eat

The results are presented under different headings, reflecting the actual information collected and the fact that some of the subjects blend together, especially changes seen over time in regard to all of the topics. The interviewers were Henry Huntington and Mark Nelson. Lori Quakenbush is the project leader.

General Information
Marine mammals continue to be abundant in the Kivalina area. The migratory patterns remain largely the same, with variation in timing from year to year. The big change for hunters is that the ice is no longer a reliable platform for hunting, but is instead a dangerous place that prevents hunters from reaching marine mammals or limits the length of the hunting period.

The number of marine mammals coming past Kivalina has decreased considerably since construction of the Red Dog Mine Port Site in the late 1980s. The noise from that facility deflects marine mammals migrating up the coast, pushing them offshore and out of reach of Kivalina hunters. For example, beluga whales used to be seen every summer, but after the construction of the Port Site, they do not come to Kivalina from the south any more. As a consequence, hunters from Kivalina now often travel northwest of the community towards Cape Thompson to go hunting. It is expensive to travel far to go hunting. Many hunters also go to the area of the north channel into the lagoon, where it is quieter.

A great deal has changed in recent years, but hunters are adjusting to these changes. What used to take place is not what happens now. The availability of high-powered outboards and high-powered rifles has helped hunters adjust in ways that would not have been possible 50 or 100 years ago. A boat trip to Point Hope now takes two hours, instead of all day. Hunters can make day trips from the village instead of having to camp out on the land or ice. People need to be thinking in new ways a lot now.

Kivalina hunters do not pursue marine mammals in fall. They are hunting caribou at that time of year. Not many seals are seen in fall.

Bearded seals, caribou, and fish are the primary subsistence resources that sustain Kivalina. Other species, such as bowhead whales, beluga whales, and walrus are appreciated when they are available, but not essential to the community's well-being.

People eat the blubber (oil) and meat of bearded and ringed seals. They also eat many of the organs of both seals. Elders enjoy small seals of either species. Seals are fat in spring, and their blubber is good for seal oil. Fall seals are not as good for oil. In late spring, male ringed seals are darker and have a different taste "like kerosene" in the words of one hunter. Spotted seal meat is not eaten because it does not taste good, but spotted seal blubber may be used for oil if necessary. There has been no real change in the quality of seal meat or oil.

Sharing of seals and other animals is very important for Kivalina hunters. Hunters will take animals for their families, their relatives, elders, and others in the community. The first animal of the season is typically shared, so many people are excited when a hunter gets his or her first animal, knowing it will be distributed to others. People say that if you give first, more will come, so they do not like to keep their first catch. This practice applies to caribou and to marine mammals.

Shorefast ice does not stay as long as it used to. In some years, it starts to melt as early as March. The ice used to be thicker than it is now, and seems to be getting thinner. This year, in early January, there is open water to the beach. Last winter, 2015, the ice did not stay for good

until March. This winter may be similar. Shorefast ice used to form in early fall and stay until June, but this is no longer the case.

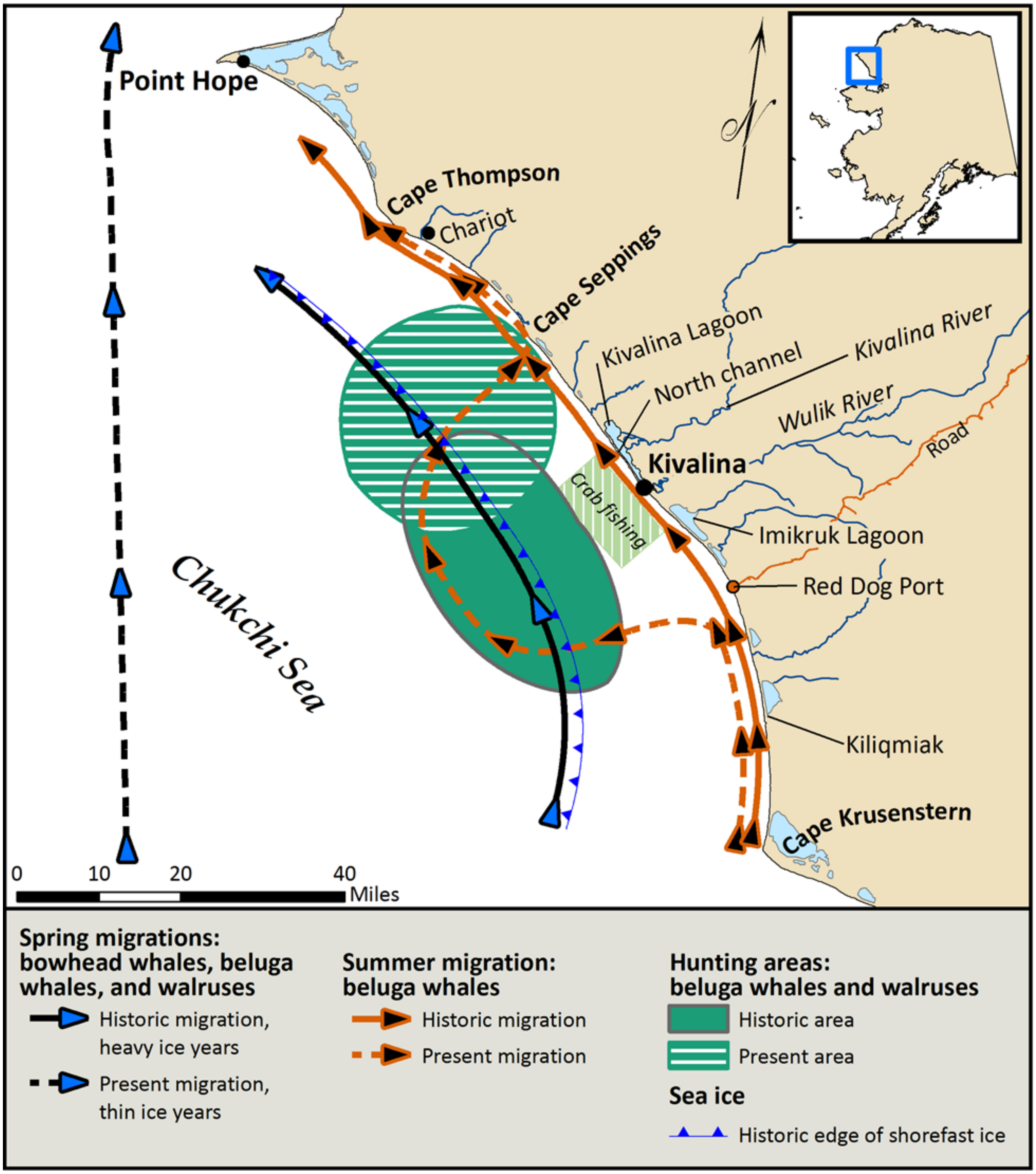

Figure 1. Movements and behavior of bearded seals, spotted seals, ringed seals, walrus, beluga whales, and bowhead whales as described during traditional knowledge interviews, January 2016.

Bearded Seals
Bearded seals start to haul out on the ice in spring when the days get longer. Many seals are seen at this time of year.

They are usually not hunted until the bowhead and beluga hunt is over, so as not to scare off the whales. Bearded seal hunting takes place before it is too warm, so that the meat will not spoil or be affected by insects as it is drying on racks.

In recent years, the thin ice has made it dangerous to go hunting for bearded seals on top of the ice. In 2015, the ice disappeared very quickly after break-up, so the opportunity for hunting bearded seals by boat was very short. In the late 1990s, hunters were caught by surprise one year when the hunting period was only a week long instead of several weeks. After that, they made sure to take advantage of the opportunity as soon as it came. That worked, until last year. In 2015, however, the season was much shorter, again catching hunters by surprise. Offshore winds carried the ice out, but the ice did not come back in after the winds died, as it used to do. This happened once before, in the 1980s. Now hunters fear it will be the new pattern.

A few bearded seals remain in the area after the ice goes out. Bearded seals come through the channel into the lagoon in summer, following fish. Young bearded seals go up rivers in fall. Bearded seals start returning to the area in fall.

A few sick bearded seals have been seen in recent years, with large hairless areas. One young bearded seal taken last October in open water had about half its hair missing. It did not have sores on its skin, but it was skinny. Most seals are healthy, but it used to be very rare to encounter a sick seal.

Ringed Seals
Some ringed seals stay all winter in the Kivalina area. In mid-winter, ringed seals are fat and in their prime. Hunters cannot get them now because the ice is thin or there is open water. Whereas hunting used to start in December or January, it is often not possible to hunt ringed seals until February or March.

It is important to take ringed seal in winter to make bleached sealskin leather, which requires cold weather to cure properly. Bleached ringed sealskin is becoming a rare commodity in Kivalina.

Small, sickly ringed seals have been seen on the beach in the past few years. They do not move off when people approach. They are not common, only a few each year, but this is a new phenomenon. One hunter said the previous time he saw a sick seal was at least 30 years ago, in contrast to seeing at least one each year now.

Spotted Seals
Spotted seals arrive after the ice leaves. Some pups can be seen on the beach all summer, going out to feed and coming back to rest and avoid danger. Spotted seals are seen all summer. Few people hunt spotted seals. Their skins are beautiful and warm, if tanned properly, but the meat is not wanted and the blubber is usually thin. People could get more spotted seals than ringed or

bearded seals these days. Spotted seals do not seem to be affected as much by changes in ice, as they come for the fish rather than the ice.

Walrus

Walrus come northwards past Kivalina in spring when the ice starts to break up, at the end of the bearded seal season. Walrus used to follow a path that brought them past Kivalina, but now seem to go straight from Shishmaref to Point Hope or Cape Thompson, which takes them 50 miles away from Kivalina, too far to go in a boat in broken ice.

Occasionally a walrus will be seen hauled out on the beach. They prefer ice, but will haul out on land if there is no ice.

Walrus are seen going south in fall. A few stray walrus are seen in summer, heading south.

One hunter took a female walrus each of the past two summers, in July, but the blubber was thin and the meat was very dark and red and stinky. They were excited about getting a walrus, because that has become rare for Kivalina hunters, but they could not eat it.

Women are not allowed to go walrus hunting because of stories from long ago of walrus chasing boats and even turning boats over.

Bowhead and Beluga Whales

Bowhead and beluga whales are normally hunted in spring as they migrate north through the leads in the ice. The whales continue to follow this pattern, but with more open water they can take a more direct route from Wales to Point Hope, bypassing Kivalina. When the ice is thick offshore, then bowheads and belugas are more likely to follow the leads along the shore in the Kivalina area. The whales that do come past Kivalina are typically 20 miles offshore. When there was stable shorefast ice in spring, hunters could go 20 miles out and camp at the ice edge to hunt whales. Now, it is too dangerous, so hunters do not have access to the whales. With the thin ice, Kivalina hunters would not be able to pull a large bowhead whale out of the water. There is still an opportunity, but it is shorter than it used to be.

A few belugas will be seen in summer, usually coming from the north. They may be deflected offshore near the Red Dog Port Site, then return to the coast near Cape Seppings. Some come south to feed in the river mouths along the coast, which is why they stay close to shore at this time. Last summer (2015), killer whales kept the belugas in the shallow water close to shore, where hunters could get them. Hunters did not see the killer whales, but heard reports of killer whales from people in Point Hope. The belugas stayed in shallow water, avoiding killer whales even though it made them vulnerable to human hunters. This happens from time to time, but not consistently. Killer whales are doing what they have always done.

Other Information

Gray whales come into the area at the end of summer.

Humpback whales were seen in front of Kivalina in July 2015, a time when there were lots of herring in the area. Humpbacks are seen every few years, usually in July when there are many chum salmon coming up the coast.

There are porpoises (harbor porpoises, *Phocoena phocoena*) in the Kivalina area in summer, but hunters do not pursue them.

Polar bears still come to the Kivalina area, but would usually be encountered by hunters at the ice edge during whaling season. Now that hunters cannot go as far out on the shorefast ice, they see fewer polar bears. Two polar bears have made dens in the hills behind Kivalina this winter.

One hunter found an eight-foot shark on the beach at the end of July one year.

Crabbing is good at Kivalina, especially in fall. The crabs are plentiful in deeper water about five miles from shore.

Some shrimp wash up on the beach after fall storms, along with small fish known as *akaluaq*. Squid and unusual fishes have been washing up on the beach in recent years, which is odd. The squid, which are black and a few inches long, have not been seen before. Some people have also seen swimming worms in the ocean, up to a foot long, the thickness of a pencil. Smaller worms were seen last summer, in a school towards Cape Thompson, at a time when there were also three gray whales nearby. Someone saw a swimming octopus in one recent year.

Acknowledgements
We are grateful for the skill, expertise, and generosity of the five people who participated in the interviews. We appreciate the support of the Eskimo Walrus Commission and the Ice Seal Committee for this project and are grateful to Enoch Adams Jr. for helping to set up the interviews and to Stanley Hawley and the Kivalina IRA Council for use of their office space. The Bureau of Ocean Energy Management (BOEM) funded the work as part of Contract Nos. M09PC00027 and M13PC00015 and we appreciate the support of Charles Monnett, Catherine Coon, Dan Holiday, and Carol Fairfield. Justin Crawford prepared the maps used during the interviews and the figures in this report.

References
Huntington, H.P. 1998. Observations on the utility of the semi-directive interview for documenting traditional ecological knowledge. Arctic 51(3):237-242.

Noongwook, G., the Native Village of Gambell, the Native Village of Savoonga, H.P. Huntington, and J.C. George. 2007. Traditional knowledge of the bowhead whale (*Balaena mysticetus*) around St. Lawrence Island, Alaska. Arctic 60(1):47–54.
