# Supplementary material for: Effects of changing sea ice on marine mammals and subsistence hunters in northern Alaska from traditional knowledge interviews
Source: Biol Lett. 2016 Aug;12(8):20160198. doi: 10.1098/rsbl.2016.0198 (PMC5014017; doi:10.1098/rsbl.2016.0198)
Supplement: ADF&G-OTZ 2016 [file rsbl20160198supp9.pdf]

Traditional Knowledge Regarding Ringed Seals, Bearded Seals, and Walrus near Kotzebue, Alaska

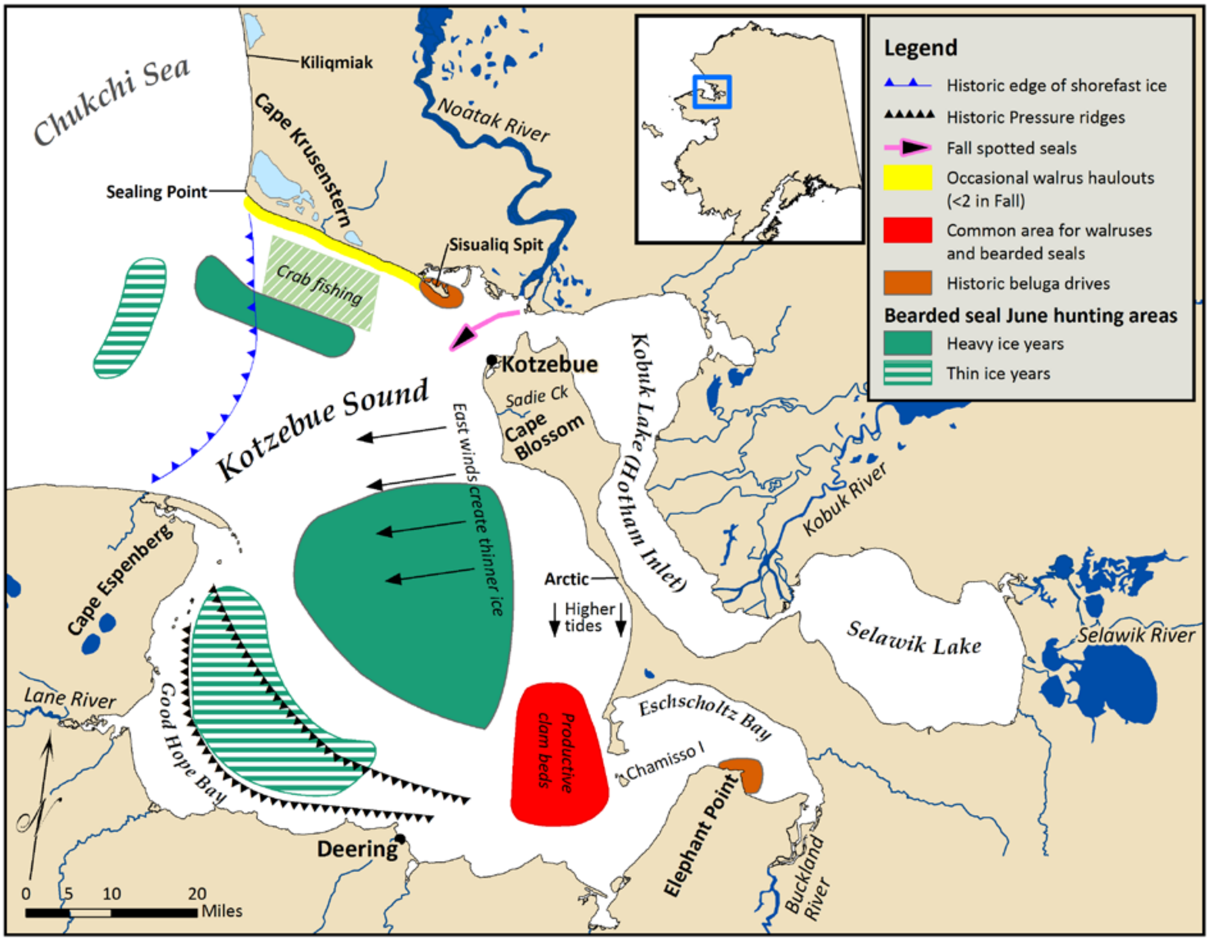

Traditional Knowledge Regarding Ringed Seals, Bearded Seals, and Walrus near Kotzebue, Alaska

By:

Henry P. Huntington
Huntington Consulting
Eagle River, Alaska
hph@alaska.net
Ph: (907) 696-3564

Mark Nelson and Lori T. Quakenbush
Alaska Department of Fish and Game
Fairbanks, Alaska
mark.nelson@alaska.gov, lori.quakenbush@alaska.gov
Ph: (907) 459-7374, (907) 459-7214

Final Report
Approved July 2016

Final report should be cited as:
Huntington, H.P., M. Nelson, and L.T. Quakenbush. 2016. Traditional knowledge regarding ringed seals, bearded seals, and walrus near Kotzebue, Alaska. Final report to the Eskimo Walrus Commission, the Ice Seal Committee, and the Bureau of Ocean Energy Management for contract #M13PC00015. 11pp.

Introduction

Ringed seals and bearded seals are important species for subsistence harvests by Iñupiat hunters from Kotzebue (population 3,284), in northwestern Alaska. Walrus are found and hunted in this area, too. These Arctic marine mammal populations are at potential risk from climate change, increasing industrial activity, coastal development, and shipping through Bering Strait. Scientific studies of distribution, behavior, movements, and habitat use of seals and walrus have made important contributions to understanding the effects of a changing environment and the potential effects from industrial activity. For example, placing satellite transmitters on seals and walrus provides detailed information about the movements and some behaviors of individual animals. Documenting traditional knowledge about seals and walrus, through interviews with residents of coastal communities, however, provides valuable complementary current and historical information about the general patterns of each species.

This report summarizes information gathered from interviews with hunters and other knowledgeable residents in Kotzebue, Alaska, in January 2016. This traditional knowledge project used the same approach that the Native Village of Savoonga used when documenting traditional knowledge about bowhead whales on St. Lawrence Island (Noongwook et al. 2007).

Methods

We used the semi-directive interview method, in which the interviewers raise a number of topics with the person being interviewed, but do not rely solely on a formal list of questions (Huntington 1998). Instead, the interview is closer to a discussion or conversation, proceeding in directions determined by the person being interviewed, reflecting his/her knowledge, the associations made between walrus and other parts of the environment, and so on. The interviewers use their list of topics to raise additional points for discussion, but do not curtail discussion of additional topics introduced by the person being interviewed.

In Kotzebue, we interviewed six people individually. Those interviewed were John Goodwin, Cyrus Harris, Henry (Boyuk) Goodwin, and three others who wished to remain anonymous. The interviews were conducted on January 6 and 7, 2016, in the home of one interviewee, at the Kotzebue IRA Council office, at the Nullagvik Hotel, and, in one case, beginning at the Bering Air terminal, en route to Kivalina, at the Kivalina community center, and concluding at the Kivalina IRA Council office.

The topics identified by the research team in advance of the interviewers were:

- Haulouts on land
- Overwintering areas and behavior
- Use of lagoons and rivers
- Feeding patterns and prey
- Differences between ringed and bearded seals
- Impacts from climate change
- Parts of seals that people eat

The results are presented under different headings, reflecting the actual information collected and the fact that some of the subjects blend together, especially changes seen over time in regard to

all of the topics. The interviewers were Henry Huntington and Mark Nelson. Lori Quakenbush is the project leader.

General Observations about Seals

Hunters on the coast would take seals for their own families and also for families that could not hunt for themselves, such as elders and widows. They would also take seals to trade with people from upriver, getting dried fish and furs in return.

People eat seal oil, meat, heart, kidneys, intestine, and liver of seals. Bearded seals are preferred, though all seals can be eaten. From spotted seals, hunters usually take the hide and blubber, as the meat is not regarded as tasty. Bearded seal flippers would be aged underground and eaten after a few weeks. In the old days, people would store seal and other foods in sealskin pokes in ice cellars. Today, they typically use electric freezers and modern containers.

Elders like young bearded seals, which produce nice, clear oil. Older male bearded seals yield oil that is yellow and less preferred.

By May, seals are making holes in the ice and coming out to lie on top of the ice. Historically, hunters would start hunting when the seals started hauling out atop the ice. Most of the seals seen at this time are adult seals, but hunters would also take young seals when they came out of their lairs. The skins were very desirable at that stage, soft and good for liners. The best hunting for pups was in years with little snow in early spring, which did not happen every year.

Soon after break-up, only bearded and ringed seals are around. Later on, while the ice is still there, spotted seals arrive and the bearded seals and most ringed seals leave. Spotted seals are aggressive and scare off the other seals. Once spotted seals are there, hunters either stop looking for bearded seals or look elsewhere for bearded seals. This is usually in June or July.

There are seals year round in Kotzebue Sound and along the coast. From satellite tagging, hunters now know that the juveniles travel far, even into the Bering Sea, whereas older seals tend to stay closer to Kotzebue Sound.

Hunters can get bearded, ringed, and spotted seals in fall, though the bearded seals are almost all juveniles.

The quality of seal and walrus meat, blubber, other foods, oil and hides has not changed. Seal behavior has not changed, either, despite changes to the ice.

This past year, seals were fat and healthy, and there were many pups, despite the poor ice conditions for hunters. Hundreds if not thousands of seals were seen in Eschscholtz Bay in 2015. Lots of seals were seen between Kotzebue and the Chamisso Islands, including seals sleeping on the water surface. The only big change is the loss of hunting opportunity due to poor ice and rapid disappearance of ice after break-up starts.

When the snow melts early, there is no protection for seal pups from predators such as jaegers and ravens and foxes. The roof of the den collapses and the pup is exposed. Under the snow, seals move around and make escape routes from their dens.

The smallest pups do not go into the water.

There are as many or more seals now as there were in the past. There has never been a shortage of seals compared with people's needs. There is not as much hunting, largely because there are fewer dogs to feed. Hunters also used to get seals to make sealskin pokes for storing oil and meat, but today there are other containers so no need to hunt seals for this purpose.

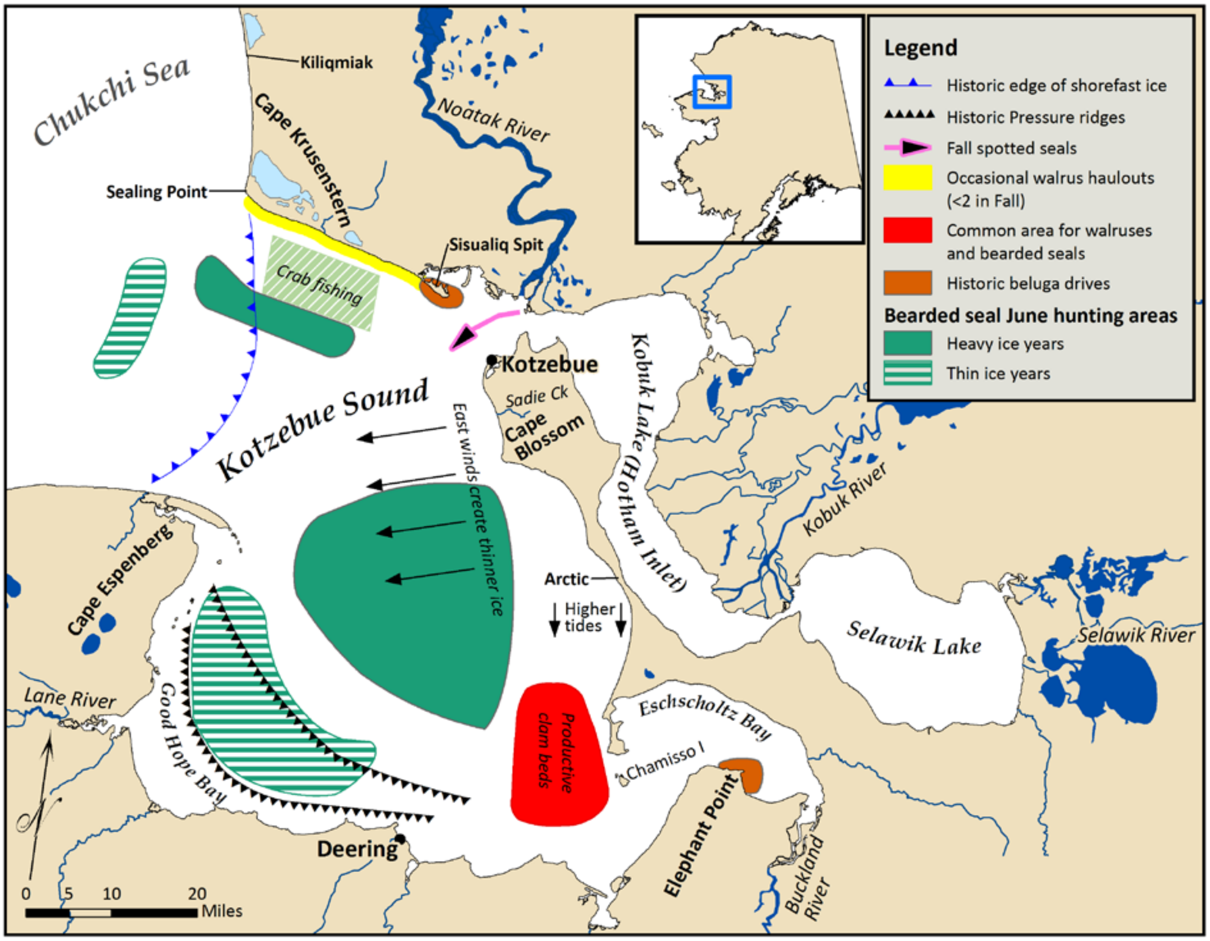

Figure 1. Movements and behavior of bearded seals, spotted seals, ringed seals, walrus, and beluga whales as described during traditional knowledge interviews, January 2016.

Seals and Disease

It seems there may even be an overpopulation of seals, which could cause starvation if there is not enough food for all the seals. When animals starve, they can develop many different kinds of disease, which may help explain the skin sores and other problems hunters saw a few years ago.

Hair loss is normal in seals, and can be seen on bearded, ringed, and spotted seals. When seals were taken, hunters' wives would pull on the hair to see if the hair was firmly attached or if the seal was in the middle of the molt. Seals that are molting or have lost hair are good for making leather, for ropes and mukluk soles and other purposes. Hairless seals would be fed to dogs, or if they seemed fat and healthy otherwise would be eaten by people. One hunter took a hairless ringed seal in winter, and his grandmother said he should burn it, so he did. People would say the seals did not spend enough time on top of the ice to molt fully. The hair loss may also have come from rubbing against the seabed. The loss of ice in recent years may mean the seals cannot spend as much time hauled out as they used to, which could affect the molt. Seals may have to learn to adapt, for example by hauling out on land instead of ice.

The recent hair loss, accompanied by skin sores and other signs of ill health, is a new phenomenon. Hunters do not want to handle, much less eat, seals that show signs of poor health. In addition to the sores, the seals appear lethargic. Ringed seals hauled out on the beach do not flee, so would be easy to catch, but hunters do not want to approach them once they see they are sick. This illness occurred first in 2011, but seems mostly to have disappeared. Some hunters see it as nature's way of dealing with overpopulation of seals.

Bearded Seals

In spring, bearded seals are the focus for hunting, getting food for spring and for the following winter. When the ice started to break up, hunters would go to Sealing Point (near Cape Krusenstern) to hunt bearded seals. In the days before outboard motors, hunters would use kayaks to go between floes and in the cracks in the ice to pursue bearded seals.

The ice edge off Cape Krusenstern is a good place to hunt bearded seals, though the seals move around. Many may be seen one day, and none the next day, in the same spot. The waters about 10 miles west of Cape Blossom are also rich with seals, but this is farther from shore and more dangerous to travel to. The waters north of Cape Krusenstern are also good for hunting, at Kiliqmiak, just south of Rabbit Creek. There are many creeks and other places on either side of Cape Krusenstern where hunters can find refuge in case of bad weather. There are fewer places to find refuge in southern Kotzebue Sound.

It used to be that there were two or three weeks of good hunting conditions for bearded seals, as the ice broke up but before it was gone entirely. In spring 2015, there was only a week or less, because the ice disappeared very quickly after break-up. This is due in part to more east wind, blowing the ice out, and in part to thinner ice during the winter, making it easier to melt and move. The lack of ice also meant that waves could build up more in Kotzebue Sound, increasing the risk for hunters. Some ice remained towards Goodhope Bay, but it was dangerous to go that far in open water. Still, if hunters can find an ice floe, there are often bearded seals nearby, so the hunting can be good. With so little ice, the bearded seals have few options left, so are concentrated near the floes that remain. The risk of exposure to wind and waves is still higher for hunters with so little ice.

Bearded seals come in earlier than they used to, but often stay on thin ice where hunters cannot reach them. Thinner ice also makes it harder to hunt for bearded seals, as travel on top of the ice is more dangerous for hunters. Bearded seals can be hunted while they are swimming in open

water, but hunters prefer to get them on the ice since hauling them in and out of the boat is difficult. The adult bearded seals come in earlier because the ice breaks up earlier, but they do not stay as long because the ice goes away quickly, leaving only swimming seals.

Bearded seals need white ice (thicker ice), but there is more and more black ice (thinner, younger ice) in Kotzebue Sound these days, which produces fewer pressure ridges and thus less denning habitat. The ice is no longer suitable for camping during the spring hunt. The ice that is left moves very quickly in the currents and can break up quickly, making it dangerous for camping. Adult bearded seals in spring have thinner blubber than they did in the past, only an inch or an inch-and-a-half thick as opposed to three to four inches.

Yearling bearded seals (*ugruchiaq*) return in September, before the ice starts to form. Many of these seals spend time up rivers, including the Ugrugvik Lakes, just north of the mouth of the Kobuk River. A bearded seal was seen close to Ambler on the Kobuk River in September 2015. There are often bearded and ringed seals up the Kobuk, and seemed to be even more this past fall (2015), well over a dozen. Some bearded and ringed seals also go up the Noatak River. There were more yearling bearded seals last fall (2015) than ever before. They appeared very healthy.

One hunter has checked the stomachs of three bearded seals in his lifetime. All were full of shrimp. The seals will feed throughout Kotzebue Sound, but a prime feeding area is off the Chamisso Islands.

Ringed Seals

Some ringed seals will stay in Kotzebue Sound during summer, but most move away because they do not like to be around spotted seals. Ringed seals return in late summer. The juveniles come first, and the adults later in fall after the spotted seals have started to leave. Adult ringed seals will stay in Kotzebue Sound all winter. They make their dens in the pressure ridges. Large ridges used to form in the middle of Kotzebue Sound, but the ice today is thinner. Merging currents at Cape Blossom also created pressure ridges close to shore, but today this area is often open water even into winter, reducing denning habitat for ringed seals. In mid-winter, ringed seals are fat and healthy, at their most prime condition. They float very well at this time of year.

Spotted Seals

After the ice is gone in summer, spotted seals are the ones seen in the area until fall. They come to feed on the fish in Kotzebue Sound and in the rivers. Spotted seals are molting when they arrive. That is the time of year when everything is molting. Many spotted seals go up the Noatak River, to just below the hatchery, though they are generally in the lower part of the river. They also go into Hotham Inlet, Kobuk Lake, and Selawik Lake. They feed on fish in the freshwater areas.

After the ice floes start to form in fall, spotted seals will haul out in the hundreds and ride the ice to the southwest when the wind blows from the northeast. In fall 2015, thousands of spotted seals were seen in front of Kotzebue. Buckland hunters took many spotted seals in Eschscholtz Bay, including by the mouth of the Buckland River.

Spotted seals may be arriving a little later than they used to, and are staying a lot longer in fall.

Walrus
Walrus come into Kotzebue Sound in spring, and feed in the waters off the Chamisso Islands in southern Kotzebue Sound. They may have young there, too. They stay while there is still ice in the area.

In fall, one or two walrus may haul out on the beach to the east of Cape Krusenstern. There are never many that do this.

When there are walrus around, seals will not be seen. The seals stay away from walrus.

A walrus taken last summer had shrimp in its stomach.

Beluga Whales
Beluga whales will come into Kotzebue Sound when the ice starts to break up, coming in the cracks that form from Cape Espenberg and Cape Krusenstern.

There has been a huge change in beluga whales in Kotzebue Sound. It is not even clear that there is a Kotzebue Sound population any more. People used to get them every year. Sisualiq is named for beluga whales (*sisuaq*), and there used to be drive hunts there every summer. The drive hunt used to be well organized and coordinated, but now people tend to go for themselves rather than as a group. Belugas do not go into Eschscholtz Bay the way they used to, either. The few sightings in recent years have been around the mouth of the Noatak River, with a few in Eschscholtz Bay, and one juvenile beluga as far upriver as Selawik Lake. One beluga was seen in the shallows between Kotzebue and Sisualiq, in only a few feet of water, during the tomcod run in October, which is very late for a beluga to be seen near Kotzebue.

There is a lot of boat traffic these days, especially hunters from Kotzebue going after bearded seals towards Cape Krusenstern or putting in crab pots north of Kotzebue. The noise may deter belugas from coming into the Sound. Elders said the belugas came in because it was quiet. When air traffic increased at Kotzebue, the belugas started to decline. The noise of jet planes can be heard even at Sisualiq. The conflict between Buckland and Kotzebue hunters over hunting in Eschscholtz Bay (Elephant Point), which was the last place belugas were plentiful in Kotzebue Sound, may also have contributed to the decline. The custom is to let the first animals pass, but these days there are no animals to follow the first ones. Today, though, hunters may pursue the first animals. Hunters no longer coordinate the hunt the way they used to. Most beluga hunting today is with nets.

The ice entrapment of belugas in Russia in the mid-1980s seems the most likely explanation for their disappearance. There was a lot of harvest prior to that, but it seems hard to believe that overharvest is the explanation for the nearly complete disappearance of belugas from Kotzebue Sound.

The belugas that came into Kotzebue Sound in large numbers in one year in the 1990s were thinner than the belugas Kotzebue hunters are used to taking.

A group of belugas came to the Sadie Creek area a few years ago. Hunters think killer whales chased them in, because the belugas nearly beached themselves in the shallows.

One year, hunters found a king salmon in the stomach of a beluga whale. Two other belugas taken at the same time had only tomcod in their stomachs. A beluga taken in summer 2015 had a stomach full of crabs. Crabs are sometimes seen in beluga stomachs, but usually the stomachs are mostly full of fish.

Killer whales
There are more killer whales than there used to be in the Kotzebue Sound area. This is known from observations and also from the results of acoustic monitoring done by the Kotzebue IRA Council. When they follow belugas, the belugas will stay very close to shore and even go into very shallow water. Killer whales stay where the water is deep. One hunter saw a killer whale kill a large male beluga by holding it under water until it died. After that, the killer whales tore the beluga apart.

Other Information
Fewer ribbon seals are seen now than in the past. Hunters used to encounter them now and then, but hardly seem them now. People never ate them, but took the hides and fed the meat and blubber to dogs. One hunter saw many ribbon seals a few years ago, between Point Hope and Cape Lisburne.

A fur seal was once seen in Kotzebue Sound, many years ago.

The waters between Cape Espenberg and Cape Krusenstern are very dynamic, with open water and moving ice in winter and spring, and an abundance of marine mammals.

The southern end of Kotzebue Sound, including Eschscholtz Bay, has tides of about four feet. Boats hauled up at Cape Espenberg can be left high and dry at low tide if hunters are not paying attention. The northern end, including Kotzebue, does not.

The water level in northern Kotzebue Sound, Hotham Inlet, Kobuk Lake, and Selawik Lake is controlled by wind. North and east winds cause the water level to drop; west and south winds cause it to rise, with the highest water coming from south winds. Fish movements are determined by the currents caused by the wind. There used to be little south wind in summer, but September would bring south winds, causing the water to rise. The prevailing wind in winter is from the east, lowering the water and preventing flooding. Today, there is more flooding due to changing wind patterns.

An east wind opens the ice to the west of a line between Cape Espenberg and Cape Krusenstern. In the old days, hunters would travel to the ice edge by dog team when there was an east wind so they could hunt seals there. They would wait for the wind to calm down, so that the risk of being blown out to sea was less, and then have a day or two of seal hunting before the open water froze over again. Today, east winds may open the ice may well within Kotzebue Sound. While new ice may form on the open water, the ice still remains thin and does not have time to become thick. It seems that the east winds are stronger than they used to be.

In those days, the ice in Kotzebue Sound was five to six feet thick, and there was no moving ice inside of the Espenberg-Krusenstern line. There were more pressure ridges in Kotzebue Sound, including very large ones in the middle of the sound. Seals make their lairs in the pressure ridges, where the ice and snow provide good habitat. Today, the ice is thinner and flatter, though there are still some smaller pressure ridges, closer to shore, so there is still denning habitat for seals. The thinner ice is more dangerous for traveling, as it can open up or form cracks well into Kotzebue Sound. No longer can hunters travel straight from Kotzebue to Cape Espenberg—the ice is too unreliable. There is much less shorefast ice, since there are not large pressure ridges to hold the ice in place. There are fewer strong west winds to push the ice into Kotzebue Sound and build up those pressure ridges, and the ice is thinner. Hunters used to be able to camp on the ice past Cape Krusenstern, but today the ice is not reliable.

While ice and weather conditions have always varied from year to year, the changes have really taken effect over the past fifteen years or so. In the 1980s, there was still ice for hunting bearded seals into July, but now the ice is gone in June.

When the ice goes out in spring, Kotzebue Sound opens up a week or two after Kobuk and Selawik Lakes. The elders have always said it is dangerous to go out while there is still ice in Kobuk Lake and Hotham Inlet. In some years, the ice goes out quickly, and in other years it goes back and forth in Kotzebue Sound for some weeks.

A strong current carries spring pack ice from Cape Espenberg northwards towards Cape Krusenstern. A current along the coast from Shishmaref merges with a current coming from Goodhope Bay and southern Kotzebue Sound to produce a stronger current going north. This current does not go into northern Kotzebue Sound, but goes north past Cape Krusenstern.

There used to be more snow, on the ice and in town.

The weather used to be easier to predict. Now it is hard to read. Clouds forming on the tops of mountains are a good indicator that winds are coming, as are the ways high clouds form or disappear in the sky.

Hunters use satellite imagery of sea ice to plan boat travel in Kotzebue Sound, to help improve safety and efficiency as they will know where to find ice for hunting bearded seals.

Concerns

Continued climate change, and subsequent changes, like more commercial shipping, remain a concern. If the ship traffic starts to occur when marine mammals are migrating, it could be a major conflict or impact. The Arctic Waterways Safety Committee is a good forum for discussing shipping. Having a shipping lane from Bering Strait to Canada would be a good way to reduce impacts and risks to hunters.

The reports of dying murres and poor salmon returns from around the state raise concern about how much the ocean is changing and what that is likely to mean for people in Kotzebue, even if seals in Kotzebue Sound appear to be doing well so far.

Acknowledgements

We are grateful for the skill, expertise, and generosity of the six hunters who participated in the interviews. We appreciate the support of the Eskimo Walrus Commission and the Ice Seal Committee for this project and are grateful to John Goodwin and Alex Whiting for helping to set up the interviews and to the Kotzebue IRA Council for providing space for interviews. The Bureau of Ocean Energy Management (BOEM) funded the work as part of Contract Nos. M09PC00027 and M13PC00015 and we appreciate the support of Charles Monnett, Catherine Coon, Dan Holiday, and Carol Fairfield. Justin Crawford prepared the maps used during the interviews and the figure in this report.

References

Huntington, H.P. 1998. Observations on the utility of the semi-directive interview for documenting traditional ecological knowledge. Arctic 51(3):237-242.

Noongwook, G., the Native Village of Gambell, the Native Village of Savoonga, H.P. Huntington, and J.C. George. 2007. Traditional knowledge of the bowhead whale (*Balaena mysticetus*) around St. Lawrence Island, Alaska. Arctic 60(1):47–54.
