# Supplementary material for: Effects of changing sea ice on marine mammals and subsistence hunters in northern Alaska from traditional knowledge interviews
Source: Biol Lett. 2016 Aug;12(8):20160198. doi: 10.1098/rsbl.2016.0198 (PMC5014017; doi:10.1098/rsbl.2016.0198)
Supplement: ADF&G-SHH 2016 [file rsbl20160198supp10.pdf]

Traditional Knowledge Regarding Ringed Seals, Bearded Seals, and Walrus near Shishmaref, Alaska

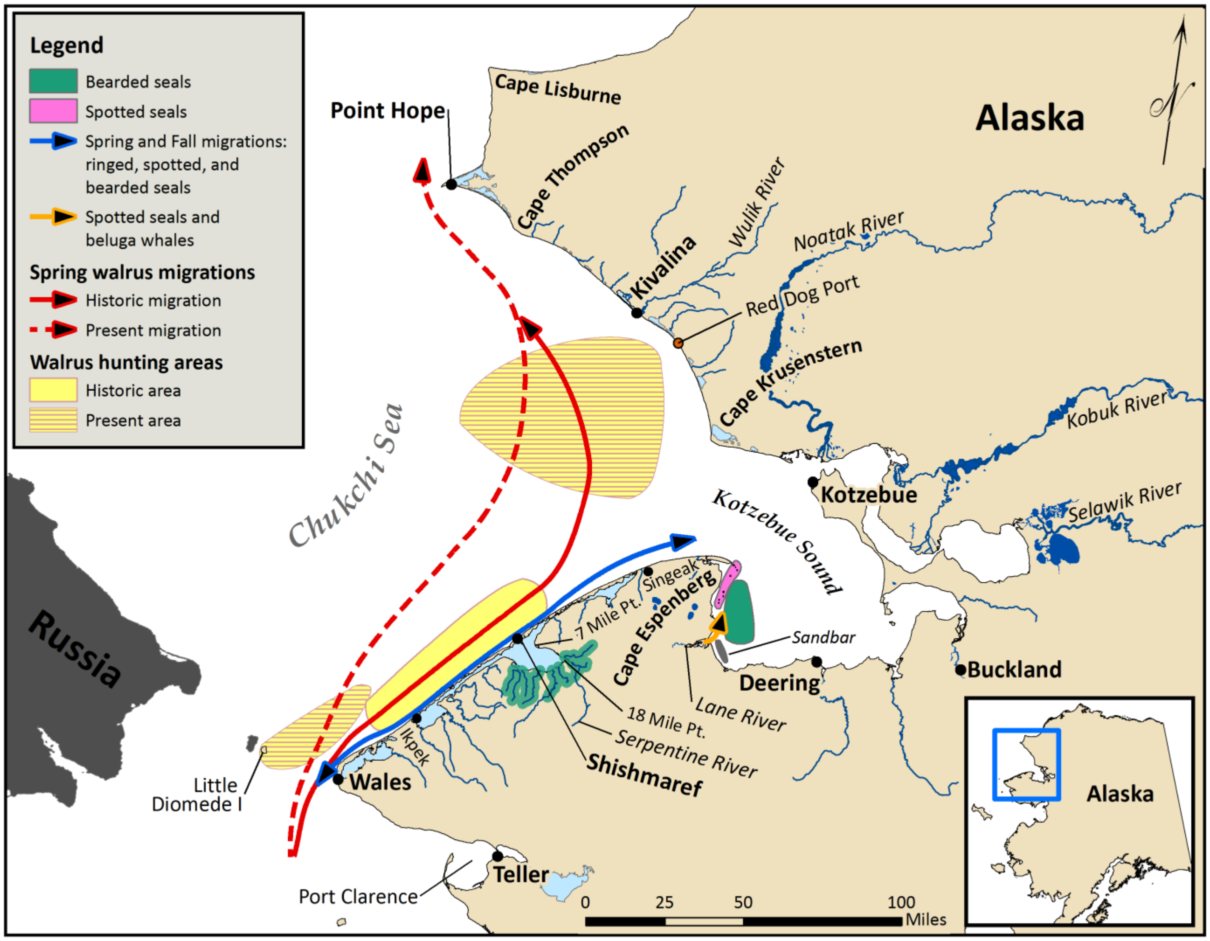

Traditional Knowledge Regarding Ringed Seals, Bearded Seals, and Walrus near Shishmaref, Alaska

By:

Henry P. Huntington
Huntington Consulting
Eagle River, Alaska
hph@alaska.net
Ph: (907) 696-3564

Mark Nelson and Lori T. Quakenbush
Alaska Department of Fish and Game
Fairbanks, Alaska
mark.nelson@alaska.gov, lori.quakenbush@alaska.gov
Ph: (907) 459-7374, (907) 459-7214

Final Report
Approved July 2016

Final report should be cited as:
Huntington, H.P., M. Nelson, and L.T. Quakenbush. 2016. Traditional knowledge regarding ringed seals, bearded seals, and walrus near Shishmaref, Alaska. Final report to the Eskimo Walrus Commission, the Ice Seal Committee, and the Bureau of Ocean Energy Management for contract #M13PC00015. 9pp.

Introduction

Bearded seals, spotted seals, and ringed seals are important species for subsistence harvests by Iñupiat hunters from Shishmaref (population 579), on the northern coast of the Seward Peninsula, in the Chukchi Sea coast, just north of Bering Strait in Alaska. Walrus are found and hunted in this area, too. These Arctic marine mammal populations are at potential risk from climate change, increasing industrial activity, coastal development, and shipping through Bering. Scientific studies of distribution, behavior, movements, and habitat use of seals and walrus have made important contributions to understanding the effects of a changing environment and the potential effects from industrial activity. For example, placing satellite transmitters on seals and walrus provides detailed information about the movements and some behaviors of individual animals. Documenting traditional knowledge about seals and walrus, through interviews with residents of coastal communities, however, provides valuable complementary current and historical information about the general patterns of each species.

This report summarizes information gathered from interviews with hunters and other knowledgeable residents in Shishmaref, Alaska, in January 2016. This traditional knowledge project used the same approach that the Native Village of Savoonga used when documenting traditional knowledge about bowhead whales on St. Lawrence Island (Noongwook et al. 2007).

Methods

We used the semi-directive interview method, in which the interviewers raise a number of topics with the person being interviewed, but do not rely solely on a formal list of questions (Huntington 1998). Instead, the interview is closer to a discussion or conversation, proceeding in directions determined by the person being interviewed, reflecting his/her knowledge, the associations made between walrus and other parts of the environment, and so on. The interviewers use their list of topics to raise additional points for discussion, but do not curtail discussion of additional topics introduced by the person being interviewed.

In Shishmaref, we interviewed five people, three in one group and two individually. Those interviewed were William Olanna, Bert Iyatunguk, Fred Weyiouanna, Morris Kiyutelluk, and one other who wished to remain anonymous. The interviews were conducted on January 4 and 5, in the homes of the interviewees and at the Shishmaref IRA Council office.

The topics identified by the research team in advance of the interviewers were:

Haulouts on land
Overwintering areas and behavior
Use of lagoons and rivers
Feeding patterns and prey
Differences between ringed and bearded seals
Impacts from climate change
Parts of seals that people eat

The results are presented under different headings, reflecting the actual information collected and the fact that some of the subjects blend together, especially changes seen over time in regard to

all of the topics. The interviewers were Henry Huntington and Mark Nelson. Lori Quakenbush is the project leader.

General Observations

In spring, bearded seals come first, followed by walrus, and then spotted seals. When the spotted seals are plentiful near Shishmaref, hunters know the bearded seal season is over, unless they go most of the way to Kotzebue to catch up to them. Today, the changing climate is shortening the time separating the migrations and they are blending together such that bearded seals and walrus may arrive at the same time.

Animals are arriving earlier in spring than they used to, and the spring season is over sooner. It used to be that duck hunting came before bearded seal hunting, but now bearded seal hunting takes place at the same time as duck hunting. Eggs and berries also come earlier than they used to.

Seals are not common around Shishmaref in summer, from about July to September. They are at Cape Espenberg and in Kotzebue Sound.

In fall, there are more seals in the Shishmaref area than there used to be.

Sea ice breaks up earlier than it used to and freezes much later. This winter (2015-16), there was open water on the ocean until Christmas, when it usually freezes by November. The ice is thin and dangerous much of the time, not solid and reliable as it once was in winter. There are no large pressure ridges to hold the ice in place, so in spring the ice will break up quickly and be dangerous to travel on.

Seals feed on herring and salmon, which are plentiful in the Shishmaref area.

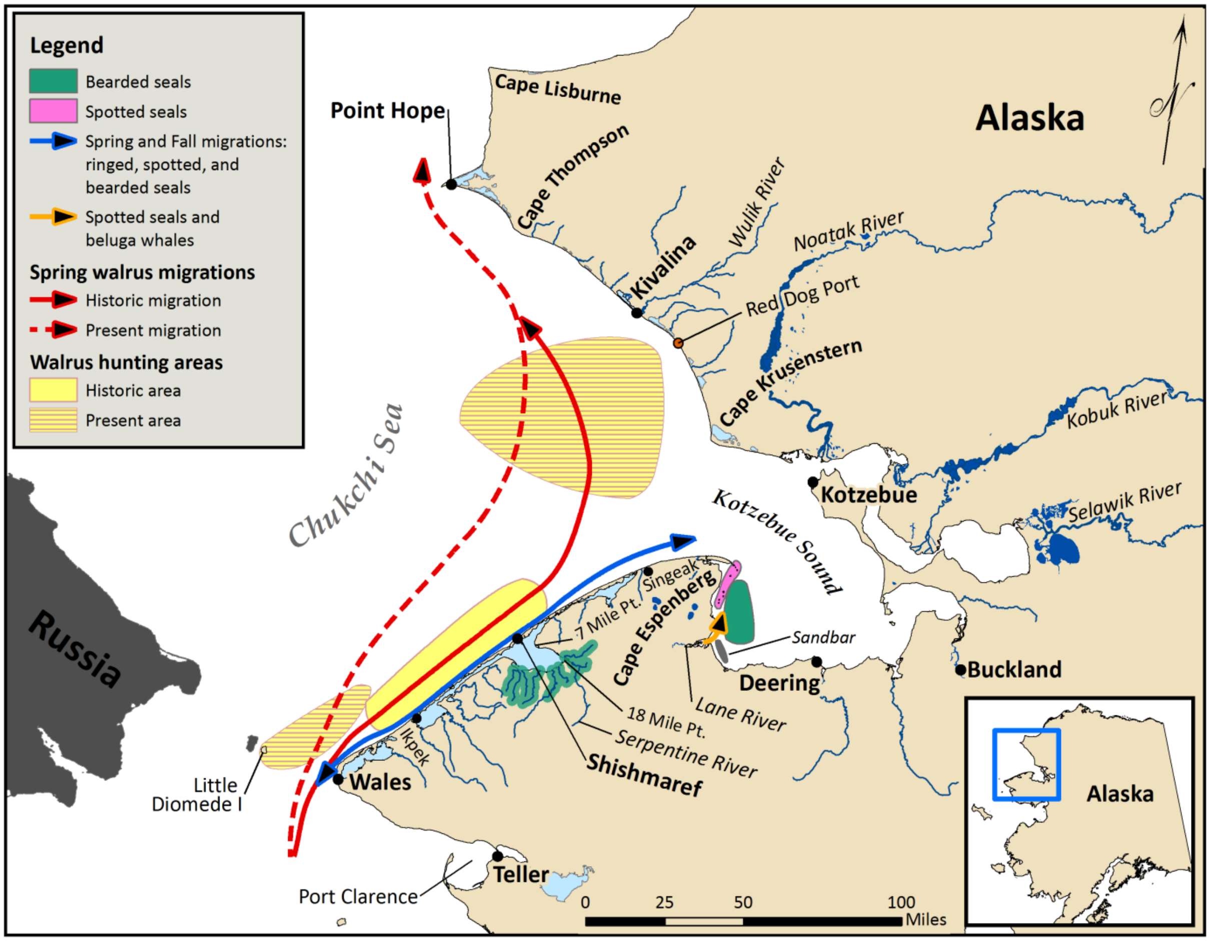

Figure 1. Movements and behavior of bearded seals, spotted seals, ringed seals, walrus, and beluga whales as described during traditional knowledge interviews, January 2016.

Bearded Seals

Bearded seals are the main source of food for Shishmaref residents. Bearded seal oil and meat are a typical winter meal. Bearded seals are hunted mainly in spring, from the shorefast ice or in the pack ice when boating is possible.

In spring, bearded seals migrate closer to the shore and the village than they used to. Hunters do not have to travel as far, unless they are held on shore because the sea ice is piled up against the land. In those years, hunters may have to travel very far, sometimes to northern Kotzebue Sound, in pursuit of bearded seals.

Young bearded seals (unmiaq) migrate north with the adults, but migrate back south slightly ahead of the adults. Unmiaq’s are also found up rivers in summer, especially the Serpentine River and occasionally in smaller rivers and tributaries. They are likely feeding on salmon.

Bearded seals return to the Shishmaref area in late fall, when slush ice starts to form on the ocean. In spring, the bearded seals surface often as they migrate past the village. In fall, they travel differently, surfacing infrequently and moving fast.

One fall during the slush ice period, one hunter saw one pan of sea ice full of spotted seals and another pan nearby that was full of bearded seals.

In recent years, since the Fukushima nuclear reactor breach in 2011, hunters have seen many diseased animals, with sores around their flippers and back end, with white livers, and with bald spots or even with no hair at all. The hair can feel like sandpaper instead of being smooth, and in fall it may come out easily when it should be firmly attached to the skin. In 2015, there were more sick seals than in 2014.

Hunters once found a worm in a bearded seal's liver, most likely a liver fluke. The animal appeared healthy otherwise, though its blubber was yellow-orange.

In spring 2015, after the sea ice broke up, hunters caught a young bearded seals (*unmiaq*) but found that it was covered with white spots. This was something different from the hair loss hunters were familiar with. Hunters do not like to eat diseased animals. One hunter's grandmother told him not to eat seals that have no hair where they are supposed to have hair. Many hunters do not even want to touch animals that appear severely ill.

Bearded seals in fall also showed signs of disease. Diseased seals of all species are typically thin, with little blubber, and do not dive right away or stay down for long. One seal did not dive until three shots were fired at it. Healthy seals dive right away and can stay down for a long time.

People in Shishmaref eat the oil, meat, liver, intestines, kidneys, heart, and lungs of bearded seals. The blubber is rendered into oil. Meat is dried and stored in oil. Liver is cooked and then stored in oil. Intestines are dried, cooked, and stored in oil. Lungs are half-dried and then eaten. They can become hard as wood if left too long. Flippers are hung up with the drying meat until they become tasty. In the old days, seal oil and meat would be stored in sealskin pokes, in shallow holes in the ground, covered with wood. In fall, the ground would freeze and water in the hole would freeze, helping protect the pokes from bears and foxes. The quality of seal meat, seal oil, and seal hides does not appear to have changed.

Some bearded seals have claw marks, probably from polar bears as the seals are hunted in spring.

Albino bearded seals are seen, though rarely.

Spotted Seals

Spotted seals are typically hunted in fall when they return to the Shishmaref area from Cape Espenberg and Kotzebue Sound. They are the first seals seen in early fall before the ice starts to form.

Spotted seals haul out in large numbers on small islands south of Cape Espenberg on the Kotzebue Sound side. They are also seen in large numbers in the mouth of the Lane River, farther south of Cape Espenberg on the same side, particularly in the deep water channel on the north side of the Lane estuary.

In fall 2015, hundreds of spotted seals were seen on top of ice in Shishmaref Lagoon near the mouth of the Serpentine River. During open water, spotted seals are abundant near the entrances to Shishmaref Lagoon likely eating fish because the fishing is poor. The fish arrive in higher numbers once the ice forms and the seals leave.

Spotted seals in recent years have been larger than they used to be. The spotted seals found near Cape Espenberg, at Singeak, and at Ikpek (southwest of Shishmaref) are all big. The spotted seals in Shishmaref Lagoon are only 4-5 feet long, not the big ones.

Spotted seals also suffer from the disease that afflicts bearded seals, the one that causes hair loss.

Ringed Seals

Ringed seals, referred to as common seals, are typically hunted in fall, but can be hunted year round if they are available.

Ringed seals return to the Shishmaref area in late fall, like bearded seals, when slush ice starts to form on the ocean.

The harvest of ringed seals has declined due to the disease that has been seen on so many bearded, spotted, and ringed seals in the past five or so years. People do not want to hunt animals that may be sick. Some ringed seals and ringed seal pups are seen on the beach in summer, but people do not want to harvest them or touch them or even feed them to dogs because of the risk of disease.

Walrus

Walrus are hunted in spring as they migrate northwards with the retreating ice. Walrus migrate farther off shore now because the pack ice is less dense and more broken than in the past.

Walrus are occasionally seen in fall, as lone animals swimming past. One was seen in Shishmaref Lagoon. Some individuals are seen hauled out on the beach, by themselves.

Walrus can be seen sometimes on very small ice floes, instead of in larger groups on larger ice pans the way they used to haul out. Sometimes only the small floes are available.

In the 1970s and early 1980s, some hunters went over 50 miles offshore on the ice by snowmachine to hunt walrus. In those days, the weather was cold and the sea ice was solid.

Today, walrus are sometimes hunted from the ice edge in May. They used to be hunted along the shore north and south of Shishmaref. In some years, hunters have to go as far as Kotzebue Sound and even close to Kivalina to pursue walrus as they migrate northwards. When they do

so, hunters may go to Cape Espenberg and wait for good weather for traveling across Kotzebue Sound. Hunters have also gone as far south as Wales and Diomede to pursue animals.

Walrus appear healthy, with no sign of the disease afflicting seals. Some walrus are skinny, perhaps due to retreating sea ice and having to swim farther from their resting places atop the ice to their feeding areas.

Other Information

Ribbon seals are very skittish, diving off ice floes at the slightest sound or smell of people.

It is harder to find polar bears than it was 20 years ago. They are typically seen in spring when they go out with the sea ice. Nowadays, some get stranded on land. Some are seen swimming straight out to sea, presumably in search of sea ice. Hunters in Shishmaref do not believe the polar bears survive this attempt. Polar bears are seen more often inland than they used to be, usually in early spring before the ice goes out.

Steller sea lions are occasionally seen in the Shishmaref area. This is a relatively new phenomenon, but is still rare.

The Lane River area is a good place to hunt belugas in late summer and fall.

In spring 2015, killer whales kept a gray whale cow and calf close to shore near Shishmaref. The killer whales may have taken the calf.

Hunters can push whales in the direction they want them to go by slapping the water with their paddles. This imitates the behavior of killer whales, which slap their tails and dorsal fin on the water to scare the whales and push them in the direction the killer whales want them to go. Whales have occasionally come into Shishmaref Lagoon, but this has not happened in recent years. The bones of a whale can be found a short distance upstream from the mouth of the Serpentine River, where hunters pursued it many years ago. It is not known what species of whale it was.

Shishmaref hunters took a bowhead whale once, when it came closer to the shorefast ice than usual.

Many more gray whale carcasses are seen along the coast near Shishmaref than there used to be. Bowhead whale carcasses remain rare.

Fish arrive in spring after the ice goes out.

There are more seabirds in the Shishmaref area now than there used to be. There are also different birds

There are different crabs in the Shishmaref area in recent years.

Some hunters will print satellite images of sea ice before going out on the shorefast ice in spring.

The channels into Shishmaref Lagoon formed relatively recently. The one to the north of the village, for example, opened within the past two generations. The grandmother of one Shishmaref resident described jumping over the narrow channel when she was young, as it was forming.

Caribou are found closer to the village recently than they have been for many years. The warmer weather may be an influence. The elders said the caribou would return to this area. They can be seen on the mainland not far from Shishmaref. It used to be that only a few caribou might be seen, but now there are hundreds if not thousands. Predators are closer, too, including wolves, wolverine, and brown bears.

It used to be possible to predict good weather for a couple of days, so that hunters could cross Kotzebue Sound for example. Now, the winds and bad weather can come up very quickly.

Acknowledgements
We are grateful for the skill, expertise, and generosity of the five hunters who participated in the interviews. We appreciate the support of the Eskimo Walrus Commission and the Ice Seal Committee for this project and are grateful to Jane Kakoona, Karen Olanna, and Renee Kuzuguk from the Shishmaref IRA Council office for helping to set up the interviews. The Bureau of Ocean Energy Management (BOEM) funded the work as part of Contract Nos. M09PC00027 and M13PC00015 and we appreciate the support of Charles Monnett, Catherine Coon, Dan Holiday, and Carol Fairfield. Justin Crawford prepared the maps used during the interviews and the figure in this report.

References

Huntington, H.P. 1998. Observations on the utility of the semi-directive interview for documenting traditional ecological knowledge. Arctic 51(3):237-242.

Noongwook, G., the Native Village of Gambell, the Native Village of Savoonga, H.P. Huntington, and J.C. George. 2007. Traditional knowledge of the bowhead whale (*Balaena mysticetus*) around St. Lawrence Island, Alaska. Arctic 60(1):47–54.
